# Emotional and Social Dimension of Abstract Concepts Meet with Interoception in Right Anterior Insula

**DOI:** 10.1523/JNEUROSCI.0238-25.2025

**Published:** 2025-11-21

**Authors:** Martina Mancano, Costanza Papagno

**Affiliations:** ^1^Center for Mind/Brain Sciences, University of Trento, Rovereto 38068, Italy; ^2^CISMed Interdepartmental Center for Medical Sciences, University of Trento, Trento 38122, Italy

**Keywords:** abstract concepts, anterior insula, emotions, interoception, semantics, TMS

## Abstract

According to the embodied cognition theory, which claims that concepts’ representation is grounded in sensory and motor components, abstract concepts are grounded in interoception, which is processed in the Anterior Insula (AIns). However, it is not clear whether interoception and abstract concepts share common anatomical substrates, and if yes, whether AIns is one candidate. In this study, we used repetitive transcranial magnetic stimulation (rTMS) on healthy human participants (*N* = 25, 19 females) to examine whether left and right AIns play a role in both abstract concepts and interoception. The heartbeat counting task served as a measure of interoceptive accuracy, and the semantic similarity judgment as a measure of semantic performance. Concepts were characterized according to both a categorical approach, contrasting three categories of concepts, namely social and emotion (abstract categories) and objects (concrete category), and a dimensional approach, collecting semantic ratings on the emotion and social dimensions of abstract and concrete concepts. TMS site and TMS-induced electric field inside the AIns ROI were used to predict interoceptive and semantic behavioral responses. Both TMS site and E-field ROI analyses confirmed the right AIns' role in supporting interoception and the emotion and social dimensions of abstract concepts. This aligns with an embodied cognition framework, where AIns is involved in both the nonlinguistic and linguistic processing of emotional and social dimensions. Together, these results support the evidence of a relation between interoception and socio-emotional semantics and the convergence of these two processes on the right AIns.

## Significance Statement

Humans’ awareness of their internal, physiological states enables them to perceive their emotions and navigate the social environment successfully. However, little is still known on how the meaning of words we use to refer to emotions and social events is rooted in our bodily awareness. In the present study, we show that the right anterior insula, a crucial region for interoception, causally supports the representation of the emotional and social dimensions of abstract words as well. These results demonstrate that there is a common region in the brain involved in both processes, stress the connection between anterior insula and emotional processing, and deepen our understanding of the connection between body and language.

## Introduction

Concrete concepts refer to external entities (e.g., car, dog) and abstract concepts to complex, introspective ideas (e.g., fear, problem).

Concrete concepts are usually processed significantly faster than abstract concepts, i.e., the so-called concreteness effect (Paivio et al., 1994). The dual-coding theory (Paivio, 1991) explains it claiming that abstract concepts are represented in a linguistic format only, while concrete concepts have both a linguistic and a perceptual representation.

Conversely, the embodied cognition claims that both concrete (Barsalou, 2008) and abstract concepts (Barsalou et al., 2018; Desai et al., 2018) are represented in the sensory and motor brain areas activated by the corresponding experiences.

While concrete concepts can be clearly divided in categories (Warrington and Shallice, 1984), only recently, a distinction in categories (e.g., social: “democracy”, quantity: “zero”) has been confirmed for abstract concepts, in healthy (Desai et al., 2018; Conca et al., 2021) and clinical populations (Mancano and Papagno, 2023).

The abovementioned research highlights Emotion as one important dimension of abstract concepts (Vigliocco et al., 2014). Abstract words are more emotionally valenced than concrete ones (Kousta et al., 2011), and emotionality facilitates processing of abstract more than of concrete words (Newcombe et al., 2012; Moffat et al., 2015; Siakaluk et al., 2016).

The experience of emotions is associated with physiological responses (e.g., heartbeat, visceral, and breathing; James, 1884) that we perceive through interoception, i.e., perception of the internal states (Craig, 2003; Critchley and Harrison, 2013). Interoception is processed in the bilateral insula (AIns), whereas interoceptive and salient stimuli are integrated into a unified representation of the bodily emotional state in the Anterior Insula (Critchley et al., 2004; Craig, 2009).

Interoceptive strength, i.e., how much a concept is experienced through interoception, is higher for abstract than concrete concepts and for emotional compared with neutral abstract concepts (Connell et al., 2018; Repetto et al., 2023). Words with low concreteness, or abstract words, elicit a higher change in heart rate (Vergallito et al., 2019). The difference in perceived difficulty between abstract and concrete words was larger when participants were at the same time monitoring their heartbeat rather than performing other noninteroceptive tasks, especially for emotional and social concepts (Villani et al., 2021). Convergent results from recent studies strengthen the association between interoception and abstract emotional word (Barca et al., n.d.; Ferré et al., 2024).

AIns is also involved in the representation of abstract concepts (Papagno et al., 2013; Cousins et al., 2016), particularly emotion concepts (Desai et al., 2018; Ziegler et al., 2018; Conca et al., 2021). However, we are not aware of studies investigating AIns in relation to both emotion words and interoception at the same time. Even though emotion and social have been distinguished as two different categories (Conca et al., 2021; Mancano and Papagno, 2023), they have been seldom directly contrasted.

**Figure 1.**
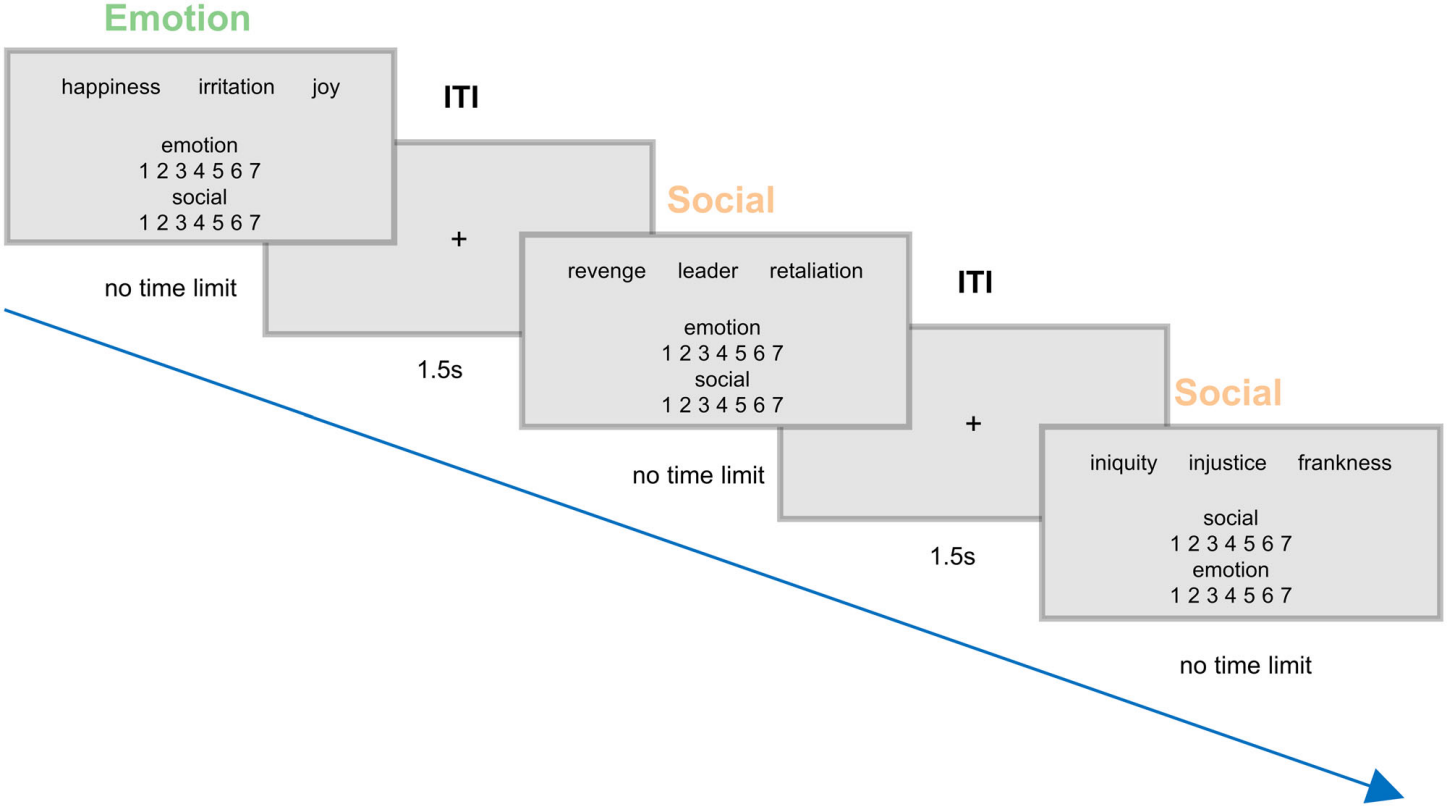
Semantic ratings study. Experimental procedure. Subjects were presented with triplets of words. In each trial, they were asked to evaluate how strongly, on a Likert scale ranging from 1 to 7, the meaning of the triplet words was associated to the emotion and social scales. They were asked to read all the three words before making their choice. Emotion scale was defined as how much a concept is used to express/evokes an affective state or an internal state that you can perceive. Social scale was defined as how much a concept is used to describe/is associated to an interpersonal relationship, an institution or a social construct.

**Figure 2.**
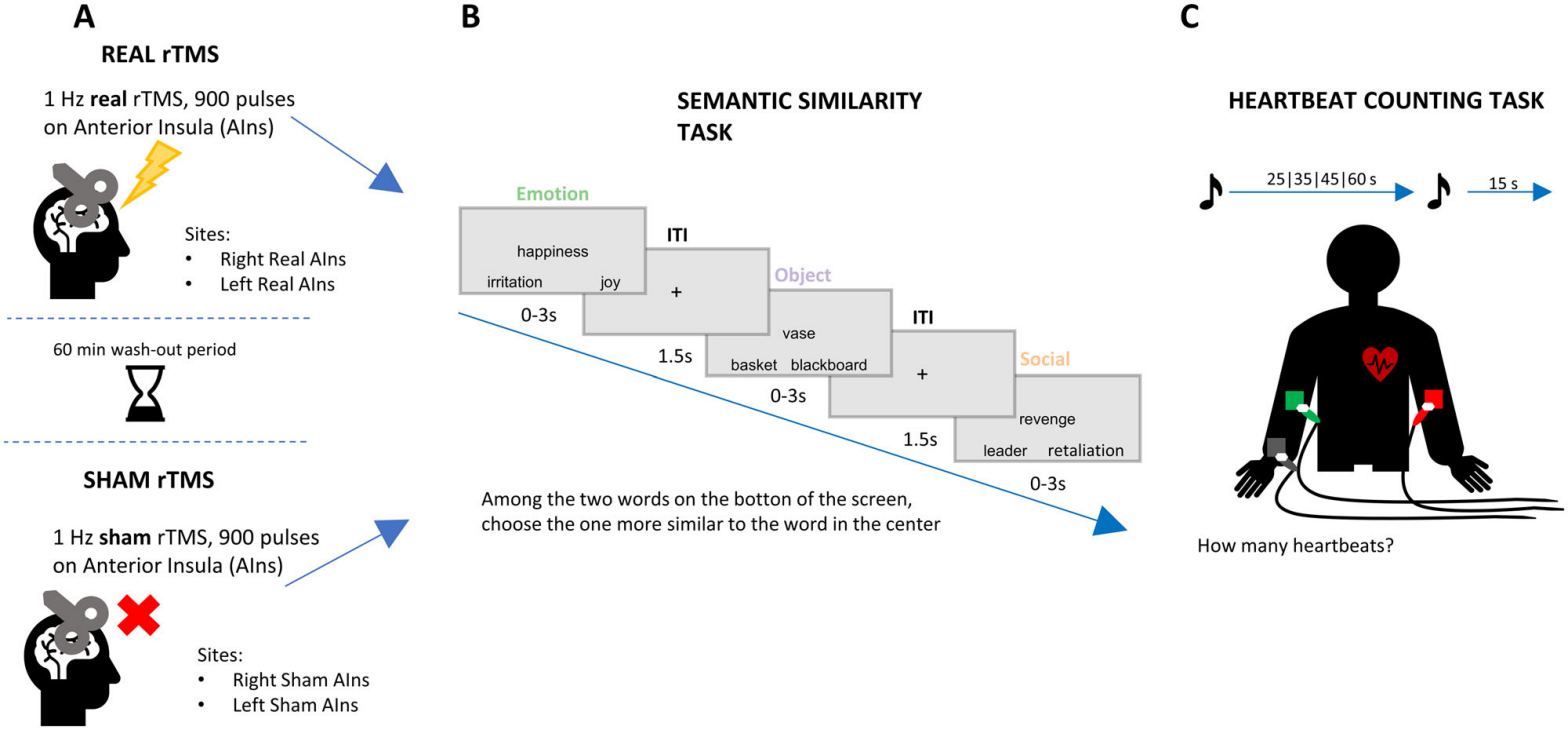
TMS experiment. Experimental procedure. Schematic representation of one TMS session. ***A***, TMS. Participants were firstly submitted to a 15 min (900 pulses) neuronavigated repetitive TMS (rTMS) session, consisting of one of the four experimental conditions: Right Real AIns, Right Sham AIns, Left Real AIns, Left Sham AIns. During sham conditions, a wooden eight-shaped block was placed between the coil and the scalp. The order of TMS conditions was counterbalanced across participants. Two tasks followed the stimulation: ***B***, Semantic similarity task. In this task, participants were presented with triplets of words. In each trial, they were asked to identify, among the two words at the bottom of the screen, the more similar word in meaning (“target”) to the word presented in the center (“probe”), by pressing respectively “A” and “L” on the keyboard, if the target word appeared on the left or the right side. ***C***, Heartbeat counting task. During the electrocardiogram (ECG) recording, subjects were asked to start silently counting their heartbeats at a signal sound and report how many heartbeats they perceived until they heard a second sound. Participants performed four blocks of variable duration in randomized order (25, 35, 45, and 60 s). The order of the tasks was counterbalanced across participants and sessions.

To address these points, we performed a behavioral study to obtain semantic ratings on the Emotion and Social dimensions of concepts (Fig. 1). Then, we used repetitive transcranial magnetic stimulation (rTMS) to investigate the role of bilateral AIns in the representation of concrete and abstract concepts and interoception (Fig. 2).

We considered Objects, Emotion, and Social categories based on a categorical approach. We also adopted a dimensional approach (Crutch et al., 2013; Troche et al., 2017), considering both the Emotional and Social dimensions of concepts.

We predicted an interference on interoception after stimulation of right AIns, but not left AIns (Critchley et al., 2004; Pollatos et al., 2007a, 2016; Mai et al., 2019).

For the semantic results, although “linguistic” regions are typically left-lateralized (Binder et al., 2009), emotional language deficits follow right brain damage (Sidtis and Sidtis, 2018; Sheppard et al., 2022). Based on the literature linking interoception and emotion concepts (Connell et al., 2018; Villani et al., 2021), and their partial overlap with social ones, we expected an effect either (1) restricted to the Emotion or (2) extended to both the Emotion and the Social features.

## Materials and Methods

### Experiment 1—semantic ratings

Abstract concepts are not as easily classifiable into distinct categories as concepts referring to concrete, tangible entities. They can evoke multiple meanings and are more difficult to characterize.

Therefore, in the first preliminary behavioral study, we asked participants to provide semantic ratings of the emotion and social dimension of experience on the abstract stimuli in our dataset. In a second data collection phase, we obtained ratings on the same emotion and social dimensions on concrete stimuli as a control.

#### Participants

Twenty-one participants (10 males and 11 females, mean age: 25.23 years, SD = 2.94, range = 19–30) took part in the first data collection phase. All participants were right-handed, Italian native speakers. One was removed from the analysis due to performance in the experimental task >2 SD below the group mean in all conditions. Thus, the final sample comprised 20 people (10 males and 10 females, mean age: 25.27 years, SD = 2.88, range = 19–30). Twenty participants (14 females, mean age: 27.95 years, SD: 3.44, range: 22–35) took part in the second data collection phase, all right-handed Italian native speakers. All participants gave their written informed consent before starting the experiment. The experiment was approved by the Research Ethics Committee of the University of Trento.

#### Methods

Our set of linguistic stimuli comprised triplets categorized as either objects (concrete triplets), emotion, or social concepts (abstract triplets; see below, Semantic similarity task, for a full description of linguistic stimuli). We collected these emotion and social ratings on the Social and Emotion triplets because, despite their categorization based on the concepts they referred to, the distinction was sometimes fuzzy as words belonging to one of the two categories (i.e., social concepts, e.g., “friendship”) often displayed a certain degree of features from the other category (e.g., “friendship” having an emotional connotation). We also collected ratings on the Object triplets as a control. Since we included mostly everyday objects that should not consistently elicit an emotional response or be associated to a social interaction, we did not expect these items to present with significative scores on these dimensions.

In each trial, subjects were required to rate on a 7-point Likert scale the meaning of the triplets’ words on two dimensions: the Emotion scale, i.e., how much a concept refers to/evokes an affective state, and the Social scale, i.e., how much a concept refers/is associated to relationships between people or social constructs. Participants were instructed to read all three words before expressing their judgment, which thus referred to the whole triplet. Triplets appeared in the center of the screen, and the two scales on the bottom half of the screen, one on top of each other. All triplets were rated on both scales by all participants. They were instructed to press the numerical keyboard button corresponding to their rating on the first scale and then the numerical keyboard button corresponding to their rating on the second scale. Participants were given no time limit to make their choice. The order of the scales in each trial was randomized. Each trial began with a fixation cross at the center of the screen (1,500 ms), followed by the presentation of the triplet for 1,000 ms, after which the two scales appeared (no time limit; Fig. 1).

#### Analysis

We analyzed mean category and by-triplet Social and Emotion ratings. Analyses were performed on MATLAB 2021b.

We first calculated average Emotion and Social ratings for each triplet. For each triplet, we eliminated outliers above or below two SD from each triplet's average rating on the respective scale (0.047% of ratings on the social dimension and 0.044% of ratings on the emotion dimension) and then re-calculated mean category Emotion and Social ratings without outliers. One-way ANOVAs and post hoc tests were performed to test whether mean emotion and social scores differed between triplets categorized as either Emotion, Social, or Objects concepts.

For abstract concepts, we also checked for triplets with ambiguous features, i.e., rated below 4.5 on the corresponding scale or above 4 on the other category scale (e.g., an Emotion triplet that scores below 4.5 average rating on the Emotion scale, or above 4 average rating on the Social scale and vice versa). We found 14 triplets with ambiguous features and replaced them with other items, which we did not include in the semantic ratings analysis of abstract concepts.

### Experiment 2—TMS experiment

In our main TMS experiment, we used repetitive TMS to disrupt activity in right and left AIns, to test its role in semantic processing and interoception. After 15 min of real (inhibitory) or sham rTMS, participants performed two tasks in counterbalanced order: a heartbeat counting task, to test their interoceptive accuracy, and a semantic similarity task, to test their semantic performance (Fig. 2).

#### Participants

Twenty-six healthy Italian participants (7 males, 19 females, mean age 23.15 years, range 20–30, SD 3.00) took part in the TMS experiment. Inclusion criteria were the following: right-handed, Italian native speakers, no history of psychiatric, neurological, or developmental disorders. No subject who participated in the semantic ratings experiment took part in the TMS experiment. We excluded one participant due to technical problems, so the final sample comprised 25 subjects (6 males, 19 females, mean age 23 years, range 20–30, SD 2.95). We recruited, through advertisement at the University of Trento, Italy, participants whose magnetic resonance (MRI) had already been acquired at CIMeC, University of Trento. They received a token of €60. All participants gave their written informed consent before starting the experiment. The experiment was approved by the Research Ethics Committee of the University of Trento.

#### Methods

The experiment consisted of two sessions, in which the right and left AIns were targeted (Fig. 2). In each session, we administered both a real and a sham stimulation, separated by a washout period of 60 min. We used a repeated measures design where all participants underwent all stimulations and the sequence of sessions was counterbalanced across participants [four possible sequences: (1) day 1: Left Insula Real-Left Insula Sham; day 2: Right Insula Sham-Right Insula Real, (2) day 1: Left Insula Sham-Left Insula Real; day 2: Right Insula Real-Right Insula Sham, (3) day 1: Right Insula Real-Right Insula Sham; day 2: Left Insula Sham-Left Insula Real, (4) day 1: Right Insula Sham-Right Insula Real; day 2: Left Insula Real-Left Insula Sham].

At the beginning of each session, participants performed a first heartbeat counting task (baseline). After that, there was a short training phase for the semantic similarity task, where participants made semantic decisions on a set of training triplets. We did not explain to participants that triplets belonged to three categories either in the training phase or in the experiment. Subsequently, in the first session, the resting motor threshold was acquired, whereas in the second one, we started directly with the 15 min rTMS stimulation. Subjects performed two tasks immediately after rTMS: the heartbeat counting and the semantic similarity task. The order of the tasks was counterbalanced across participants and sessions. The completion of both tasks took ∼10 min. After concluding the second task, there was a break of ∼50 min (60 min of washout minus the 10 min of tasks), before starting the second stimulation condition (real or sham). After this second stimulation of each day, participants performed the same two tasks, in the same order as after the first stimulation (i.e., if participants had started with the semantic task in the first stimulation of the day, they would start with the semantic also in the second one). In the following session, after 2 weeks, they performed the two tasks in the opposite order (i.e., if they had started with the semantic task the first day, they would start with the heartbeat counting task on the second day and vice versa).

The first session lasted ∼2 h and 20 min, and the second one lasted 2 h.

##### TMS paradigm

Neuronavigated rTMS was delivered using a MagPro X100 with MagOption connected to a MagVenture MCF-B65 coil, using the SofTaxic navigator system software (SofTaxic, EMS).

Participants underwent rTMS on two different days, separated by 2 weeks (mean: 14 d, SD: 1.26). In each session, two 15 min rTMS at 1 Hz (900 pulses) were applied over one of two sites: right AIns or left AIns. Placement of the coil on the target area was accomplished through neuronavigation on each participant's individual MR T1.

Each day, the TMS conditions were a real and a sham stimulation, in counterbalanced order. In the sham stimulation, an 8-shaped wooden block, 3 cm thick, was placed between the coil and the scalp, to prevent the induced electric current from reaching the brain. Real and sham stimulations were separated by a washout period of 60 min (counted from the end of the first stimulation to the beginning of the second). In the following session, participants would start with the other condition (e.g., day 1: left real-left sham, day 2: right sham-right real).

The stimulation intensity was set at 90% of the previously determined resting motor threshold (RMT), which corresponded on average to 58.9% (SD = 9.89%) of maximum stimulator output (MSO). To determine the RMT, we first localized the left motor hand area delivering TMS pulses and inspecting for visible muscle twitches in the contralateral hand. We acquired the position of the coil that reliably elicited muscle twitches. With the coil placed in that position, to identify the RMT, we used the software TMS Motor Threshold Assessment Tool, MTAT 2.1 with the option set to “without a priori information.” A trial was considered successful when a visible muscle twitch was induced in the index finger of the contralateral hand.

For each participant, we manually selected the target area in the dorsal-rostral part of the first gyrus of the anterior insula, corresponding approximately to [(−)36, 18, 0] MNI coordinates. We positioned the coil on the participant's head based on online navigated TMS and stabilized it with mechanical support.

##### Heartbeat counting task

The heartbeat counting task consisted of four counting phases that lasted 25, 35, 45, or 60 s, in randomized order, with a fixed interval of 15 s between sessions. A sound signaled the beginning (1,200 Hz) and the end (300 Hz) of each session. With their eyes closed, subjects were asked to silently count their heartbeats during the sessions and to verbally report at the end of each counting session how many heartbeats they had felt.

Meanwhile, the ECG was recorded with electrodes placed on the right (negative) and left (positive) upper forearm, and ground on the right wrist, connected to an EKG sensor (https://www.vernier.com/product/ekg-sensor/) and SensorDAQ data acquisition interface (https://www.vernier.com/product/sensordaq/). We used MATLAB Support Package for Vernier SensorDAQ to access and store data. ECG data were recorded with a sampling rate of 250 Hz. The presentation of the task was governed through Psychophysics Toolbox version 3 (PTB-3; Kleiner et al., 2007) in MATLAB.

During the task, participants were comfortably sitting on a chair, with their palms facing up and their forearms resting on the table.

The first time that they performed the task in each session (pre-TMS or baseline), they were presented with the instructions, where they were explicitly told to report only heartbeats that they felt, without trying to guess their heart rate. This could mean reporting all heartbeats, some heartbeats, or no heartbeats at all. We formulated the instructions following Desmedt et al. (2020) study, to minimize the risk that participants based the number of reported heartbeats on time estimates. We added these baselines to the heartbeat counting task in both sessions (baseline day 1, baseline day 2) to familiarize participants with the task and prevent possible confounding practice effects (Sagliano et al., 2019). When they performed the tasks after TMS administration, instructions were not repeated.

##### Semantic similarity task

In this task, subjects were presented with triplets of nouns (180 triplets, 540 words). Each trial consisted of one triplet: a probe word appeared in the center of the screen (e.g., “crime”) and two other semantically related words at the bottom right and bottom left—one was the target word (semantically similar to the probe, e.g., “offence”) and the other a semantically distant word (semantically distant from the probe word, e.g., “friendship”).

The side of the target and distant word were randomized. In each trial, subjects were required to select the target word.

Triplets belonged to three semantic categories: Emotion, Social (i.e., the abstract categories), and Objects (i.e., the concrete category), with 60 triplets for each category.

For Object concepts, we selected words referring to man-made objects, mostly manipulable objects (e.g., “forbici,” scissors; “scopa,” broom), but also a few non-manipulable objects were included (e.g., “armadio,” closet).

For Emotion concepts, we selected words directly referring to an emotional state/internal feeling (e.g., “euforia,” euphoria; “timore,” fear).

For Social concepts, we selected words referring to interpersonal relationships/actions (e.g., “amicizia,” friendship; “aggression,” aggression) and societal/cultural concepts (e.g., “penalità,” penalty).

The abstract categories were also rated on both the emotion and the social dimensions (see above, Experiment 1—Semantic ratings), so that an emotion triplet (made of emotion-referring words) had a score on both the emotion and the social continuous dimensions, and a social triplet (made of social-referring words) had a score on both dimensions as well.

Words were initially drawn from Villani et al. (2019), Montefinese et al. (2014), and Della Rosa et al. (2010) datasets, but new words were used to achieve the necessary number of stimuli and balance across categories. All stimuli are available on the Open Science Framework (OSF) project page associated with this study (https://osf.io/cavsy/).

The main task was preceded by a short training phase before TMS, during which participants were presented with 12 different triplets (4 triplets for each category) to familiarize themselves with the task.

The order of triplet presentation was randomized, and triplets belonging to the different categories (Emotions, Social concepts, Objects) were interleaved. Words were balanced across categories in terms of Subtlex-it log frequency (https://osf.io/zg7sc/; *F*_(2,537)_ = 1.86, *p* = 0.157) and controlled for length (*F*_(2,537)_ = 10.04, *p* ≤ 0.001). We could not balance for length, in that objects were significantly shorter than both abstract categories (Objects-Emotions: *T* = −4.19, *p* ≤ 0.0001, Objects-Social: *T* = −3.467, *p* = 0.001).

We used Word-Embeddings Italian Semantic Space (Marelli, 2017), a word2vec model trained on Italian text corpora, to extract cosine similarity between pairs of words in our dataset. Stimuli were balanced for semantic similarity between probe-target words (“semantic similarity similar”; *F*_(2,177)_ = 2.09, *p* = 0.126) and semantic similarity between probe-distant words (“semantic similarity distant”; *F*_(2,177)_ = 2.33, *p* = 0.1) across categories.

The presentation of task stimuli was performed using Psychophysics Toolbox version 3 (PTB-3; Kleiner et al., 2007) on MATLAB.

Each trial began with a fixation cross at the center of the screen (1,500 ms), followed by the triplet presentation. Subjects had a time limit of 3 s to respond. They were instructed to press the “A” keyboard button when the target word was presented on the left side and the “L” button when it appeared on the right side. After they responded to the stimulus, the script automatically moved to the next trial. If no answer was provided, after the maximum time (3 s), the next trial appeared. Reaction times (RTs) and accuracy were recorded.

In each session, half of the stimuli (90 triplets, 30 per category) were presented after the first stimulation and the other half after the second stimulation. The splitting of the dataset was randomized for each session and each participant, balancing for probe frequency and length, target frequency and length, distant frequency and length, semantic similarity similar, and semantic similarity distant between the two halves (all *p* > 0.06).

#### Analyses

##### Heartbeat counting task

Interoceptive accuracy. In the ECG data, R-wave peaks of recorded heartbeats were detected using “findpeaks” MATLAB function. Interoceptive accuracy was calculated with the following formula:

1/4∑(1−(|recordedheartbeats−countedheartbeats|)/recordedheartbeats),

whereby scores range continuously between 0 and 1, with higher scores indicating better performance (i.e., higher accuracy). We also computed the average Heart Rate during the counting sessions.

The following analyses were conducted using R-Studio (version 4.3.1). Outliers detected using the “boxplot.stats” function were excluded from the analysis (3 observations above the third quantile + (1.5 × IQR)).

Interoceptive accuracy was analyzed with generalized linear mixed models (GLMMs), fitting a model with beta family and link function logit, computed using the “glmmTMB” package (Magnusson et al., 2017) and “lmerTest” package (Kuznetsova et al., 2017) for *p* values. Beta distribution is a distribution used to analyze continuous proportional data ranging in the interval 0–1. An approach based on GLMMs was used to account for the effects of repeated measures within subjects. Even though it is recommended to use the maximal random effect structure (Barr et al., 2013), adding the random by-subjects slopes led to convergence issues, so we used a random intercept model.

Interoceptive accuracy was modeled with TMS session (6 levels: Baseline 1, Baseline 2, Left Insula Real, Left Insula Sham, Right Insula Real, Right Insula Sham) as fixed effect, Heart Rate as control covariate, and by-subject random intercept as a random effect. The model formula (R syntax) was the following:

accuracy∼TMS_session+Heart_Rate_overall+(1|ID_subject).

In this and all the following models, continuous predictors and covariates were always centered.

Model assumptions were tested with “DHARMa” package (Hartig and Hartig, 2017) and did not reveal any significant problems.

Estimated marginal means (EMMs) were calculated with the “emmeans” package (Lenth and Lenth, 2018). We applied planned comparisons to test for contrasts of interest; specifically, we tested for differences in interoceptive accuracy between Left Insula Real-Left Insula Sham, and differences between Right Insula Real-Right Insula Sham. The results were adjusted for multiple comparisons using the “Holm” method.

##### Semantic similarity judgment

Analyses were conducted using R-Studio (version 4.3.1). Single-trial RTs and accuracy were analyzed with linear mixed models (LMMs) and generalized linear mixed models (GLMMs). Mixed models were chosen to account for the effects of repeated measures within subjects and trials. Adding the random slopes led to convergence problems; therefore, we only included by-subjects and by-triplet random intercepts.

(1) Category. In this first analysis, we adopted a categorical approach, which aimed to investigate the effect of the interaction between TMS site and semantic categories in predicting semantic processing.

RTs. The histogram of raw RTs and QQ-Plot suggested that RTs were not normally distributed and presented a right-skewed distribution. Therefore, we run all the following analyses with log-transformed RTs as the dependent variable. RTs faster than 300 ms and RTs for incorrect trials were excluded from the analysis (646 incorrect trials, 7.17%).

Log RTs were analyzed with linear mixed models (LMMs), computed using the “lme4” package (Bates et al., 2015a) for modeling and “lmerTest” package for *p* values (Kuznetsova et al., 2017).

Log RTs were modeled with TMS site (four levels: Left Insula Real, Left Insula Sham, Right Insula Real, Right Insula Sham), category (three levels: Emotions, Objects, Social), and their interaction, as fixed factors; semantic similarity similar, semantic similarity distant, and triplet length as control covariates; by-subject and by-triplet random intercepts as random effects. The model formula (R syntax) was the following:

log(RTs)∼TMS_session×category+semanticSimilaritySimilar+semanticSimilarityDistant+tripletlength+(1|ID_subject)+(1|IDTriplet).

Triplet length was calculated as the sum of letters of all three words in the triplet.

Model assumptions were visually inspected with the plot_model function from “sjPlot” package (Lüdecke, 2023) and did not reveal any large deviation from normality or homoscedasticity.

Estimated marginal means (EMMs) were calculated with the “emmeans” package (Günther et al., 2015). We applied planned comparisons to test for contrasts of interest, namely, the following 6 TMS site × category comparisons: Emotions Left Insula Real-Left Insula Sham, Social Left Insula Real-Left Insula Sham, Objects Left Insula Real-Left Insula Sham, Emotions Right Insula Real-Right Insula Sham, Social Right Insula Real-Right Insula Sham, Objects Right Insula Real-Right Insula Sham. The results were adjusted for multiple comparisons using the “Holm” method.

Accuracy. Accuracy was analyzed with generalized linear mixed models (GLMMs), with family binomial and link function logit, using “lme4” package (Bates et al., 2015b) for modeling and “lmer-test” package (Kuznetsova et al., 2017) for *p* values.

Accuracy was modeled with the same fixed and random effects specified for RTs. The model formula (R syntax) was the following:

Accuracy∼TMS_session×category+semanticSimilaritySimilar+semanticSimilarityDistant+tripletlength+(1|ID_subject)+(1|IDTriplet).

Model assumptions were tested with “DHARMa” package (Hartig and Hartig, 2017) and did not reveal any significant problems.

Estimated marginal means (EMMs) were calculated with the “emmeans” package (Lenth and Lenth, 2018). We applied planned comparisons to test for contrasts of interest; specifically, we tested for the same TMS × category comparisons described for RTs.

(2) Semantic ratings. In a second analysis, we adopted a dimensional approach, using the semantic ratings collected in the first behavioral experiment (see above, Experiment 1—Semantic ratings). Due to a substitution of triplets with ambiguous features, ratings were available for 106 out of the 120 abstract triplets. As our main analysis, we tested the interaction between TMS site and the Emotion and Social scale ratings on abstract triplets. As a control, we repeated the same analysis on concrete triplets. If the effects of ratings and TMS targeting AIns are specific to the abstract concepts, we should find significant results in the first analysis with abstract triplets, whereas with concrete triplets we should not observe significant results.

RTs. Log RTs were modeled with TMS site (four levels: Left Insula Real, Left Insula Sham, Right Insula Real, Right Insula Sham), Social rating and Emotion rating, the interaction between TMS and Social rating, and between TMS and Emotion rating, as fixed effects; semantic similarity similar, semantic similarity distant, and triplet length as control covariates; and by-subject and by-triplet random intercepts as random effects. The model formula (R syntax) was the following:

log(RTs)∼(Emotion_rating+Social_rating)×TMS_session+semanticSimilaritySimilar+semanticSimilarityDistant+tripletlength+(1|ID_subject)+(1|IDTriplet).

Model assumptions were visually inspected with the plot_model function from “sjPlot” package (Lüdecke, 2023) and did not reveal any large deviation from normality or homoscedasticity.

Estimated marginal means of linear trends were calculated with “emmeans” package (Lenth and Lenth, 2018) for TMS site-Emotion rating and TMS site-Social rating interaction, to estimate the slope of the effect of the Emotion or Social rating at each TMS level. We applied planned comparisons to test for differences in Social and Emotion rating slopes between Left Insula Real-Left Insula Sham and between Right Insula Real-Right Insula Sham. The results were adjusted for multiple comparisons using the “Holm” method.

Accuracy. The model formula was:

Accuracy∼(Emotion_rating+Social_rating)×TMS_session+semanticSimilaritySimilar+semanticSimilarityDistant+tripletlength+(1|ID_subject)+(1|IDTriplet).

Model assumptions were tested with “DHARMa” package (Hartig and Hartig, 2017) and did not reveal any significant problems. Estimated marginal means of linear trends and planned comparisons were calculated in the same way described for RTs.

##### E-field modeling

**Figure 3.**
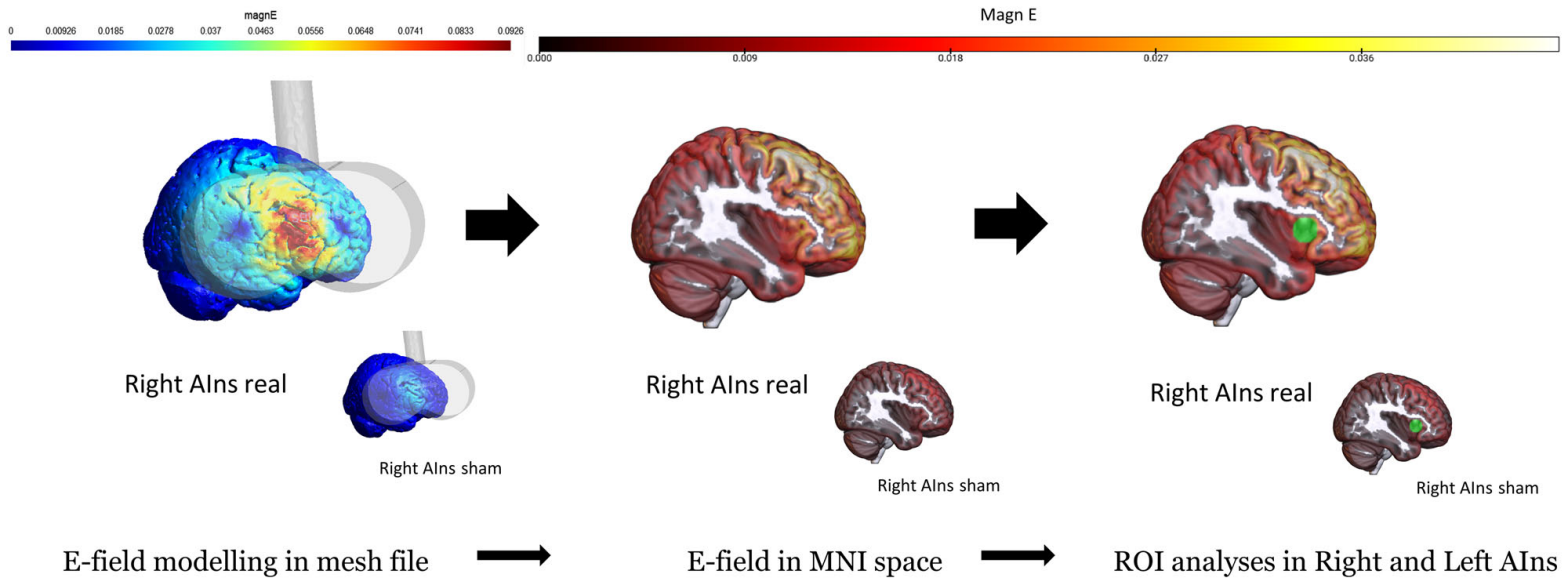
E-field modeling. E-field analysis pipeline. Analyses are conducted in SimNIBS 4.0. E-field is first simulated inside a head mesh model built from individual participants’ T1. Based on the focus of the coil coordinates, and the stimulation intensity, a propagation of the TMS-induced electric field in the brain is modeled for each subject in each TMS condition. These simulations are then interpolated to standard MNI space, where spherical, gray matter ROI centered around left and right AIns, left and right E-field maximum peak, and left and right PHC are created. E-field values from inside these ROI are then averaged to obtain the mean intensity (MagnE) of the E-field inside that specific area. These latter will be used in GLLMs to predict participants’ behavioral responses. See Extended Data Figure 3-1 for single-subject and average MNI coordinates of left and right maximum E-field peaks.

Simulations were performed in SimNIBS 4.0 (Thielscher et al., 2015). Individual T1-weighted structural MRI scans were segmented and meshed into tetrahedral head models through the *charm* pipeline (Fig. 3). Simulations of the TMS-induced E-field were performed in MATLAB environment. The TMS focus coil position was extracted from the *stmpx* files saved after each session from SofTaxic, with the handle pointing toward F8 for stimulation of right AIns and F7 for left AIns. The stimulation intensity was determined using the *dI/dt* (speed of variation of the current through the coil) value displayed directly on the TMS machine screen. E-field values are expressed in volts per meter (V/m). To simulate the TMS-induced E-field in the sham condition, we used the same parameters as in the real condition except for the distance of the coil from the scalp, which was set to 31 mm. The E-field in the sham condition, indeed, has much lower values compared with the real condition, but still it tells us that a very low intensity stimulation reached the brain. Mean E-field MagnE component was evaluated in ROIs defined in the MNI space (Van Hoornweder et al., 2023). Left and right dorsal Anterior Insula MNI coordinates were selected with an independent approach, where peak cluster activations were obtained from NeuroSynth “anterior insula” association maps (https://neurosynth.org/analyses/terms/anterior%20insula/). The resulting peaks MNI coordinates (right AIns: [40, 18, 2], left AIns: [−34, 22, 0]) were chosen as the center of spherical gray matter ROIs (*r* = 10 mm). E-field values (mean MagnE) were extracted via the mni2subject_coords function within these spheres in individual spaces. Because of technical problems (i.e., the simulation of the E-field in one subject could not be aligned to the MNI space), we removed this participant from this analysis.

To test whether the effects observed for interoceptive accuracy in the Heartbeat Counting task and RTs and accuracy in the semantic similarity task of the previous analyses were ascribable to TMS-induced E-field in AIns, we ran all previous GLMMs analyses, substituting TMS site categorical predictor with subject-level E-field values calculated inside AIns as continuous predictors (E-field value in right AIns, E-field value in left AIns; Zhang et al., 2022; Martin et al., 2025). To make an example, the equivalent of Category × TMS site analysis for RTs model formula (R syntax) was the following:

log(RTs)∼E-fieldMagnErightAIns×category+semanticSimilaritySimilar+semanticSimilarityDistant+tripletlength+(1|ID_subject)+(1|IDTriplet).

E-field MagnE right AIns in the formula is the mean electric field magnitude induced in the right AIns ROI. For the category analysis, estimated marginal means of linear trends were calculated, and planned comparisons were run to test for specific contrasts between the effect of E-field AIns and category level: Emotion-Social, Emotion-Objects, and Social-Objects.

Since this predictor variable represents the mean intensity of the E-field induced specifically inside AIns, and therefore the level of interference induced in this area by TMS, its predictive value of behavioral responses provides a measure of the relevance of AIns in sustaining those responses.

As a control analysis, we tested whether E-field calculated in an ROI centered around the E-field maximum peak in the brain predicted behavioral responses, and if yes, whether it provided a better fit for the data compared with E-field elicited inside right and left AIns ROI. We also did the same with another ROI, centered inside the parahippocampal gyrus (PHC), an area anatomically distant from the stimulation sites but involved in abstract and concrete concepts’ processing (Wang et al., 2010; Loiselle et al., 2012; Kafkas et al., 2023; Stochel and Sandberg, 2025), therefore an area that potentially, if stimulated, would have exerted an effect on the semantic similarity judgment task. We added these two areas to verify the specificity of the effects of the E-field evoked inside AIns on behavioral responses, namely, whether and how much these responses could be predicted by the E-field induced in other areas of the brain potentially involved.

For PHC, the MNI coordinates were ±22, −28, 16.

For E-field maximum peak (MAX E-field), MNI coordinates during right and during left TMS were extracted from single-subject E-field simulations during right and left TMS.

The same procedure used for AIns was also used to create a spherical gray matter ROI centered around the maximum peaks and one centered around PHC coordinates, yielding E-field average value in a sphere centered around the maximum left and the maximum right peak, and a sphere centered around right and left PHC.

Single-subject right and left MAX coordinates are shown in Extended Data Figure 3-1. The average peak in the right hemisphere was located in [57.48 30.56 16] MNI coordinates, the average peak in the left hemisphere in [−51.20, 32.12, 14.92] MNI coordinates, which correspond to pars triangularis of the left and right inferior frontal gyrus.

Then, GLMMs analogous to the ones with AIns E-field were computed using right and left MAX E-field as continuous predictors, to test whether the behavioral responses could be explained by the E-field elicited in the area of its peak intensity. The same was done using right and left PHC E-field.

Only when both models with AIns E-field and MAX E-field and/or PHC E-field presented a significant effect of interest, we compared the fit of the models to the data using the models’ log likelihood (loglik) and Akaike information criterion (AIC).

## Results

### Experiment 1—semantic ratings

**Figure 4.**
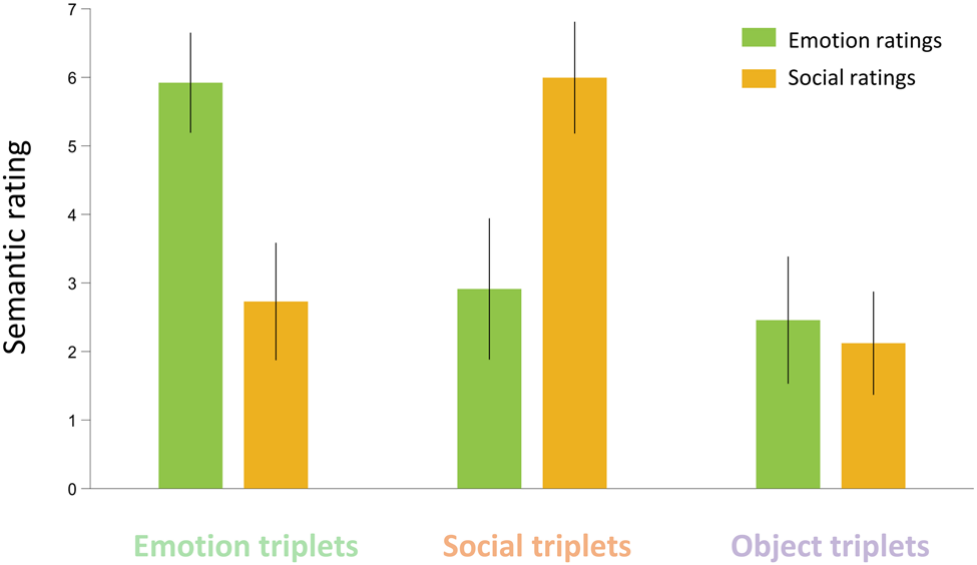
Semantic ratings experiment results. Average emotion (in green) and social (in orange) ratings for emotion triplets and social triplets, respectively. Error bars represent the standard deviation of the mean. Planned comparisons show both emotion and social ratings are significantly different across all three categories.

For Emotion triplets, mean Emotion and Social ratings (±SD) were as follows: Emotion ratings, 5.92 ± 0.73, Social ratings, 2.73 ± 0.86. For Social triplets, mean Emotion and Social ratings (±SD) were as follows: Emotion ratings, 2.91 ± 1.03, Social ratings, 6.00 ± 0.82. For Objects triplets, mean Emotion and Social ratings (±SD) were as follows: Emotion ratings, 2.46 ± 0.93, Social ratings, 2.12 ± 0.75. Emotion, Social, and Objects triplets differed significantly in their Emotion ratings (Objects-Emotions: *t*_(177)_ = −20.971, *p* < 0.001, Social-Emotions: *t*_(177)_ = −18.218, *p* < 0.001, Social-Objects: *t*_(177)_ = 2.753, *p* < 0.018) and in their Social ratings (Objects-Emotions: *t*_(177)_ = −4.114, *p* < 0.001, Social-Emotions: *t*_(177)_ = 22.094, *p* < 0.001, Social-Objects: *t*_(177)_ = 26.208, *p* < 0.001; Fig. 4).

Lower emotional and social ratings on concrete triplets confirm that these dimensions are not predominant in objects. While the higher average rating in the corresponding scale suggests that the categorical distinction between Emotion and Social concepts is valid, the moderate average rating on the other scale (i.e., social rating for emotion concepts and vice versa) indicates that a dimensional model captures more fine-grained features of abstract concepts representation.

### Experiment 2—TMS experiment

#### Heartbeat counting task

*Interoceptive accuracy*. TMS site (*Χ*^2^_(5)_ = 11,350, *p* = 0.045) significantly affected interoceptive accuracy, as did heart rate control covariate (*Χ*^2^_(5)_ = 11,273, *p* = 0.001), whereby higher heart rate predicted lower interoceptive accuracy.

**Figure 5.**
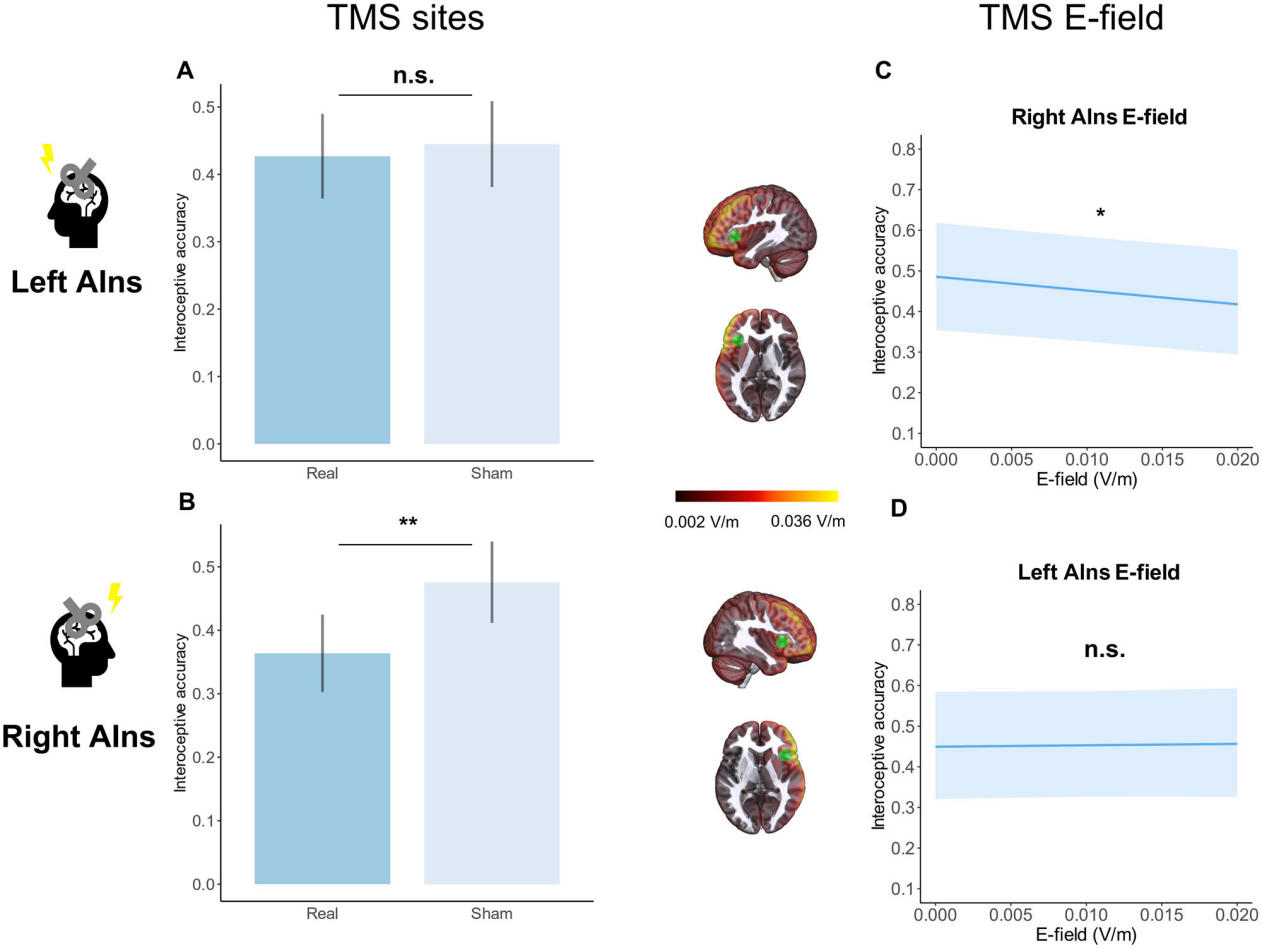
Heartbeat counting task results. AIns, anterior insula; E-field, electric field. ***A***, Estimated marginal means of interoceptive accuracy following left TMS conditions, converted from logit to the response scale. The comparison between left real-left sham is not significant. ***B***, Estimated marginal means of interoceptive accuracy following right TMS conditions, converted from logit to the response scale. The comparison between right real-right sham is significant: right real stimulation led to a decrease in performance, namely, to lower interoceptive accuracy. ***C***, Adjusted predictions of the effect of the E-field induced in left AIns on interoceptive accuracy. The effect of left AIns E-field is not significant. See ***A*** of Extended Data Figures 5-1 and 5-2 for the analysis with, respectively, left MAX E-field and left PHC E-field. ***D***, Adjusted predictions of the effect of the E-field induced in right AIns on interoceptive accuracy. The degree of right AIns E-field significantly lowers interoceptive accuracy: higher E-field values in right AIns lead to lower interoceptive accuracy. See ***B*** of Extended Data Figures 5-1 and 5-2 for the analysis with, respectively, right MAX E-field and right PHC E-field. ***A***, ***B***, Error bars represent standard errors of the marginal means (SEM). ***C***, ***D***, Error bars represent 95% confidence intervals (CI) of the adjusted predictions. ***A***, ***B***, *p* values of the planned comparisons were corrected for multiple comparisons using Holm correction. Tests are performed on the logit scale. **p < 0.01, *p < 0.05, n.s., *p* > 0.05.

Planned comparisons revealed a significant effect of the right AIns stimulation (Right Insula Real-Right Insula sham: *z* value = −2,940, *p* = 0.007), namely, participants’ interoceptive accuracy was lower after real versus sham right insula stimulation. Left insula stimulation, instead, did not significantly affect performance (Fig. 5*A*,*B*; Table 1).

10.1523/JNEUROSCI.0238-25.2025.f5-1 Figure 5-1 MAX E-field as predictor of Interoceptive accuracy MAX: maximum, E-field: electric field. **(A)** Adjusted predictions of the effect of the E-field induced in left MAX on interoceptive accuracy. The effect of left MAX E-field is not significant (Χ^2^ = 0.001, p = 0.970). **(B)** Adjusted predictions of the effect of the E-field induced in right MAX on interoceptive accuracy. The degree of right MAX E-field significantly lowers interoceptive accuracy: higher E-field values in right MAX led to lower interoceptive accuracy (Χ^2^ ^=^ 5.861, p = 0.015) **(A-B)** Error bars represent 95% confidence intervals (CI) of the adjusted predictions. Download Figure 5-1, TIF file.

10.1523/JNEUROSCI.0238-25.2025.f5-2 Figure 5-2 PHC E-field as predictor of Interoceptive accuracy PHC: parahippocampal cortex, E-field: electric field. **(A)** Adjusted predictions of the effect of the E-field induced in left PHC on interoceptive accuracy. The effect of left PHC E-field is not significant (Χ^2^ ^=^ 3.502, p = 0.061). **(B)** Adjusted predictions of the effect of the E-field induced in right PHC on interoceptive accuracy. The effect of right PHC E-field is not significant (Χ^2^ ^=^ 3.539, p = 0.060). **(A-B)** Error bars represent 95% confidence intervals (CI) of the adjusted predictions. Download Figure 5-2, TIF file.

**Table 1.** TMS site as predictor of interoceptive accuracy

	Chisq	Df	*p* value	
Model results				
(Intercept)	0.984	1	0.321	
TMS site	**11.350**	**5**	**0.045**	
Heart rate	**11.273**	**1**	**0.001**	
Contrast	odds.ratio	SE	*z*.ratio	*p* value
Planned comparisons				
Left Real-Left Sham	0.929	0.139	−0.491	0.624
Right Real-Right Sham	**0.630**	**0.099**	**−2.940**	**0.007**

Mixed-effect model results of TMS site predicting interoceptive accuracy and planned comparisons between ipsilateral real and sham stimulations, showing that right real stimulation significantly affected interoceptive accuracy compared with right sham stimulation. Significant results are written in bold. Chisq, chi-squared statistic; Df, degrees of freedom; SE, standard error; *z*.ratio, test statistic.

Crucially, E-field ROI analyses revealed consistent results. E-field values in the right AIns significantly affected interoceptive accuracy (MagnE Right AIns: *Χ*^2^ = 5.896, *p* = 0.015), namely, higher E-field values in right AIns predicted lower interoceptive accuracy. Heart rate also had a significant effect on interoceptive accuracy (*Χ*^2^ = 11.022, *p* = 0.001), with a higher heart rate predicting lower interoceptive accuracy. In contrast, E-field values in left AIns did not significantly affect interoceptive performance (Fig. 5*C*,*D*; Table 2; Extended Data Table 2-1).

**Table 2.** E-field in right anterior insula as predictor of interoceptive accuracy

	Chisq	Df	*p* value
Model results			
(Intercept)	0.506	1	0.477
Right AIns MagnE E-field	**5.896**	**1**	**0.015**
Heart rate	**11.022**	**1**	**0.001**

Mixed-effects regression model results of TMS E-field in right AIns predicting interoceptive accuracy, showing that the magnitude of E-field inside right AIns significantly affected interoceptive accuracy. Significant effects are written in bold. See Extended Data Table 2-1 for the analysis with left AIns E-field as predictor of Interoceptive accuracy. Chisq, chi-squared statistic; Df, degrees of freedom.

10.1523/JNEUROSCI.0238-25.2025.t1-1 Table 2-1 E-field in left Anterior Insula as predictor of Interoceptive accuracy. Mixed-effects regression model results of TMS E-field in left AIns as predictors of interoceptive accuracy. Significant effects are written in bold. Chisq: Chi-squared statistic, Df: degrees of freedom. Download Table 2-1, DOCX file.

E-field values in the right MAX E-field ROI also significantly affected interoceptive accuracy, namely, higher E-field values in right MAX predicted lower interoceptive accuracy (*Χ*^2^ = 5.861, *p* = 0.015). In contrast, E-field values in left MAX did not significantly affect interoceptive performance (*Χ*^2^ = 0.001, *p* = 0.970; Extended Data Fig. 5-1).

The E-field in right PHC does not have a significant effect on Interoceptive accuracy (*Χ*^2^ = 3.539, *p* = 0.060). The E-field in left PHC does not predict Interoceptive accuracy either (*Χ*^2^ = 3.502, *p* = 0.061; Extended Data Fig. 5-2).

Since both right MAX and right AIns exerted a significant effect on interoceptive accuracy, we compared the models’ fit to the data.

Right MAX ROI model loglik and AIC were, respectively, 56.992 and −103.985, and those of right AIns model were 56.995 and −103.989. The results indicate that right AIns model is a better fit (higher loglik, smaller AIC) than right MAX in explaining interoceptive accuracy data.

In brief, both TMS site and E-field analysis showed that stimulation applied over the right AIns significantly decreased interoceptive performance.

#### Semantic similarity task

##### Category

**Figure 6.**
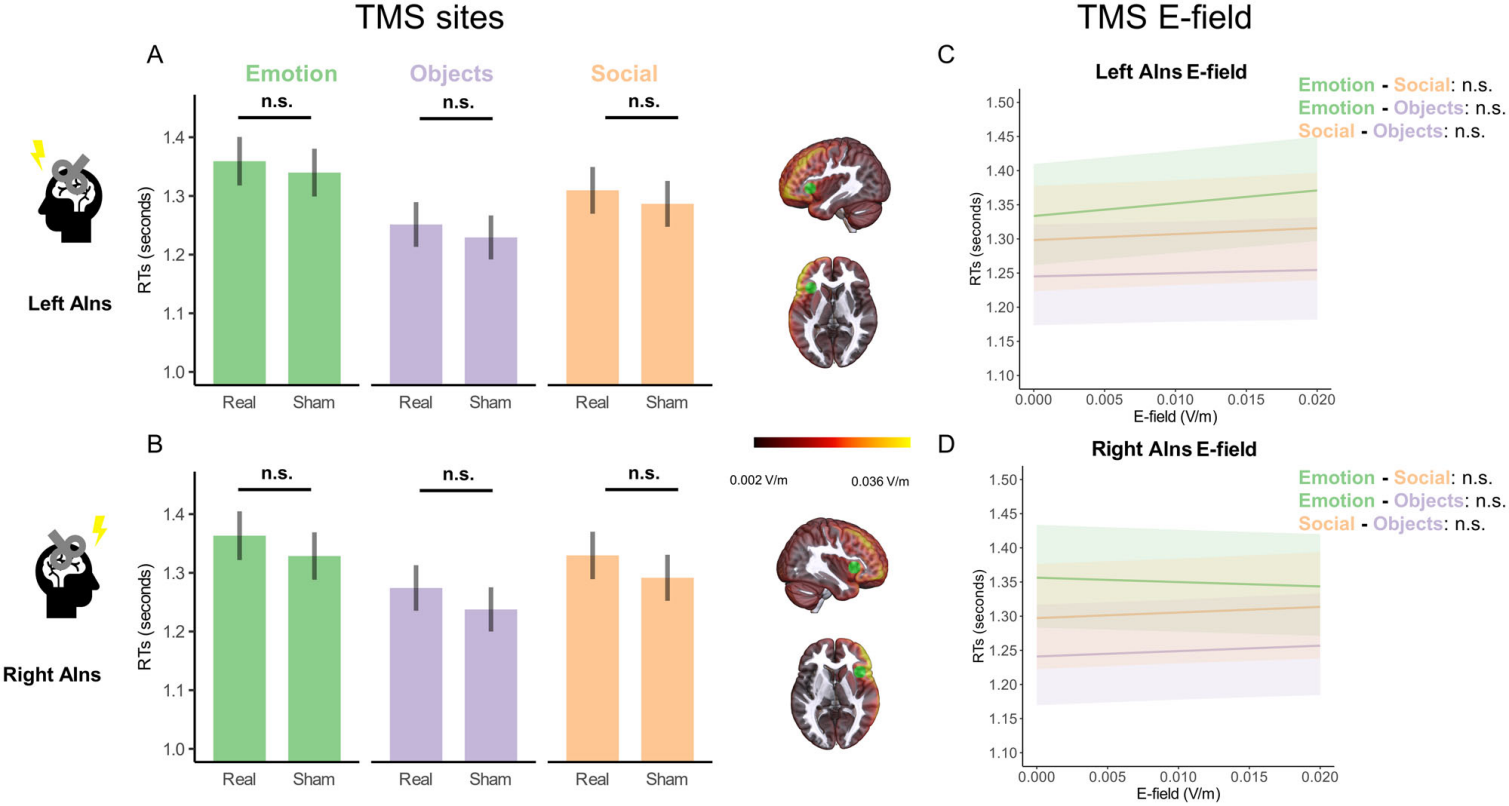
Semantic similarity task. Category results. RTs. AIns, anterior insula; E-field, electric field. ***A***, ***B***, Estimated marginal means of reaction times (RTs) following left (***A***) and right (***B***) TMS conditions, back-transformed from log to the response scale. Planned comparisons were nonsignificant (Extended Data Fig. 6-1). ***C***, Adjusted predictions of the interaction between the electric field (E-field) induced in left anterior insula (AIns) and category on RTs. Planned comparisons were nonsignificant (Extended Data Fig. 6-2). See ***A*** of Extended Data Figures 6-4 and 6-5 for the analysis with, respectively, left MAX E-field and left PHC E-field. ***D***, Adjusted predictions of the interaction between the electric field (E-field) induced in right anterior insula (AIns) and category on RTs. Planned comparisons were nonsignificant (Extended Data Fig. 6-3). See ***B*** of Extended Data Figures 6-4 and 6-5 for the analysis with, respectively, right MAX E-field and right PHC E-field. ***A***, ***B***, Error bars represent standard errors of the marginal means. ***C***, ***D***, Error bars represent 95% confidence interval (CI) of the adjusted predictions. All *p* values were corrected for multiple comparisons using Holm correction. n.s. *p* > 0.05. See Extended Data Figures 6-6–6-11 for the respective analysis with category and E-field in AIns, MAX and PHC as predictors of accuracy.

The main effect of TMS site (*F* = 6,994, *p* < 0.0001) and category (*F* = 9.817, *p* < 0.0001) were significant. The effects of the covariates semantic similarity similar (*F* = 10.294, *p* = 0.002) and triplet length (*F* = 5.745, *p* = 0.018) were also significant, whereas those of semantic similarity distant and the interaction between TMS and category were not significant. Planned comparisons were not significant (all *p* > 0.05; Fig. 6*A*,*B*; Extended Data Fig. 6-1).

10.1523/JNEUROSCI.0238-25.2025.f6-1 Figure 6-1 Interaction between category and TMS site as predictors of Reaction Times. Mixed-effects regression model results of TMS site and category as predictors of (log-transformed) reaction times, and planned comparisons between ipsilateral real and sham stimulations, showing for each semantic category the difference in average between right real and right sham TMS conditions, and between left real and left sham TMS conditions. Significant results are written in bold. Sum.Sq: Sum of squares, Mean.Sq: Sum of squares / degrees of freedom, NumDF, df: Degrees of freedom, DenDF: Denominator degrees of Freedom, estimate: estimated value of the contrast, SE: standard error, t.ratio: test statistic Download Figure 6-1, DOCX file.

10.1523/JNEUROSCI.0238-25.2025.f6-2 Figure 6-2 Interaction between category and E-field in left Anterior Insula as predictors of Reaction times. Mixed-effects regression model results of TMS E-field in left AIns and category as predictors of (log-transformed) reaction times, and planned comparisons to test for differences in the effects of E-field in left AIns between categories. Significant results are written in bold. Sum.Sq: Sum of squares, Mean.Sq: Sum of squares / degrees of freedom, NumDF, df: Degrees of freedom, DenDF: Denominator degrees of Freedom, estimate: estimated value of the contrast, SE: standard error, t.ratio: test statistics Download Figure 6-2, DOCX file.

10.1523/JNEUROSCI.0238-25.2025.f6-3 Figure 6-3 Interaction between category and E-field in right Anterior Insula as predictors of Reaction times. Mixed-effect regression model results of TMS E-field in right AIns and category as predictors of (log-transformed) reaction times, and planned comparisons to test for differences in the effects of E-field in right AIns between categories. Significant results are written in bold. Sum.Sq: Sum of squares, Mean.Sq: Sum of squares / degrees of freedom, NumDF, df: Degrees of freedom, DenDF: Denominator degrees of Freedom, estimate: estimated value of the contrast, SE: standard error, t.ratio: test statistic. Download Figure 6-3, DOCX file.

10.1523/JNEUROSCI.0238-25.2025.f6-4 Figure 6-4 Interaction between MAX E-field and category as predictors of Reaction Times MAX: maximum, E-field: electric field. **(A)** Adjusted predictions of the interaction between the electric field (E-field) induced in left MAX and category on RTs. Planned comparisons were non-significant (Emotion-Social: t = 0.420, p = 1, Emotion-Objects: t = 0.668, p = 1, Social-Objects: t = 0.246, p = 1). **(B)** Adjusted predictions of the interaction between the electric field (E-field) induced in right MAX and category on RTs. Planned comparisons were non-significant (Emotion-Social: t = -1.756, p = 0.209, Emotion-Objects: t = -1.815, p = 0.209, Social-Objects: t = -0.051, p = 0.959). **(A-B)** Error bars represent 95% confidence interval (CI) of the adjusted predictions. All p values were corrected for multiple comparisons using Holm correction. n.s. p > 0.05. Download Figure 6-4, TIF file.

10.1523/JNEUROSCI.0238-25.2025.f6-5 Figure 6-5 Interaction between PHC E-field and category as predictors of Reaction Times PHC: parahippocampal cortex, E-field: electric field. **(A)** Adjusted predictions of the interaction between the electric field (E-field) induced in left PHC and category on RTs. Planned comparisons were non-significant (Emotion-Social: t value = -0.241, p = 1, Emotion-Objects: t value = 0.258, p = 1, Social-Objects: t value: 0.508, p = 1). **(B)** Adjusted predictions of the interaction between the electric field (E-field) induced in right PHC and category on RTs. Planned comparisons were non-significant (Emotion-Social: t value = -1.193, p = 0.644, Emotion-Objects: t value = -1.241, p = 0.644, Social-Objects: t value: -0.048, p = 0.962). **(A-B)** Error bars represent 95% confidence interval (CI) of the adjusted predictions. All p values were corrected for multiple comparisons using Holm correction. n.s. p > 0.05. Download Figure 6-5, TIF file.

10.1523/JNEUROSCI.0238-25.2025.f6-6 Figure 6-6 Interaction between category and TMS site as predictors of Accuracy. Mixed-effect logistic regression model results of TMS site and category as predictors of accuracy, and planned comparisons between ipsilateral real and sham stimulations, for each semantic category. Significant results are written in bold. Chisq: Chi-squared statistic, Df: degrees of freedom, SE: standard error, z.ratio: test statistic Download Figure 6-6, DOCX file.

**Figure 7.**
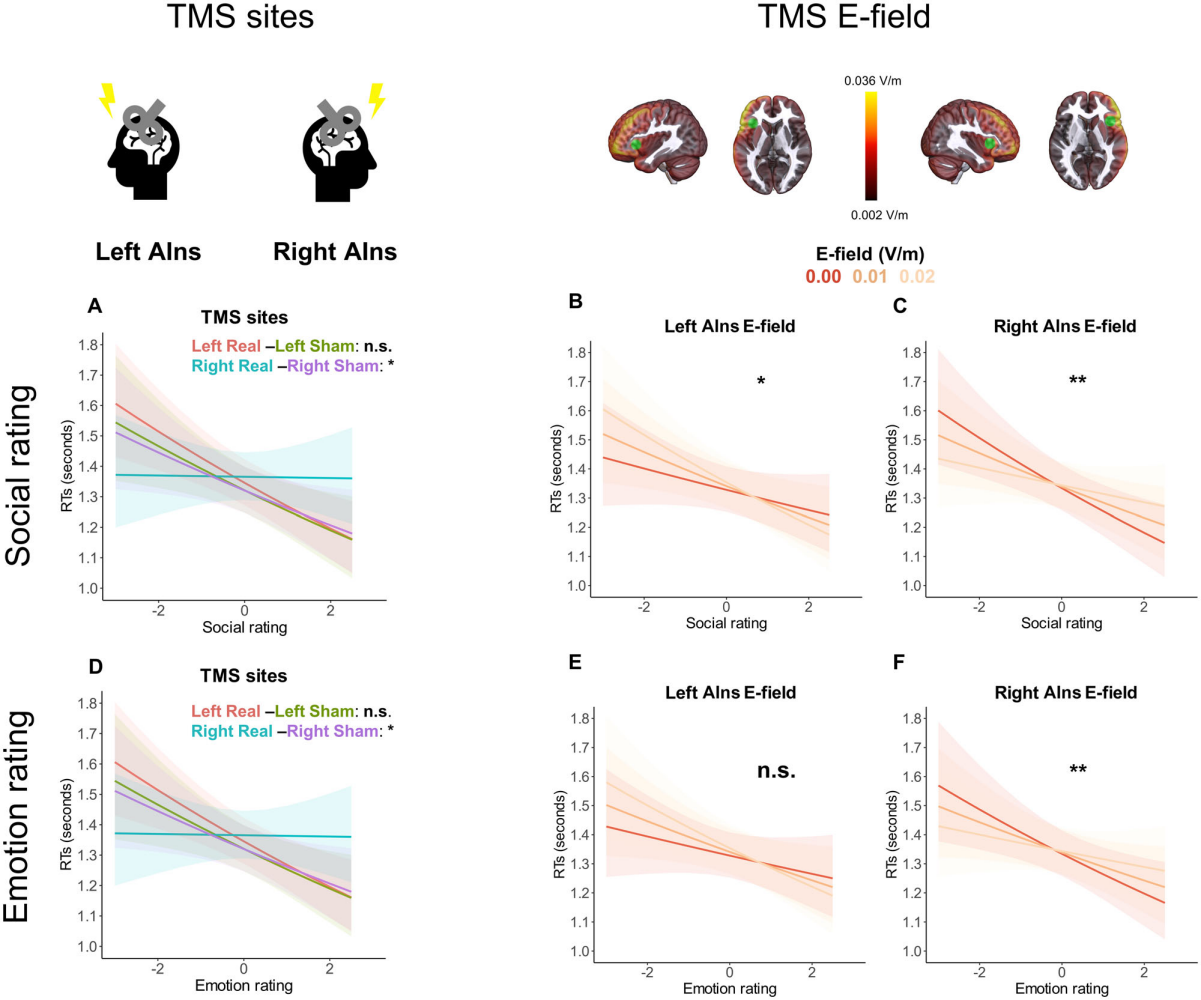
Semantic similarity task. Semantic ratings results with abstract triplets. RTs. AIns, anterior insula; E-field, electric field. Results are shown on the centered Social and Emotion rating. ***A***, ***D***, Adjusted predictions of reaction times (RTs) following each TMS condition, shown in the response scale. The comparisons between left real-left sham were not significant. In contrast, right real stimulation significantly hindered semantic processing, interfering with both the social (***A***) and the emotion (***D***) dimensions: subjects were no longer faster in responding to abstract triplets with higher social and emotion scores after right real compared with right sham stimulations. *p* values of the planned comparisons were corrected for multiple comparisons using Holm correction. ***B***, ***E***, Adjusted predictions of the interaction between the E-field induced in left AIns on Social (***B***) and Emotion (***E***) rating. The interaction with Social rating is significant and shows a negative trend. See ***A*** of Extended Data Figures 7-1 and 7-2 for the analysis with, respectively, left MAX E-field and left PHC E-field. ***C***, ***F***, Adjusted predictions of the interaction between the E-field induced in right AIns on Social (***C***) and Emotion (***F***) rating. Both interactions are significant and show a positive trend, whereby the facilitatory effect of the Emotion and Social rating is reduced in the presence of a higher E-field in the right AIns. See ***B*** of Extended Data Figures 7-1 and 7-2 for the analysis with, respectively, right MAX E-field and right PHC E-field. Error bars represent 95% confidence intervals (CI) of the adjusted predictions. **p < 0.01, *p < 0.05, n.s., *p* > 0.05. See Extended Data Figures 7-3–7-8 for the respective analysis with semantic ratings and E-field in AIns, MAX and PHC as predictors of accuracy for abstract triplets. See Extended Data Figures 7-9 to 7-16 for the analysis with semantic ratings and E-field in AIns as predictors of RTs and accuracy for concrete triplets.

10.1523/JNEUROSCI.0238-25.2025.f6-7 Figure 6-7 Interaction between category and E-field in right Anterior Insula as predictors of Accuracy. Mixed-effect logistic regression model results of TMS E-field in right AIns and category as predictors of accuracy, and planned comparisons to test for differences in the effects of E-field in right AIns between categories, showing the differences in the slope of the E-field effect on accuracy across categories. Significant results are written in bold. Chisq: Chi-squared statistic, Df: degrees of freedom, estimate: estimated value of the contrast, SE: standard error, z.ratio: test statistic Download Figure 6-7, DOCX file.

10.1523/JNEUROSCI.0238-25.2025.f6-8 Figure 6-8 Interaction between category and E-field in left Anterior Insula as predictors of Accuracy. Mixed-effect logistic regression model results of TMS E-field in left AIns and category as predictors of accuracy, and planned comparisons to test for differences in the effects of E-field in left AIns between categories, showing the differences in the slope of the E-field effect on accuracy across categories. Significant effects are written in bold. Chisq: Chi-squared statistic, Df: degrees of freedom, estimate: estimated value of the contrast, SE: standard error, z.ratio: test statistic Download Figure 6-8, DOCX file.

10.1523/JNEUROSCI.0238-25.2025.f6-9 Figure 6-9 Semantic similarity task. Category results. Accuracy AIns: Anterior Insula, E-field: electric field **(A-B)** Estimated marginal means of Accuracy (on the logit scale) following left (A) and right (B) TMS conditions. Planned comparisons were non-significant. **(C**) Adjusted predictions of the interaction between the electric field (E-field) induced in left Anterior Insula (AIns) and category on Accuracy. Planned comparisons were non-significant. **(D)** Adjusted predictions of the interaction between the electric field (E-field) induced in right Anterior Insula (AIns) and category on Accuracy. Planned comparisons were non-significant. **(A-B)** Error bars represent standard errors of the marginal means. (C-D) Error bars represent 95% confidence interval (CI) of the adjusted predictions. All p values were corrected for multiple comparisons using Holm correction. n.s. p > 0.05. Download Figure 6-9, TIF file.

10.1523/JNEUROSCI.0238-25.2025.f6-10 Figure 6-10 Interaction between MAX E-field and category as predictors of Accuracy MAX: maximum, E-field: electric field. **(A)** Adjusted predictions of the interaction between the electric field (E-field) induced in left MAX and category on Accuracy. Planned comparisons were non-significant (Emotion-Social: z value = 0.965, p = 1, Emotion-Objects: z value = 0.426, p = 1, Social-Objects: z value: -0.382, p = 1). **(B)** Adjusted predictions of the interaction between the electric field (E-field) induced in right MAX and category on Accuracy. Planned comparisons were non-significant (Emotion-Social: z value = -1.588, p = 0.337, Emotion-Objects: z value = -0.545, p = 0.864, Social-Objects: z value: 0.786, p = 0.864). **(A-B)** Error bars represent standard errors of the marginal means. (C-D) Error bars represent 95% confidence interval (CI) of the adjusted predictions. All p values were corrected for multiple comparisons using Holm correction. n.s. p > 0.05. Download Figure 6-10, TIF file.

10.1523/JNEUROSCI.0238-25.2025.f6-11 Figure 6-11 Interaction between PHC E-field and category as predictors of Accuracy PHC: parahippocampal cortex, E-field: electric field. **(A)** Adjusted predictions of the interaction between the electric field (E-field) induced in left PHC and category on Accuracy. Planned comparisons were non-significant (Emotion-Social: z value = -0.629, p = 1, Emotion-Objects: z value = -1.034, p = 0.903, Social-Objects: z value: -0.492, p = 1). **(B)** Adjusted predictions of the interaction between the electric field (E-field) induced in right PHC and category on Accuracy. Planned comparisons were non-significant (Emotion-Social: z value = -0.638, p = 1, Emotion-Objects: z value = 0.086, p = 1, Social-Objects: z value: 0.616, p = 1). Download Figure 6-11, TIF file.

E-field analyses with right AIns also revealed a significant effect of category (*F* = 9.865, *p* < 0.001), while the effect of MagnE was not significant. Again, we found a significant effect of covariate semantic similarity similar (*F* = 9.797, *p* = 0.002) and triplet length (*F* = 5.642, *p* = 0.019). The interaction between E-field in right AIns and category was not significant. Planned comparisons were not significant (all *p* > 0.05; Fig. 6*D*; Extended Data Fig. 6-3).

On the contrary, in the E-field analysis on the left AIns, the effect of MagnE was significant (magnE Left AIns: *F* = 7.352, *p* = 0.007), as was the effect of category (*F* = 9.858, *p* < 0.001), and the effects of semantic similarity similar (*F* = 9.649, *p* = 0.002) and triplet length (*F* = 5.649, *p* = 0.019). The interaction between category and MagnE left AIns was not significant. Planned comparisons were nonsignificant (all *p* > 0.05; Fig. 6*C*; Extended Data Fig. 6-2).

Likewise, E-field analyses with right MAX and with left MAX did not present any significant comparisons (all *p* > 0.05; Extended Data Fig. 6-4). E-field analysis with right PHC and left PHC E-field did not result in any significant comparisons either (all *p* > 0.05; Extended Data Fig. 6-5).

The analysis of semantic accuracy did not reveal any significant interaction between category and TMS site or E-field in any ROI either (Extended Data Figs. 6-6 to 6-11).

In brief, neither TMS nor E-field in either left or right AIns interacted with category. E-field induced in left AIns had a nonspecific effect, whereby the higher the E-field, the slower the RT.

##### Semantic ratings

Abstract triplets. According to the dimensional approach, we also tested the interaction between TMS and semantic ratings on the Emotion and Social dimension of abstract triplets. The main effect of TMS site (*F* = 5,313, *p* = 0.001), Social rating (*F* = 5,212, *p* = 0.024), TMS and Social rating interaction (*F* = 4.304, *p* = 0.005), and TMS and Emotion rating interaction (*F* = 3.678, *p* = 0.012) were all significant. Only the main effect of Emotion rating did not reach significance (*F* = −1.92, *p* = 0.055). Both Social and Emotion showed a similar, negative trend: the higher the triplet rating on Social and/or Emotion scale, the faster the response. Semantic similarity similar (*F* = 5.877, *p* = 0.017) and triplet length (*F* = 12.762, *p* = 0.001) also showed significant effects, whereas semantic similarity distant did not.

Planned comparisons tested whether the slope of Emotion and Social rating differed depending on TMS site. We found a significant difference in the slopes of Social rating effect between Right Insula real-Right Insula sham stimulation (*T* = 2,420, *p* = 0.031) and in the slope of Emotion rating effect between Right Insula real-Right Insula sham stimulation (*T* = 2.306, *p* = 0.042; Fig. 7*A–D*). In both cases, the real Right Insula stimulation slowed responses to triplets with higher Social and Emotion ratings. The interaction with Left Insula stimulation was not significant in either case (all *p* > 0.05; Fig. 7*A–D*, Table 3).

10.1523/JNEUROSCI.0238-25.2025.f7-1 Figure 7-1 Interaction between MAX E-field and semantic ratings as predictors of Reaction Times of Abstract triplets MAX: maximum, E-field: electric field. Results are shown on the centred Social and Emotion rating. **(A, C)** Adjusted predictions of the interaction between the left MAX E-field on Emotion (A) and Social (C) rating. The interactions are not significant (left MAX E-field*Emotion rating: F = 2.551, p = 0.110, left MAX E-field*Social rating: F = 2.746, p = 0.098). **(B, D)** Adjusted predictions of the interaction between the right MAX E-field on Emotion (B) and Social (D) rating. Both interactions are significant and show a positive trend, whereby the facilitatory effect of the Emotion and Social rating is reduced in the presence of a higher E-field in the right MAX (right MAX E-field*Emotion rating: F = 5.168, p = 0.023, right MAX E-field*Social rating: F = 7.913, p = 0.005). Error bars represent 95% confidence intervals (CI) of the adjusted predictions. ** p < 0.01, * p < 0.05, n.s. p > 0.05. Download Figure 7-1, TIF file.

10.1523/JNEUROSCI.0238-25.2025.f7-2 Figure 7-2 Interaction between PHC E-field and semantic ratings as predictors of Reaction Times of abstract triplets PHC: parahippocampal cortex, E-field: electric field. Results are shown on the centred Social and Emotion rating. **(A, C)** Adjusted predictions of the interaction between the left PHC E-field on Emotion (A) and Social (C) rating. The interactions are not significant (left PHC E-field*Emotion rating: F value = 1.146, p = 0.284, left PHC*Social rating: F value = 1.163, p = 0.281). **(B, D)** Adjusted predictions of the interaction between the right PHC E-field on Emotion (B) and Social (D) rating. Interactions are not significant (right PHC E-field*Emotion rating: F value = 0.281, p = 0.596, right PHC*Social rating: F value = 0.844, p = 0.358). Error bars represent 95% confidence intervals (CI) of the adjusted predictions. n.s. p > 0.05. Download Figure 7-2, TIF file.

10.1523/JNEUROSCI.0238-25.2025.f7-3 Figure 7-3 Interaction between semantic ratings and TMS site as predictors of Accuracy of Abstract triplets. Mixed-effect logistic regression model results of TMS site and semantic ratings as predictors of accuracy, and planned comparisons between ipsilateral real and sham stimulations, showing the difference in the average slope of emotion and social scores effect between right real and right sham TMS conditions, and between left real and left sham TMS conditions. Significant results are written in bold. Chisq: Chi-squared statistic, Df: degrees of freedom, estimate: estimated value of the contrast, SE: standard error, z.ratio: test statistic. Download Figure 7-3, DOCX file.

10.1523/JNEUROSCI.0238-25.2025.f7-4 Figure 7-4 Interaction between semantic ratings and E-field in right Anterior Insula as predictors of Accuracy of Abstract triplets. Mixed-effects logistic regression model results of TMS E-field in right AIns and semantic ratings as predictors of accuracy, where the last two rows represent the interaction between the magnitude of the E-field inside right AIns and respectively emotion and social rating. Significant effects are written in bold. Chisq: Chi-squared statistic, Df: degrees of freedom. Download Figure 7-4, DOCX file.

10.1523/JNEUROSCI.0238-25.2025.f7-5 Figure 7-5 Interaction between semantic ratings and E-field in left Anterior Insula as predictors of Accuracy of Abstract triplets. Mixed-effects logistic regression model results of TMS E-field in left AIns and semantic ratings as predictors of accuracy, where the last two rows represent the interaction between the magnitude of the E-field inside left AIns and respectively emotion and social rating. Significant effects are written in bold. Chisq: Chi-squared statistic, Df: degrees of freedom. Download Figure 7-5, DOCX file.

10.1523/JNEUROSCI.0238-25.2025.f7-6 Figure 7-6 Semantic similarity task. Semantic ratings results with abstract triplets. Accuracy AIns: Anterior Insula, E-field: electric field. Results are shown on the centred Social and Emotion rating. **(A, D)** Adjusted predictions of Accuracy following each TMS condition, transformed from logit to probability scale. The comparisons between left real-left sham and between right real-right sham were not significant. P values of the planned comparisons were corrected for multiple comparisons using Holm correction. **(B, E)** Adjusted predictions of the interaction between the E-field induced in left AIns with Emotion (B) and Social (E) rating on Accuracy. The interactions are not significant. **(C, F)** Adjusted predictions of the interaction between the E-field induced in right AIns with Emotion (B) and Social (E) rating on Accuracy. Both interactions are significant and show a negative trend, whereby the higher E-field in the right AIns determines the lower probability of responding correctly to triplets the higher their emotion and social rating. Error bars represent 95% confidence intervals (CI) of the adjusted predictions. * p < 0.05, n.s. p > 0.05. Download Figure 7-6, TIF file.

10.1523/JNEUROSCI.0238-25.2025.f7-7 Figure 7-7 Interaction between MAX E-field and semantic ratings as predictors of Accuracy of abstract triplets MAX: maximum, E-field: electric field. Results are shown on the centred Social and Emotion rating. **(A, C)** Adjusted predictions of the interaction between the E-field induced in left MAX with Emotion (A) and Social (C) rating on Accuracy. The interactions are not significant (left MAX*Emotion rating: Χ^2^ = 0.054, p = 0.815, left MAX* Social rating: Χ^2^ = 0.008, p = 0.927). **(B, D)** Adjusted predictions of the interaction between the E-field induced in right MAX with Emotion (B) and Social (D) rating on Accuracy. The interaction with Emotion rating is significant and shows a negative trend, whereby the higher E-field in the right MAX determines the lower probability of responding correctly to triplets the higher their emotion rating (Χ^2^ = 4.850, p = 0.028). The interaction with Social rating is not significant (Χ^2^ = 3.084, p = 0.079). Error bars represent 95% confidence intervals (CI) of the adjusted predictions. ** p < 0.01, * p < 0.05, n.s. p > 0.05. Download Figure 7-7, TIF file.

10.1523/JNEUROSCI.0238-25.2025.f7-8 Figure 7-8 Interaction between PHC E-field and semantic ratings as predictors of Accuracy of abstract triplets PHC: parahippocampal cortex, E-field: electric field. Results are shown on the centred Social and Emotion rating. **(A, C)** Adjusted predictions of the interaction between the E-field induced in left PHC with Emotion (A) and Social (C) rating on Accuracy. The interactions are not significant (left PHC E-field*Emotion rating: Χ^2^ = 2.179, p = 0.140, left PHC E-field*Social rating: Χ^2^ = 1.431, p = 0.232). **(B, D)** Adjusted predictions of the interaction between the E-field induced in right PHC with Emotion (B) and Social (D) rating on Accuracy. The interactions are not significant (right PHC E-field*Emotion rating: Χ^2^ = 1.806, p = 0.179, right PHC E-field*Social rating: Χ^2^ = 1.336, p = 0.248). Error bars represent 95% confidence intervals (CI) of the adjusted predictions. n.s. p > 0.05. Download Figure 7-8, TIF file.

10.1523/JNEUROSCI.0238-25.2025.f7-9 Figure 7-9 Interaction between TMS site and semantic ratings as predictors of Reaction times of Concrete triplets. Mixed-effect regression model results of TMS site and semantic ratings as predictors of (log-transformed) reaction times to concrete triplets, and planned comparisons between ipsilateral real and sham stimulations, showing the difference in the average slope of emotion and social scores effect between right real and right sham TMS conditions, and between left real and left sham TMS conditions. Significant results are written in bold. Sum.Sq: Sum of squares, Mean.Sq: Sum of squares / degrees of freedom, NumDF, df: Degrees of freedom, DenDF: Denominator degrees of Freedom, estimate: estimated value of the contrast, SE: standard error, t.ratio: test statistics. Download Figure 7-9, TIF file.

10.1523/JNEUROSCI.0238-25.2025.f7-10 Figure 7-10 Interaction between semantic ratings and E-field in right Anterior Insula as predictors of Reaction times of Concrete triplets. Mixed-effect regression model results of TMS E-field in right AIns and semantic ratings as predictors of (log-transformed) reaction times to concrete triplets, where the last two rows represent the interaction between the magnitude of the E-field inside right AIns and respectively emotion and social rating. Significant effects are written in bold. Sum.Sq: Sum of squares, Mean.Sq: Sum of squares / degrees of freedom, NumDF: Degrees of freedom, DenDF: Denominator degrees of Freedom. Download Figure 7-10, DOCX file.

10.1523/JNEUROSCI.0238-25.2025.f7-11 Figure 7-11 Interaction between semantic ratings and E-field in left Anterior Insula as predictors of Reaction times of Concrete triplets. Mixed-effect regression model results of TMS E-field in left AIns and semantic ratings as predictors of (log-transformed) reaction times to concrete triplets, where the last two rows represent the interaction between the magnitude of the E-field inside left AIns and respectively emotion and social rating. Significant effects are written in bold. Sum.Sq: Sum of squares, Mean.Sq: Sum of squares / degrees of freedom, NumDF: Degrees of freedom, DenDF: Denominator degrees of Freedom. Download Figure 7-11, DOCX file.

10.1523/JNEUROSCI.0238-25.2025.f7-12 Figure 7-12 Semantic similarity task. Semantic ratings results with concrete triplets. RTs AIns: Anterior Insula, E-field: electric field. Results are shown on the centred Social and Emotion rating. **(A, D)** Adjusted predictions of reaction times (RTs) following each TMS condition, shown in the response scale. The comparisons between left real-left sham and between right real-right sham were not significant, meaning the TMS condition did not significantly change the effect of social or emotion rating on the RTs for concrete triplets. P values of the planned comparisons were corrected for multiple comparisons using Holm correction. **(B, E)** Adjusted predictions of the interaction between the E-field induced in left AIns on Social (B) and Emotion (E) rating. The interactions are not significant, meaning the magnitude of the E-field inside left AIns did not significantly change the effect of social or emotion rating on the RTs for concrete triplets. **(C, F)** Adjusted predictions of the interaction between the E-field induced in right AIns with Emotion (B) and Social (E) rating. Interactions are not significant, meaning the magnitude of the E-field inside right AIns did not significantly change the effect of social or emotion rating on the RTs for concrete triplets. Error bars represent 95% confidence intervals (CI) of the adjusted predictions. ** p < 0.01, * p < 0.05, n.s. p > 0.05. Download Figure 7-12, TIF file.

10.1523/JNEUROSCI.0238-25.2025.f7-13 Figure 7-13 Interaction between TMS site and semantic ratings as predictors of Accuracy of Concrete triplets. Mixed-effects logistic regression model results of TMS site and semantic ratings as predictors of accuracy to concrete triplets, and planned comparisons between ipsilateral real and sham stimulations, showing the difference in the average slope of emotion and social scores effect between right real and right sham TMS conditions, and between left real and left sham TMS conditions. Significant results are written in bold. Chisq: Chi-squared statistic, Df: degrees of freedom, estimate: estimated value of the contrast, SE: standard error, z.ratio: test statistic. Download Figure 7-13, DOCX file.

10.1523/JNEUROSCI.0238-25.2025.f7-14 Figure 7-14 Interaction between semantic ratings and E-field in right Anterior Insula as predictors of Accuracy of Concrete triplets. Mixed-effects logistic regression model results of TMS E-field in right AIns and semantic ratings as predictors of accuracy to concrete triplets, where the last two rows represent the interaction between the magnitude of the E-field inside right AIns and respectively emotion and social rating. Significant effects are written in bold. Chisq: Chi-squared statistic, Df: degrees of freedom. Download Figure 7-14, DOCX file.

10.1523/JNEUROSCI.0238-25.2025.f7-15 Figure 7-15 Interaction between semantic ratings and E-field in left Anterior Insula as predictors of Accuracy of Concrete triplets. Mixed-effects logistic regression model results of TMS E-field in left AIns and semantic ratings as predictors of accuracy to concrete triplets, where the last two rows represent the interaction between the magnitude of the E-field inside left AIns and respectively emotion and social rating. Significant effects are written in bold. Chisq: Chi-squared statistic, Df: degrees of freedom. Download Figure 7-15, DOCX file.

10.1523/JNEUROSCI.0238-25.2025.f7-16 Figure 7-16 Semantic similarity task. Semantic ratings results with concrete triplets. Accuracy AIns: Anterior Insula, E-field: electric field. Results are shown on the centred Social and Emotion rating. **(A, D)** Adjusted predictions of Accuracy following each TMS condition, transformed from logit to probability scale. The comparisons between left real-left sham and between right real-right sham were not significant, meaning the TMS condition did not significantly change the effect of social or emotion rating on the accuracy for concrete triplets. P values of the planned comparisons were corrected for multiple comparisons using Holm correction. **(B, E)** Adjusted predictions of the interaction between the E-field induced in left AIns with Emotion (B) and Social (E) rating on Accuracy. The interactions are not significant, meaning the magnitude of the E-field inside left AIns did not significantly change the effect of social or emotion rating on the accuracy for concrete triplets **(C, F)** Adjusted predictions of the interaction between the E-field induced in right AIns with Emotion (B) and Social (E) rating on Accuracy. Interactions are not significant, meaning the magnitude of the E-field inside right AIns did not significantly change the effect of social or emotion rating on the accuracy for concrete triplets. Error bars represent 95% confidence intervals (CI) of the adjusted predictions. n.s. p > 0.05. Download Figure 7-16, TIF file.

**Table 3.** Interaction between semantic ratings and TMS site as predictors of reaction times of abstract triplets

	Sum.Sq	Mean.Sq	NumDF	DenDF	*F*.value	*p* value
Model results						
Emotion rating	0.217	0.217	1	103.339	3.676	0.058
Social rating	**0.308**	**0.308**	**1**	**103.423**	**5.212**	**0.024**
TMS site	**0.941**	**0.314**	**3**	**4,707.745**	**5.313**	**0.001**
Semantic similarity similars	**0.347**	**0.347**	**1**	**103.588**	**5.877**	**0.017**
Semantic similarity distants	0.003	0.003	1	104.638	0.048	0.827
Triplet length	**0.754**	**0.754**	**1**	**103.084**	**12.762**	**0.001**
Emotion rating:TMS site	**0.651**	**0.217**	**3**	**4,707.231**	**3.678**	**0.012**
Social rating:TMS site	**0.762**	**0.254**	**3**	**4,707.033**	**4.304**	**0.005**
Contrast	Estimate	SE	df	*t*.ratio	*p*.value	
Planned comparisons						
Emotion rating and TMS site						
Left Real-Left Sham	−0.009	0.019	4,727.923	−0.448	0.654	
Right Real-Right Sham	**0.043**	**0.019**	**4,715.815**	**2.306**	**0.042**	
Social rating and TMS site						
Left Real-Left Sham	−0.007	0.018	4,726.225	−0.376	0.707	
Right Real-Right Sham	**0.044**	**0.018**	**4,716.928**	**2.420**	**0.031**	

Mixed-effects regression model results of TMS site and semantic ratings predicting (log-transformed) reaction times, and planned comparisons between ipsilateral real and sham stimulations, showing the difference in the average slope of emotion and social scores effect between right real and right sham TMS conditions and between left real and left sham TMS conditions. In the right real compared with the right sham condition, the stimulation significantly interacted with both social and emotion scores, interfering with their facilitatory effect on RTs. Significant results are written in bold. Sum.Sq, sum of squares; Mean.Sq, sum of squares/degrees of freedom; NumDF, degrees of freedom; DenDF, denominator degrees of freedom; estimate, estimated value of the contrast; SE, standard error; *t*.ratio, test statistics.

10.1523/JNEUROSCI.0238-25.2025.t3-1 Table 3-1 Single-subject MNI MAX PEAKS MNI coordinates of E-field maximum peak (MAX E-field) during left and right TMS, for each subject. Download Table 3-1, DOCX file.

In the E-field analysis with right AIns, the main effects of Social rating (*F* = 5.473, *p* = 0.021), MagnE Right AIns and Social rating interaction (*F* = 10.028, *p* = 0.002), and MagnE Right AIns and Emotion rating interaction (*F* = 6.828, *p* = 0.009) were significant. The main effect of Emotion rating and MagnE Right AIns did not reach significance. Semantic similarity similar (*F* = 5.589, *p* = 0.020) and triplet length (*F* = 12.924, *p* ≤ 0.001) also showed significant effects, whereas semantic similarity distant did not. The interaction between MagnE right AIns and Social and Emotion ratings revealed a positive trend, whereby higher MagnE values in Right Insula determined slower RTs to triplets with higher Social and Emotion ratings (Fig. 7*C–F*; Table 4).

**Table 4.** Interaction between semantic ratings and E-field in right anterior insula as predictors of reaction times of abstract triplets

Model results	Sum.Sq	Mean.Sq	NumDF	DenDF	*F*.value	*p* value
Right AIns E-field	0.057	0.057	1	4,518.688	0.962	0.327
Emotion_rating	0.229	0.229	1	103.432	3.874	0.052
Social_rating	**0.323**	**0.323**	**1**	**103.524**	**5.473**	**0.021**
Semantic similarity similars	**0.330**	**0.330**	**1**	**103.614**	**5.589**	**0.020**
Semantic similarity distants	0.000	0.000	1	104.846	0.001	0.970
Triplet length	**0.764**	**0.764**	**1**	**103.150**	**12.924**	**0.000**
Right AIns E-field:Emotion_rating	**0.404**	**0.404**	**1**	**4,512.546**	**6.828**	**0.009**
Right AIns E-field:Social_rating	**0.593**	**0.593**	**1**	**4,512.586**	**10.028**	**0.002**

Mixed-effect regression model results of TMS E-field in right AIns and semantic ratings predicting (log-transformed) reaction times to abstract triplets, where the last two rows represent the interaction between the magnitude of the E-field inside right AIns and respectively emotion and social rating. Significant effects are written in bold. See Extended Data Table 4-1 for the respective analysis with left AIns E-field and semantic ratings as predictors of RTs. Sum.Sq, sum of squares; Mean.Sq, sum of squares/degrees of freedom; NumDF, degrees of freedom; DenDF, denominator degrees of freedom.

10.1523/JNEUROSCI.0238-25.2025.t4-1 Table 4-1 Interaction between semantic ratings and E-field in left Anterior Insula as predictors of Reaction times of Abstract triplets. Mixed-effects regression model results of TMS E-field in left AIns and semantic ratings as predictors of (log-transformed) reaction times to abstract triplets, where the last two rows represent the interaction between the magnitude of the E-field inside left AIns and respectively emotion and social rating. Significant effects are written in bold. Sum.Sq: Sum of squares, Mean.Sq: Sum of squares / degrees of freedom, NumDF: Degrees of freedom, DenDF: Denominator degrees of Freedom Download Table 4-1, DOCX file.

In the E-field analysis with left AIns, the main effect of MagnE was significant (*F* = 5.602, *p* = 0.018), as Social rating (*F* = 5.281, *p* = 0.024), and the interaction between Social rating and E-field (*F* = 4.450, 0.035), whereas all other effects were not significant (Fig. 7*B–E*; Extended Data Table 4-1). Semantic similarity similar (*F* = 5.427, *p* = 0.022) and triplet length (*F* = 12.989, *p* < 0.001) also showed significant effects, whereas semantic similarity distant did not.

In the E-field analysis with right MAX, the interaction between MagnE right MAX and Social and Emotion ratings were also significant and revealed a positive trend (right MAX E-field × Emotion rating: *F* = 5.168, *p* = 0.023, right MAX E-field × Social rating: *F* = 7.913, *p* = 0.005), whereby higher MagnE values in Right MAX determined slower RTs to triplets with higher Social and Emotion ratings (Extended Data Fig. 7-1*B*,*D*). In the E-field analysis with left MAX, the two interactions were not significant (left MAX E-field × Emotion rating: *F* = 2.551, *p* = 0.110, left MAX E-field × Social rating: *F* = 2.746, *p* = 0.098; Extended Data Fig. 7-1*A*,*C*).

In the E-field analysis with right PHC, the interaction between the E-field in right PHC and social or emotion ratings is not significant (right PHC × Emotion rating: *F* value = 0.281, *p* = 0.596, right PHC × Social rating: *F* value = 0.844, *p* = 0.358). In the E-field analysis with left PHC, the interactions with left PHC are not significant either (left PHC × Emotion rating: *F* value = 1.146, *p* = 0.284, left PHC × Social rating: *F* value = 1.163, *p* = 0.281; Extended Data Fig. 7-2).

Since both right AIns and right MAX models presented significant interactions with Emotion and Social semantic ratings, we compared the fit of the models.

Right MAX ROI model loglik and AIC were, respectively, −170.145 and 364.289, and those of right AIns model were −169.950 and 363.899. The results indicate that right AIns model is a better fit (higher loglik, smaller AIC) than right MAX in explaining semantic RTs data.

To summarize, both TMS and E-field analysis revealed a significant interaction between right AIns and social and emotion scale, whereby triplets with higher Social and Emotion ratings were particularly affected by stimulation. We also found a significant interaction between E-field in the left insula and the social scale, whereby triplets with higher Social ratings were facilitated by higher E-field in left AIns.

E-field analysis of accuracy revealed a consistent pattern, namely, the higher the E-field induced in right AIns, the lower the probability of responding correctly to triplets with higher Social and Emotion ratings (Extended Data Figs. 7-3 to 7-6). In other words, the higher E-field in right AIns interfered with participants’ ability to make correct semantic decisions on highly Emotional and Social triplets.

E-field in right PHC did not interact with semantic ratings in predicting semantic accuracy (right PHC  × Emotion rating: *Χ*^2^ = 1.806, *p* = 0.179, right PHC × Social rating: *Χ*^2^ = 1.336, *p* = 0.248; Extended Data Fig. 7-8). E-field in left PHC did not interact either (left PHC × Emotion rating: *Χ*^2^ = 2.179, *p* = 0.140, left PHC × Social rating: *Χ*^2^ = 1.431, *p* = 0.232; Extended Data Fig. 7-8). Left MAX E-field did not interact with semantic ratings in predicting accuracy either (left MAX × Emotion rating: *Χ*^2^ = 0.054, *p* = 0.815, left MAX × Social rating: *Χ*^2^ = 0.008, *p* = 0.927; Extended Data Fig. 7-7). The right MAX E-field significantly interacted with Emotion rating only (*Χ*^2^ = 4.850, *p* = 0.028), whereas the interaction with social rating was not significant (*Χ*^2^ = 3.084, *p* = 0.079; Extended Data Fig. 7-7). We compared the right AIns and right MAX models, and similarly to the models predicting semantic RTs, right AIns model provided a better fit to the semantic accuracy data compared with the right MAX model (loglik and AIC were, respectively, −1,259.762 and 2,541.523 for right AINs and −1,260.101 and 2,542.202 for right MAX model).

Concrete triplets. As a control analysis, we tested the interaction between TMS and Emotion and Social ratings on concrete triplets. If our results are specific to the abstract domain, we should not find significant results with this latter analysis.

First, in the TMS site analysis, Emotion ratings and Social ratings did not exert a main effect on RTs to concrete triplets (Emotion rating: *F* value = 0.898, *p* = 0.347, Social rating: *F* value = 0.113, *p* = 0.738). The planned comparisons testing whether TMS site interacted with the semantic ratings also did not show any significant results, either for Emotion rating (Left Real-Left Sham: *t* value = 1.009, *p* = 0.500, Right-Real-Right Sham: *t* value = 1.151, *p* = 0.500) or for Social rating (Left Real-Left Sham: *t* value = −1.161, *p* = 0.492, Right-Real-Right Sham: *t* value = −1.009, *p* = 0.492; Extended Data Figs. 7-9, 7-12).

In the right anterior insula (AIns) E-field analysis, neither Social nor Emotion ratings showed a main effect on RTs (Emotion rating: *F* value = 0.985, *p* = 0.325, Social rating: *F* value = 0.068, *p* = 0.795). The interactions with right AIns are not significant either (right AIns E-field × Emotion rating: *F* value = 0.021, *p* = 0.884, right AIns E-field × Social rating: *F* value = 1.987, *p* = 0.159; Extended Data Figs. 7-10, 7-12).

In the left AIns E-field analysis, neither Social nor Emotion ratings showed a main effect on RTs either (Emotion rating: *F* value = 1.000, *p* = 0.321, Social rating: *F* value = 0.070, *p* = 0.792). The interactions with left AIns are not significant either (left AIns E-field × Emotion rating: *F* value = 1.878, *p* = 0.171, left AIns E-field × Social rating: *F* value = 0.010, *p* = 0.921; Extended Data Figs. 7-11, 7-12).

The analysis of semantic accuracy revealed the same pattern of results: emotion and social ratings did not exert a significant effect of accuracy, and the interactions with TMS site and with right and left AIns E-field are all nonsignificant either (Extended Data Figs. 7-13–7-16).

Overall, these results confirm that social and emotion value of concrete triplets did not significantly interfere with semantic judgments, while in abstract triplets, social score significantly facilitated semantic judgments. Moreover, we confirmed, both with the TMS site and the TMS E-field analyses, that the stimulation targeting right and left AIns did not interact with concrete triplets’ social and emotional dimensions. Given the high number of observations (*N* = 2,759), we believe this lack of interactions to reflect a true null result, which strengthens the conclusion that the right AIns is involved in processing the emotion of social dimensions of abstract concepts specifically.

## Discussion

TMS on the right AIns significantly interfered with interoceptive accuracy. In the semantic task, the effect was also right lateralized; it was observed in both the emotion and the social dimensions and emerged only using a dimensional approach and only with abstract but not with concrete triplets.

### Insula in interoception

In the heartbeat counting task, rTMS over the right AIns interfered with interoceptive processing (Fig. 5*A*,*B*). This finding aligns with previous neuroimaging studies, showing that people's ability to detect their heartbeats correlates with activity or gray matter volume in right AIns (Critchley et al., 2004; Pollatos et al., 2007b). rTMS studies confirmed this, finding a decrease in interoceptive accuracy after right AIns inhibition (Pollatos et al., 2016; Mai et al., 2019), although in these studies, the left AIns was not stimulated, hence the effect of its inhibition was not tested.

However, concerns have been expressed regarding the plausibility of reaching AIns through noninvasive brain stimulation (NIBS; Coll et al., 2017). To ensure that our results could be ascribed to the rTMS interference on AIns, we simulated the TMS-induced E-field in left and right AIns, and used it to predict interoceptive accuracy.

If the E-field in the AIns ROI were found to exert a significant effect on interoceptive accuracy, it would demonstrate that AIns had a direct role in the observed response. The results of the E-field models confirmed our findings. While E-field in left AIns exerted no effect (Fig. 5*C*), E-field in right AIns interfered with participants’ performance, with higher E-field predicting lower interoceptive accuracy (Fig. 5*D*).

Together, these analyses demonstrate the effectiveness of stimulation and align with Craig’s theory on the role of right AIns in interoceptive awareness (Craig, 2009).

### Insula in emotion

In the semantic similarity task, right AIns stimulation interfered with judgments on abstract triplets with higher scores on the Emotion dimension (Fig. 7*D*).

AIns has a role in emotion experience (Duerden et al., 2013). Its activity supports emotion recognition of facial expressions (Papagno et al., 2016; Motomura et al., 2019) and perceived (un)pleasantness of visual stimuli (Mai et al., 2019; Feng et al., 2021).

Crucially, AIns is involved in the processing of emotional words. While some of these studies employed emotion-referring words (i.e., words referring to internal states, i.e., “fear”; Wilson-Mendenhall et al., 2011; Moseley et al., 2012; Lebois et al., 2020), others used “emotional words,” where emotionality is defined in terms of high valence and/or arousal (Citron et al., 2014; Vigliocco et al., 2014). Emotional words refer not only to emotions but also to other types of abstract concepts, such as social ones (e.g., “war,” “traitor”). In our stimuli, words belonging to the Emotion category were emotion-referring words, but ratings on the Emotion dimension were obtained for all stimuli.

The results show that right AIns stimulation interacted with Emotion dimension (Fig. 7*D*): after right real stimulation, compared with sham, the higher emotional connotation no longer facilitated decisions on the abstract triplets. Consistently, the higher the E-field induced in right AIns, the lower the facilitation for abstract triplets with higher emotional scores (i.e., slower RTs and lower accuracy; Fig. 7*F*; Extended Data Fig. 7-6*F*).

We did not find any interaction between AIns stimulation and Emotion category (Fig. 6). Abstract concepts might be better characterized by a dimensional rather than a categorical approach, because boundaries between categories of abstract concepts are difficult to define (Desai et al., 2018; Harpaintner et al., 2018).

As confirmed by our control analysis, these effects were not present with concrete object triplets, supporting the idea that right AIns is predominantly involved with the emotional content of abstract concepts.

These findings align with an embodied view of the abstract domain, where the emotional content of abstract words is supported by the right AIns, an area engaged in the corresponding nonlinguistic emotional experience (Parrinello et al., 2022).

The right AIns role in emotion experience has also been linked with its role in interoception (Zaki et al., 2012; Nguyen et al., 2016; Adolfi et al., 2017). Intriguingly, interoception is an important modality for the perceptual grounding of abstract concepts (Connell et al., 2018; Repetto et al., 2023) and processing of abstract emotion words and heartbeat interact with each other (Vergallito et al., 2019; Villani et al., 2021). Based on that, we hypothesize that in right AIns, interoceptive feedback may inform semantic judgments by providing a grounded representation of emotional linguistic content.

### Insula in social experience

In the semantic similarity task, right AIns stimulation interfered with abstract triplets with higher scores on the Social dimension (Fig. 7*A*).

The right AIns has been related to social processes, such as learning of social structure (Lau et al., 2020), social prediction errors (Henco et al., 2020), empathy (Corradi-Dell’Acqua et al., 2016), social connotation of speech and action (Di Cesare et al., 2018), and socially induced emotions (Immordino-Yang et al., 2014).

Social concepts, or social-referring concepts, refer to interpersonal relationships (Troche et al., 2014), to values or ideologies (Diveica et al., 2023). Even though AIns does not appear among the areas involved in the representation of social concepts (Arioli et al., 2021), studies that investigated the representation of emotional concepts finding activation in AIns (Citron et al., 2014; Vigliocco et al., 2014) included social-referring words as stimuli (e.g., “war,” “seduction”). On the other hand, emotion-referring words (such as “jealousy”) refer to internal states that are experienced in interpersonal situations. In our stimuli, words belonging to the social category were social-referring words, while ratings on the social dimension were obtained for all stimuli.

We observed a main effect of social dimension, i.e., a negative estimate of the effect of social rating (also observed as a nonsignificant trend for emotion rating), reflecting participants’ tendency to respond faster to triplets the higher their social value. This finding aligns with previous studies in which a word with higher socialness predicts faster RTs on lexical tasks (Diveica et al., 2023).

The facilitatory effect of the social dimension was hindered by right AIns stimulation (Fig. 7*A*): after real right stimulation, compared with sham, the higher social connotation no longer facilitated semantic decisions on the abstract triplets. Consistently, the higher the E-field induced in right AIns, the lower the facilitation caused by higher social scores (i.e., slower RTs and fewer correct responses; Fig. 7*C*, Extended Data Fig. 7-6*C*). The main effect of social dimension makes it unlikely that this interaction is due to the stimulation interfering with cognitive abilities in general: indeed, the triplets with higher social (and emotional) ratings benefit from that facilitatory effect, suggesting that higher scores on these dimensions facilitate semantic processing, and the TMS interferes particularly with these “easier” triplets.

Again, we only found an interaction with the social dimension, but not with the social category (Fig. 6), perhaps because a dimensional model is more fine-grained than a categorical one, therefore more powerful as it captures the overlap between emotional and social features.

The control analysis with concrete triplets did not show any significant results with social ratings either, showing that right AIns is primarily involved in the representation of social content of abstract concepts.

These findings align with an embodied view of the abstract domain (Desai et al., 2018), where the social content of abstract words is supported by the right AIns, which plays an important role in nonlinguistic social experience.

Literature supports a connection between social cognition and interoception (Pollatos et al., 2015; Arnold et al., 2019; Gao et al., 2019; Mattarozzi et al., 2019; Oldroyd et al., 2019; Arslanova et al., 2022; Feldman et al., 2023), whereby higher interoceptive abilities predict a more adaptive sociality. Together with the results from the heartbeat counting task, our findings support a convergence of interoceptive and social and emotional processes in the right AIns (Garfinkel and Critchley, 2013; Adolfi et al., 2017), wherein we hypothesize that interoceptive feedback informs semantic judgments providing a grounded representation of the social value of abstract concepts.

### Limitations

Although we reached AIns with the TMS, we certainly could not restrict the stimulation to this area (see MAX and PHC E-field analysis in Results), and we inevitably stimulated other brain regions that have contributed to the observed responses. AIns might also have been indirectly influenced by the other connected regions that were reached by rTMS.

Moreover, while our results suggest that there are neural resources inside AIns dedicated to the social and emotional components of language and to interoception, with our NIBS methods we do not know whether these resources consist of the same populations of neurons performing computations for both tasks or to different populations of neurons; therefore other methods, such as intracranial recordings, might clarify this.

Finally, our results were obtained using emotion and social-referring abstract concepts, and also in the light of critics expressed on the affective embodiment account (Winter, 2023), future studies are needed to clarify how much the importance of the emotion and social dimensions generalizes to other categories of abstract concepts, such as quantities or cognitive concepts (Villani et al., 2019; Persichetti et al., 2024).

### Conclusions

According to the embodied cognition theory, abstract concepts are perceptually grounded in interoception. This study investigated whether abstract concepts and interoception processing overlap in the Anterior Insula, leveraging the causal inference provided by TMS and E-field ROI analysis. Our findings revealed that the right AIns stimulation interfered with both interoception and social and emotional dimensions of abstract concepts. Our interoception findings confirm the role of the right AIns in cardiac interoceptive awareness. The semantic results provide evidence for the role of the right AIns in the processing of socio-emotional content of abstract concepts, in line with an embodied theory where abstract concepts engage areas representing the corresponding experience, challenging the dual coding theory claim that abstract concepts only rely on a linguistic format. Together, these results suggest that interoceptive and semantic processes converge on the right AIns, where we hypothesize that interoceptive states are integrated into semantic judgments and provide a perceptual, internal signature for emotional and social content.

## Data Availability

Data, stimulus words, and code have been deposited at the OSF repository (https://osf.io/cavsy/). The original MRI and the mesh files of the E-field simulations cannot be deposited in a public repository because of privacy reasons.
