# Supplementary figures and images for: Emotional and Social Dimension of Abstract Concepts Meet with Interoception in Right Anterior Insula
Source: J Neurosci. 2025 Nov 21;46(2):e0238252025. doi: 10.1523/JNEUROSCI.0238-25.2025 (PMC12809663; doi:10.1523/JNEUROSCI.0238-25.2025)

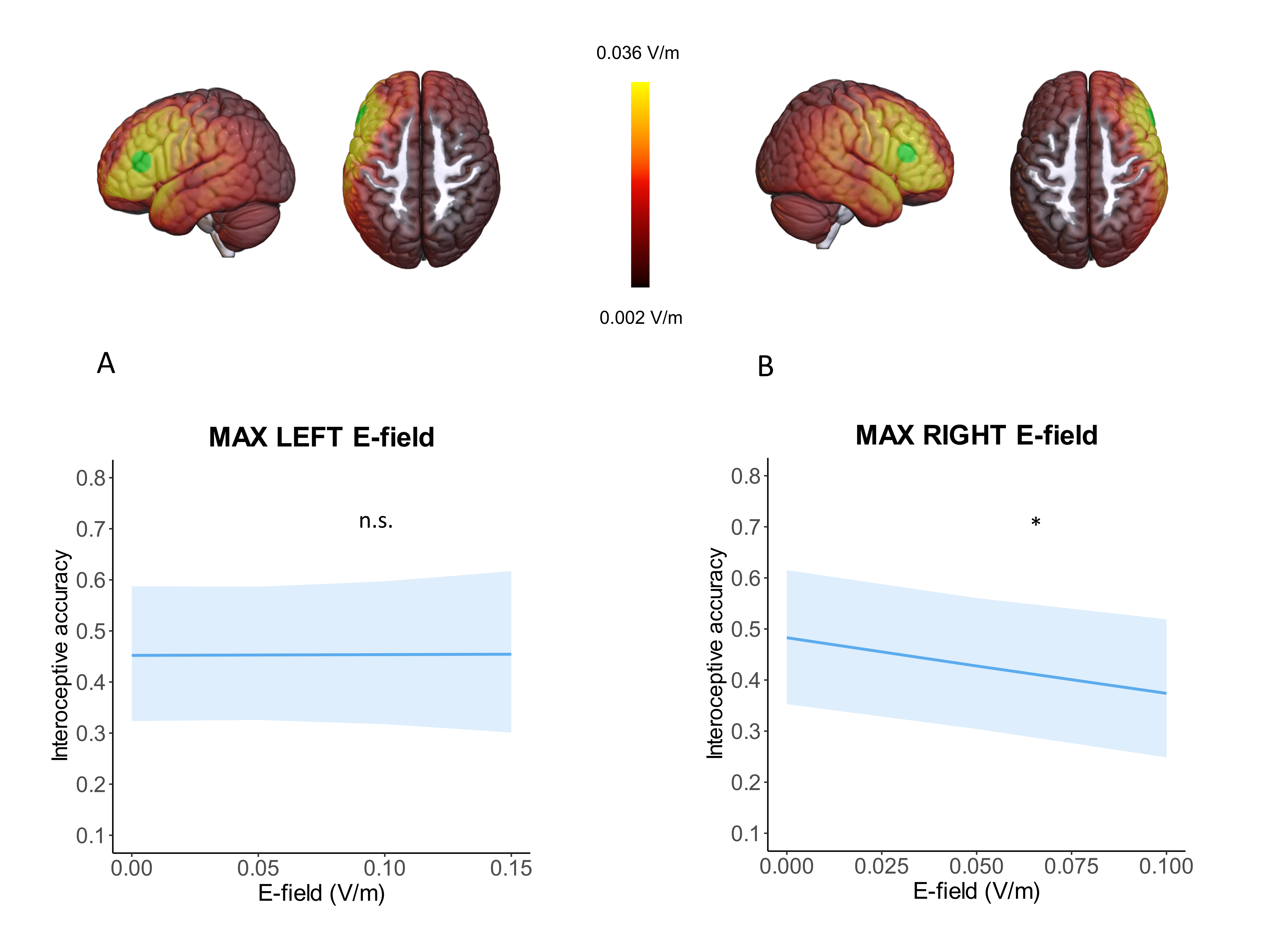

Supplement: Figure 5-1 — MAX E-field as predictor of Interoceptive accuracy MAX: maximum, E-field: electric field. (A) Adjusted predictions of the effect of the E-field induced in left MAX on interoceptive accuracy. The effect of left MAX E-field is not significant (Χ2 = 0.001, p = 0.970). (B) Adjusted predictions of the effect of the E-field induced in right MAX on interoceptive accuracy. The degree of right MAX E-field significantly lowers interoceptive accuracy: higher E-field values in right MAX led to lower interoceptive accuracy (Χ2 = 5.861, p = 0.015) (A-B) Error bars represent 95% confidence intervals (CI) of the adjusted predictions. Download Figure 5-1, TIF file. [file jneuro-46-e0238252025-s004.tif]

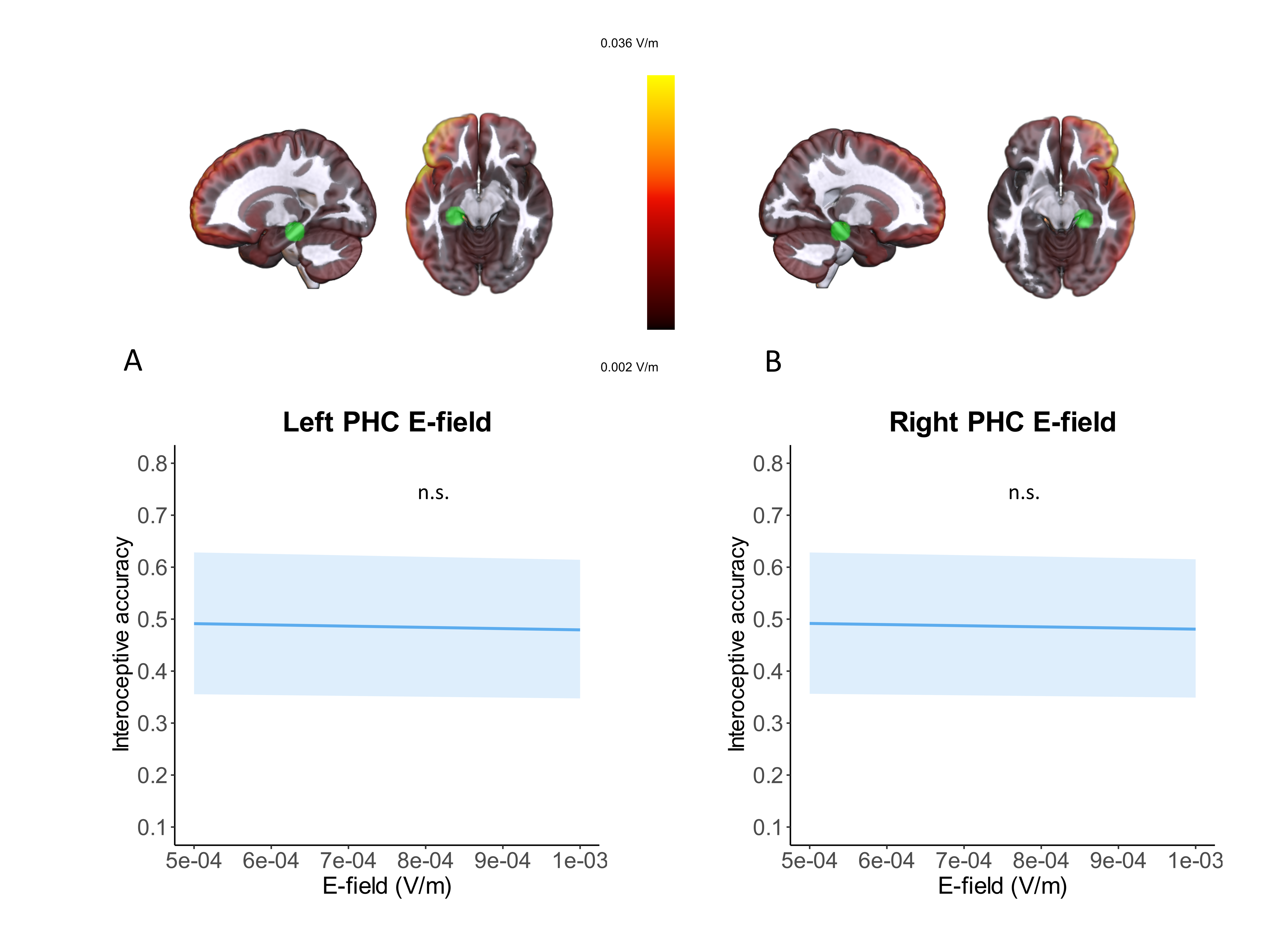

Supplement: Figure 5-2 — PHC E-field as predictor of Interoceptive accuracy PHC: parahippocampal cortex, E-field: electric field. (A) Adjusted predictions of the effect of the E-field induced in left PHC on interoceptive accuracy. The effect of left PHC E-field is not significant (Χ2 = 3.502, p = 0.061). (B) Adjusted predictions of the effect of the E-field induced in right PHC on interoceptive accuracy. The effect of right PHC E-field is not significant (Χ2 = 3.539, p = 0.060). (A-B) Error bars represent 95% confidence intervals (CI) of the adjusted predictions. Download Figure 5-2, TIF file. [file jneuro-46-e0238252025-s005.tif]

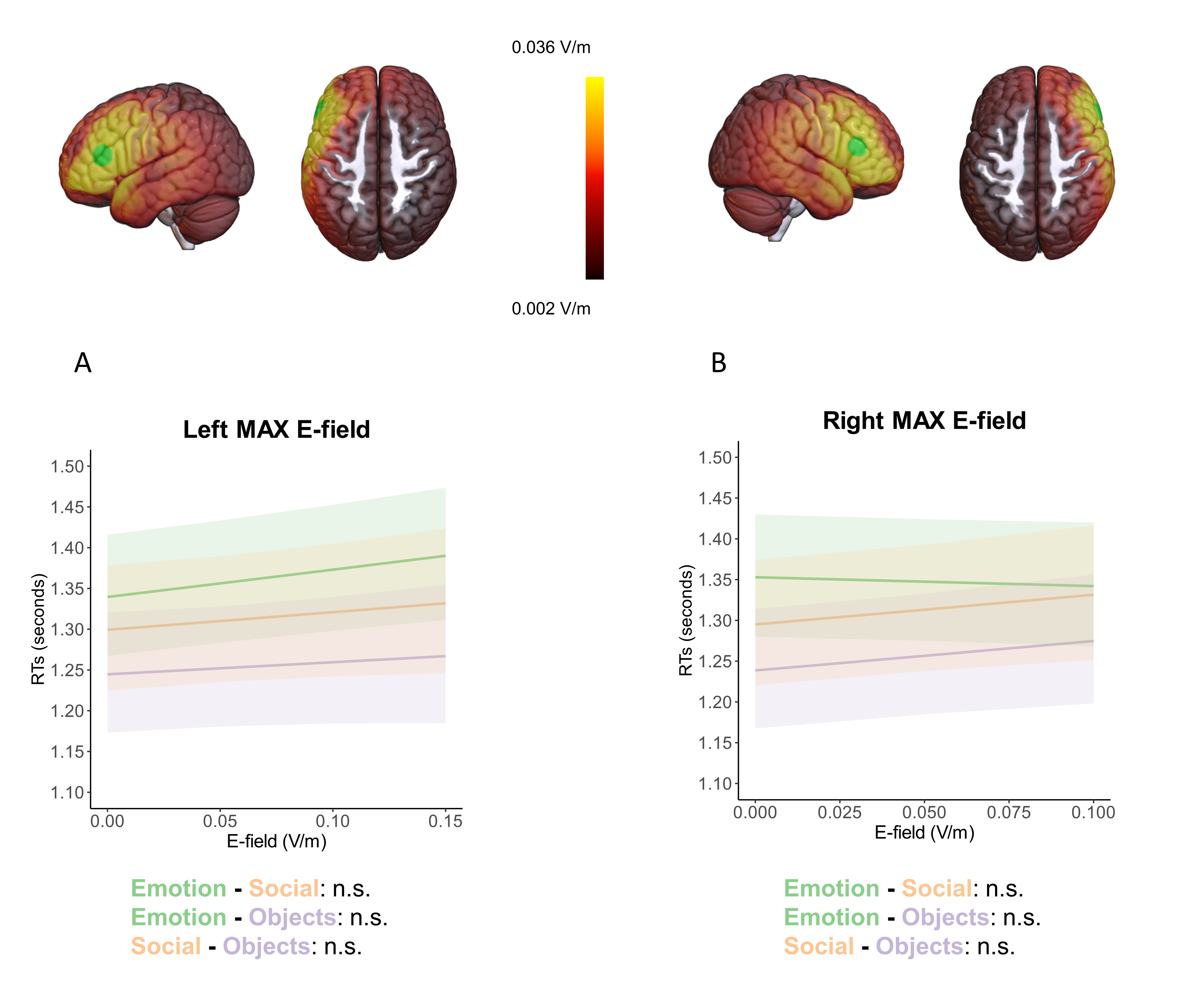

Supplement: Figure 6-4 — Interaction between MAX E-field and category as predictors of Reaction Times MAX: maximum, E-field: electric field. (A) Adjusted predictions of the interaction between the electric field (E-field) induced in left MAX and category on RTs. Planned comparisons were non-significant (Emotion-Social: t = 0.420, p = 1, Emotion-Objects: t = 0.668, p = 1, Social-Objects: t = 0.246, p = 1). (B) Adjusted predictions of the interaction between the electric field (E-field) induced in right MAX and category on RTs. Planned comparisons were non-significant (Emotion-Social: t = -1.756, p = 0.209, Emotion-Objects: t = -1.815, p = 0.209, Social-Objects: t = -0.051, p = 0.959). (A-B) Error bars represent 95% confidence interval (CI) of the adjusted predictions. All p values were corrected for multiple comparisons using Holm correction. n.s. p > 0.05. Download Figure 6-4, TIF file. [file jneuro-46-e0238252025-s009.tif]

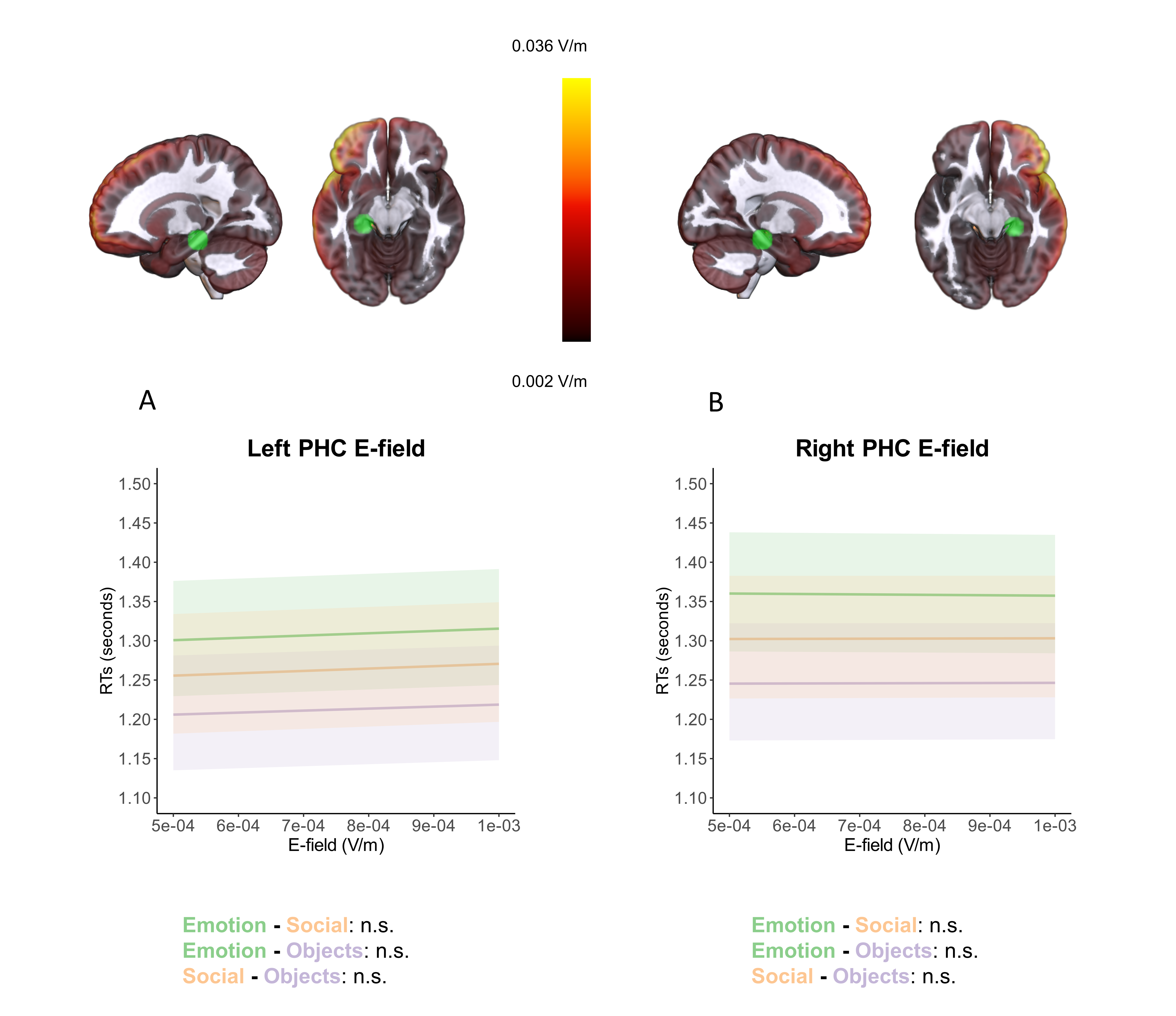

Supplement: Figure 6-5 — Interaction between PHC E-field and category as predictors of Reaction Times PHC: parahippocampal cortex, E-field: electric field. (A) Adjusted predictions of the interaction between the electric field (E-field) induced in left PHC and category on RTs. Planned comparisons were non-significant (Emotion-Social: t value = -0.241, p = 1, Emotion-Objects: t value = 0.258, p = 1, Social-Objects: t value: 0.508, p = 1). (B) Adjusted predictions of the interaction between the electric field (E-field) induced in right PHC and category on RTs. Planned comparisons were non-significant (Emotion-Social: t value = -1.193, p = 0.644, Emotion-Objects: t value = -1.241, p = 0.644, Social-Objects: t value: -0.048, p = 0.962). (A-B) Error bars represent 95% confidence interval (CI) of the adjusted predictions. All p values were corrected for multiple comparisons using Holm correction. n.s. p > 0.05. Download Figure 6-5, TIF file. [file jneuro-46-e0238252025-s010.tif]

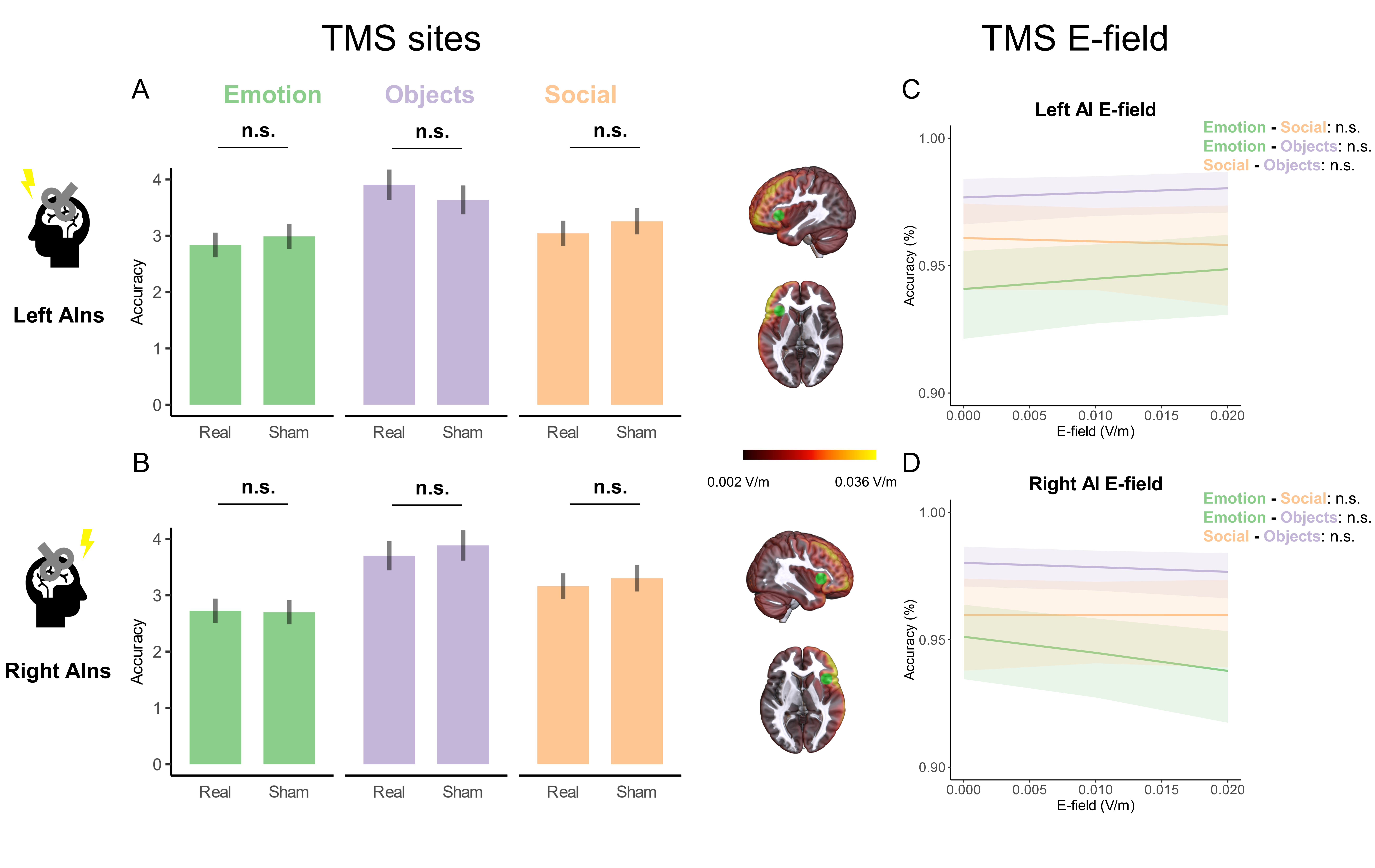

Supplement: Figure 6-9 — Semantic similarity task. Category results. Accuracy AIns: Anterior Insula, E-field: electric field (A-B) Estimated marginal means of Accuracy (on the logit scale) following left (A) and right (B) TMS conditions. Planned comparisons were non-significant. (C) Adjusted predictions of the interaction between the electric field (E-field) induced in left Anterior Insula (AIns) and category on Accuracy. Planned comparisons were non-significant. (D) Adjusted predictions of the interaction between the electric field (E-field) induced in right Anterior Insula (AIns) and category on Accuracy. Planned comparisons were non-significant. (A-B) Error bars represent standard errors of the marginal means. (C-D) Error bars represent 95% confidence interval (CI) of the adjusted predictions. All p values were corrected for multiple comparisons using Holm correction. n.s. p > 0.05. Download Figure 6-9, TIF file. [file jneuro-46-e0238252025-s014.tif]

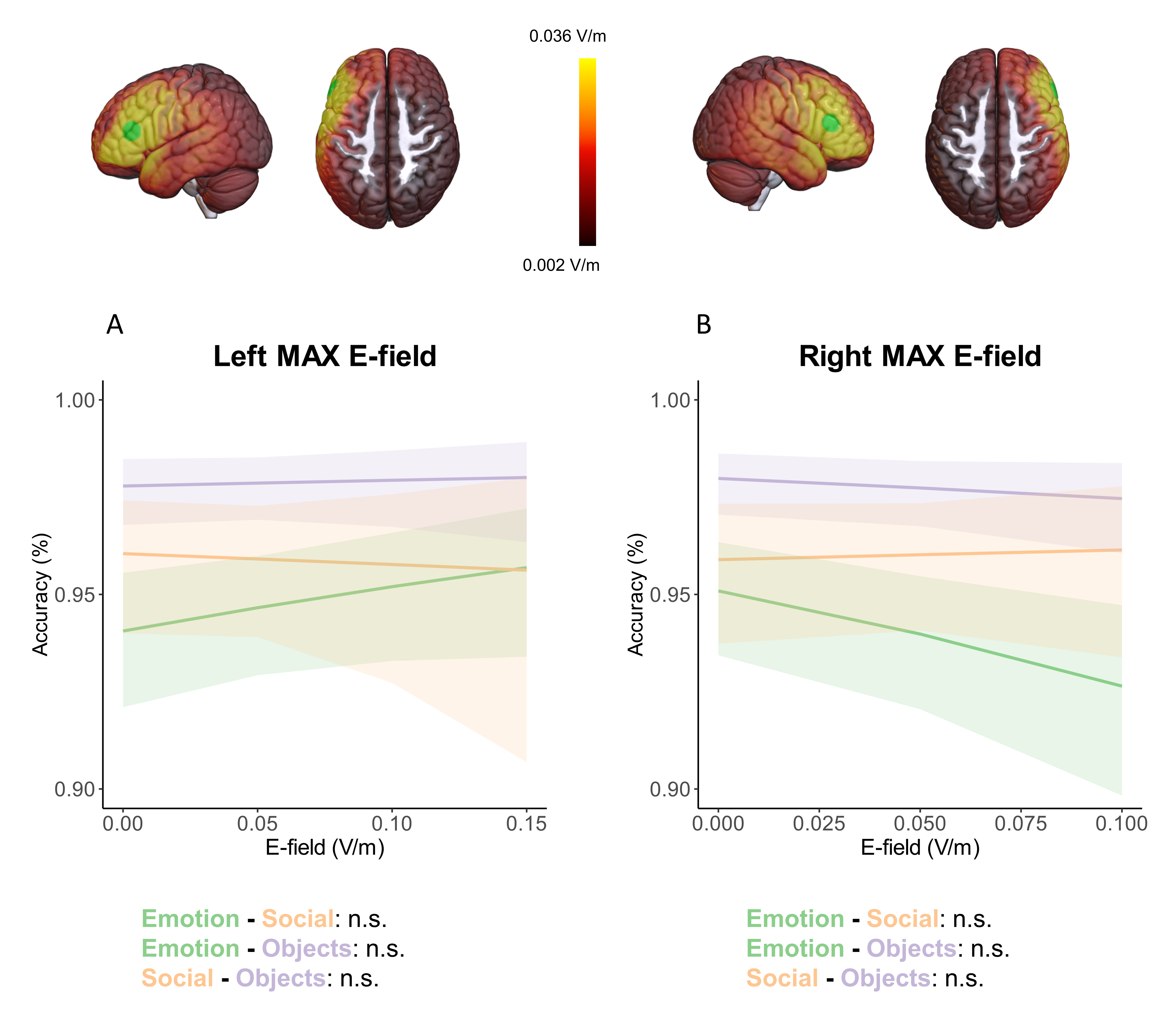

Supplement: Figure 6-10 — Interaction between MAX E-field and category as predictors of Accuracy MAX: maximum, E-field: electric field. (A) Adjusted predictions of the interaction between the electric field (E-field) induced in left MAX and category on Accuracy. Planned comparisons were non-significant (Emotion-Social: z value = 0.965, p = 1, Emotion-Objects: z value = 0.426, p = 1, Social-Objects: z value: -0.382, p = 1). (B) Adjusted predictions of the interaction between the electric field (E-field) induced in right MAX and category on Accuracy. Planned comparisons were non-significant (Emotion-Social: z value = -1.588, p = 0.337, Emotion-Objects: z value = -0.545, p = 0.864, Social-Objects: z value: 0.786, p = 0.864). (A-B) Error bars represent standard errors of the marginal means. (C-D) Error bars represent 95% confidence interval (CI) of the adjusted predictions. All p values were corrected for multiple comparisons using Holm correction. n.s. p > 0.05. Download Figure 6-10, TIF file. [file jneuro-46-e0238252025-s015.tif]

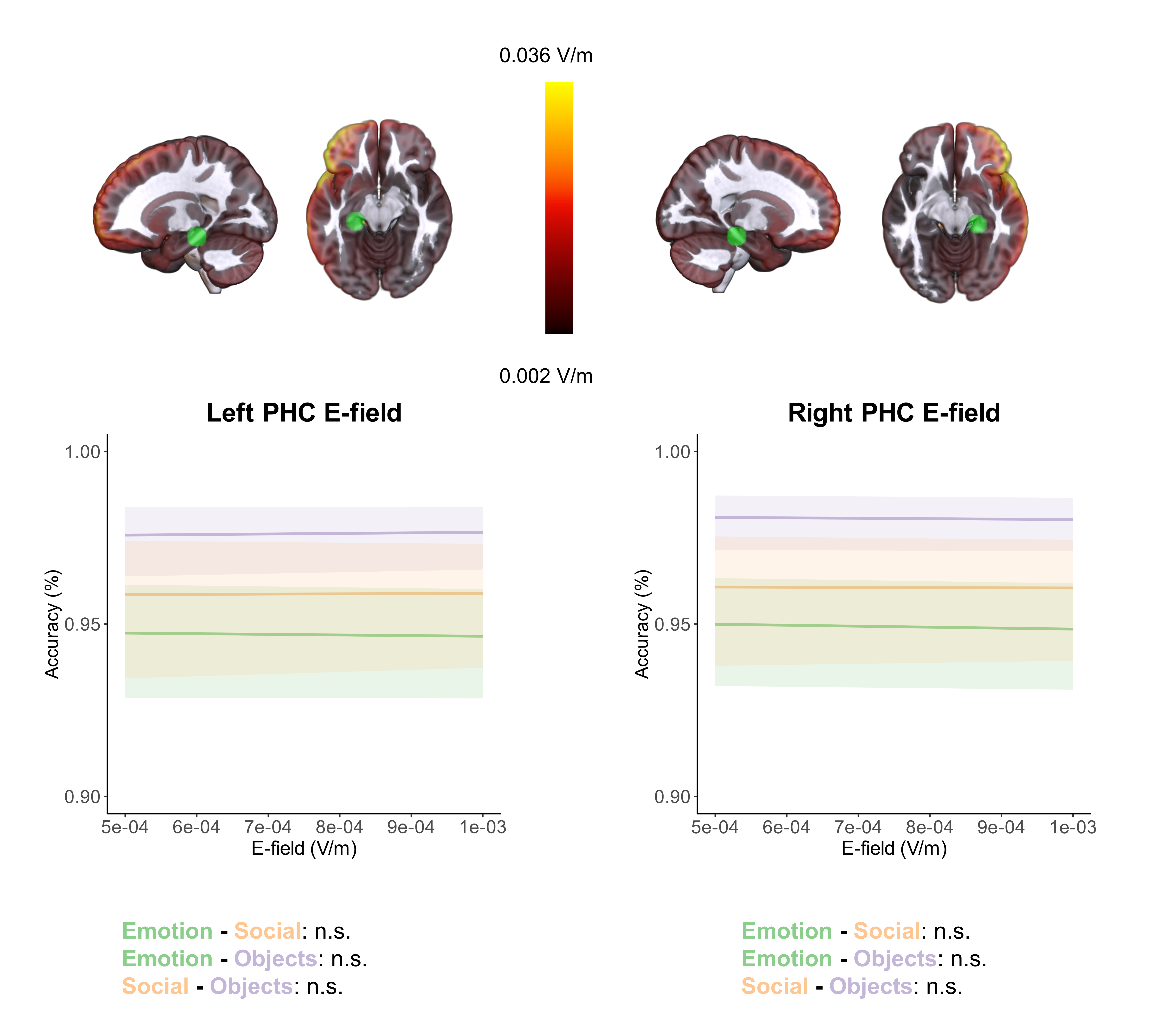

Supplement: Figure 6-11 — Interaction between PHC E-field and category as predictors of Accuracy PHC: parahippocampal cortex, E-field: electric field. (A) Adjusted predictions of the interaction between the electric field (E-field) induced in left PHC and category on Accuracy. Planned comparisons were non-significant (Emotion-Social: z value = -0.629, p = 1, Emotion-Objects: z value = -1.034, p = 0.903, Social-Objects: z value: -0.492, p = 1). (B) Adjusted predictions of the interaction between the electric field (E-field) induced in right PHC and category on Accuracy. Planned comparisons were non-significant (Emotion-Social: z value = -0.638, p = 1, Emotion-Objects: z value = 0.086, p = 1, Social-Objects: z value: 0.616, p = 1). Download Figure 6-11, TIF file. [file jneuro-46-e0238252025-s016.tif]

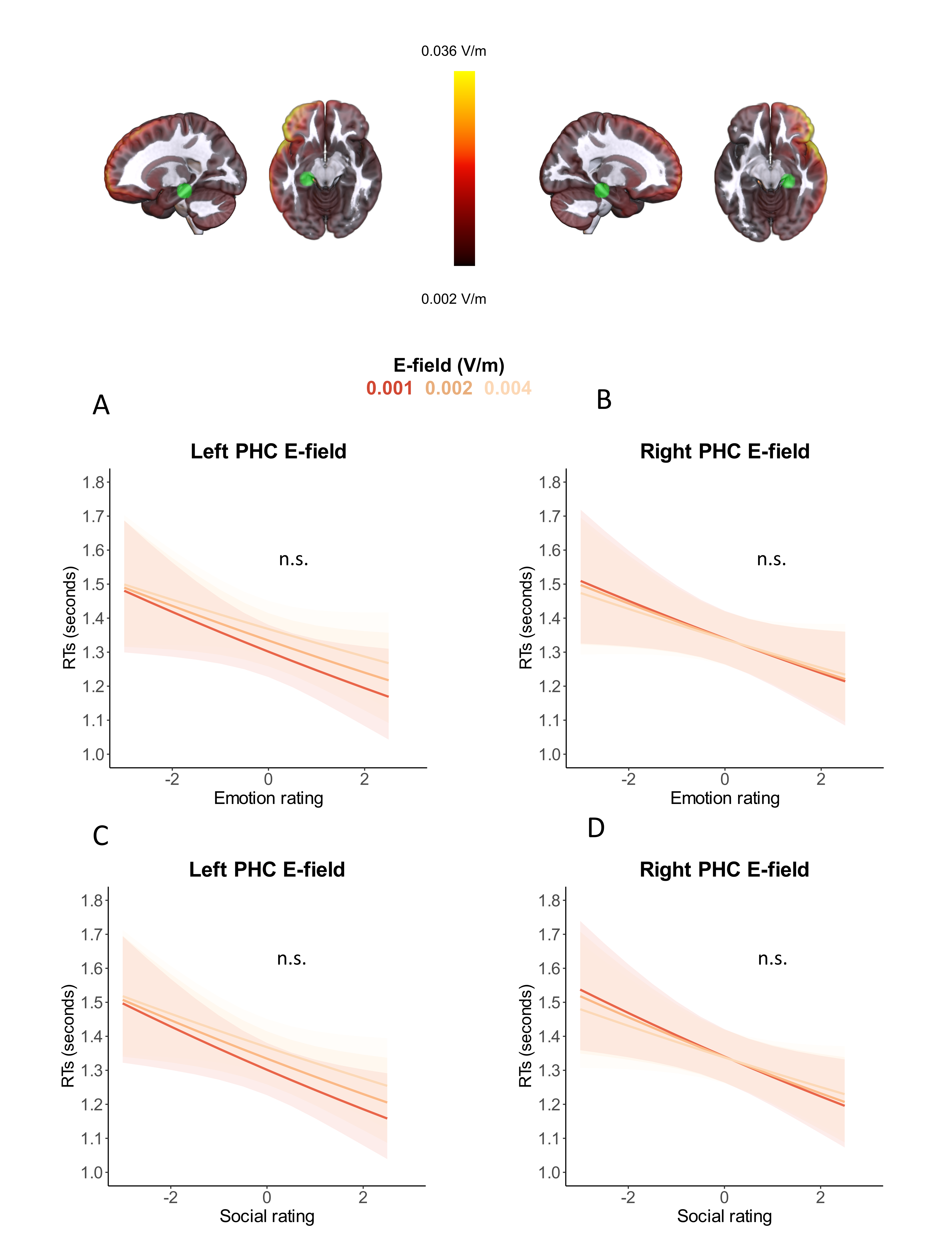

Supplement: Figure 7-2 — Interaction between PHC E-field and semantic ratings as predictors of Reaction Times of abstract triplets PHC: parahippocampal cortex, E-field: electric field. Results are shown on the centred Social and Emotion rating. (A, C) Adjusted predictions of the interaction between the left PHC E-field on Emotion (A) and Social (C) rating. The interactions are not significant (left PHC E-field*Emotion rating: F value = 1.146, p = 0.284, left PHC*Social rating: F value = 1.163, p = 0.281). (B, D) Adjusted predictions of the interaction between the right PHC E-field on Emotion (B) and Social (D) rating. Interactions are not significant (right PHC E-field*Emotion rating: F value = 0.281, p = 0.596, right PHC*Social rating: F value = 0.844, p = 0.358). Error bars represent 95% confidence intervals (CI) of the adjusted predictions. n.s. p > 0.05. Download Figure 7-2, TIF file. [file jneuro-46-e0238252025-s018.tif]

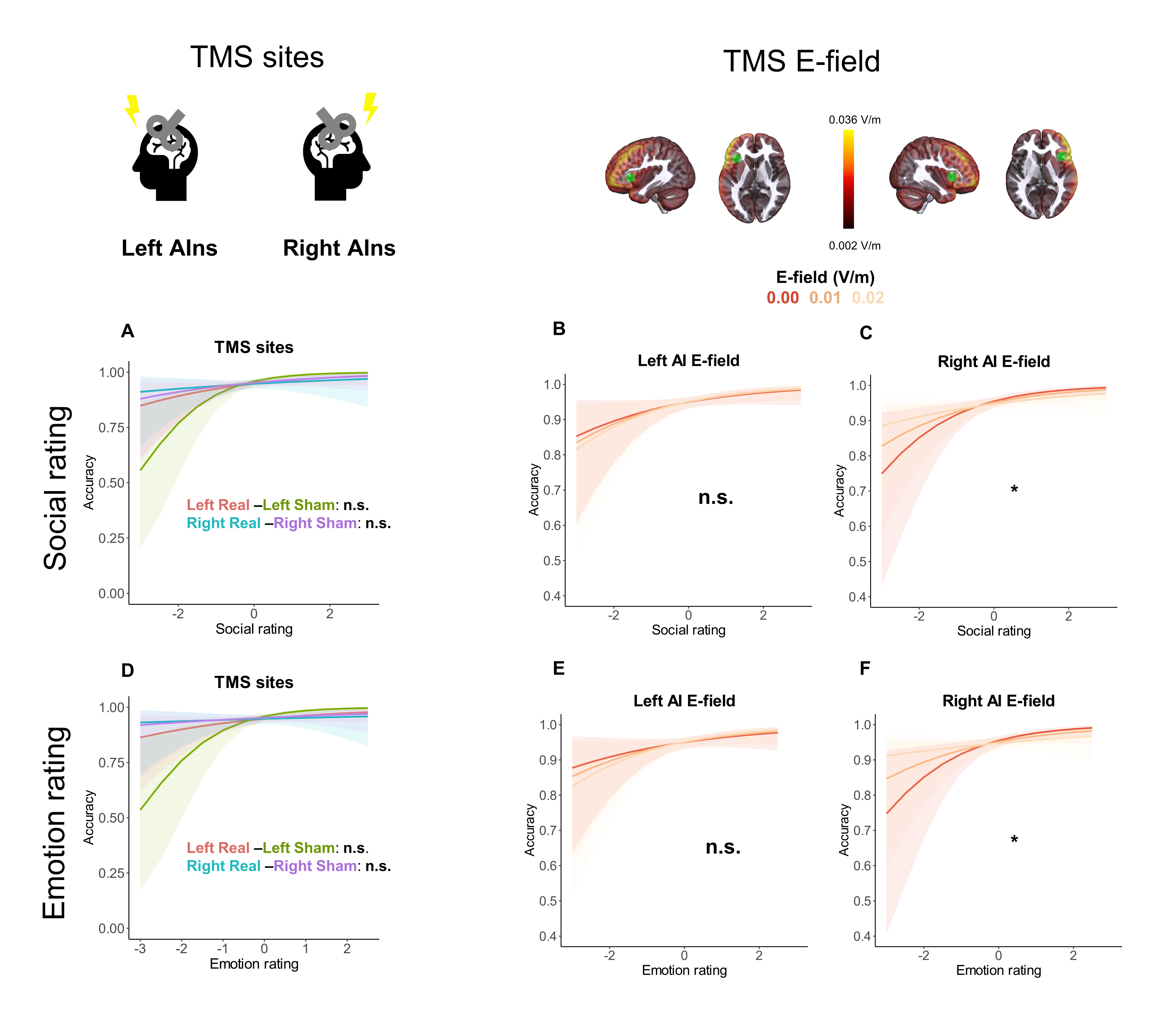

Supplement: Figure 7-6 — Semantic similarity task. Semantic ratings results with abstract triplets. Accuracy AIns: Anterior Insula, E-field: electric field. Results are shown on the centred Social and Emotion rating. (A, D) Adjusted predictions of Accuracy following each TMS condition, transformed from logit to probability scale. The comparisons between left real-left sham and between right real-right sham were not significant. P values of the planned comparisons were corrected for multiple comparisons using Holm correction. (B, E) Adjusted predictions of the interaction between the E-field induced in left AIns with Emotion (B) and Social (E) rating on Accuracy. The interactions are not significant. (C, F) Adjusted predictions of the interaction between the E-field induced in right AIns with Emotion (B) and Social (E) rating on Accuracy. Both interactions are significant and show a negative trend, whereby the higher E-field in the right AIns determines the lower probability of responding correctly to triplets the higher their emotion and social rating. Error bars represent 95% confidence intervals (CI) of the adjusted predictions. * p < 0.05, n.s. p > 0.05. Download Figure 7-6, TIF file. [file jneuro-46-e0238252025-s022.tif]

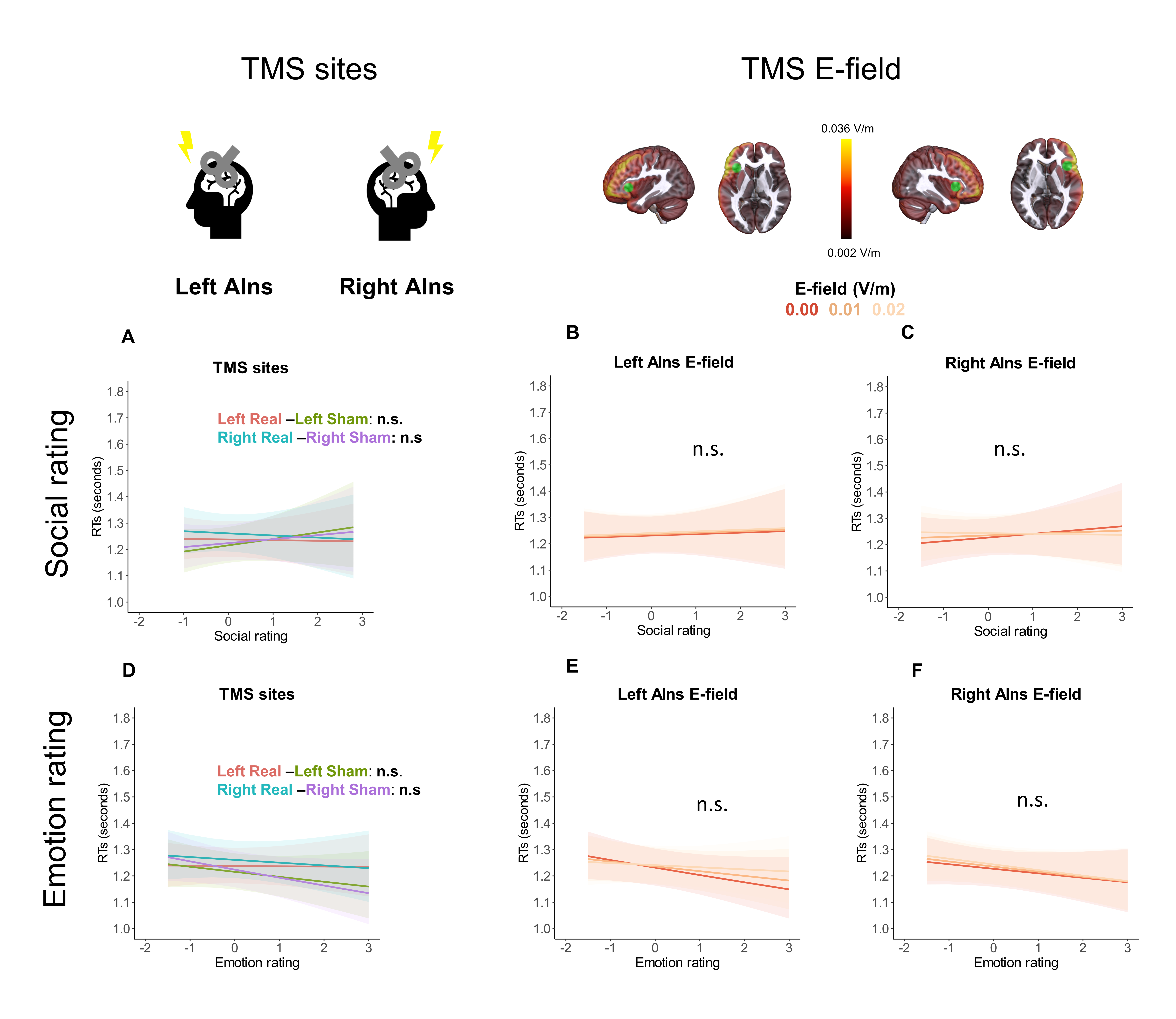

Supplement: Figure 7-12 — Semantic similarity task. Semantic ratings results with concrete triplets. RTs AIns: Anterior Insula, E-field: electric field. Results are shown on the centred Social and Emotion rating. (A, D) Adjusted predictions of reaction times (RTs) following each TMS condition, shown in the response scale. The comparisons between left real-left sham and between right real-right sham were not significant, meaning the TMS condition did not significantly change the effect of social or emotion rating on the RTs for concrete triplets. P values of the planned comparisons were corrected for multiple comparisons using Holm correction. (B, E) Adjusted predictions of the interaction between the E-field induced in left AIns on Social (B) and Emotion (E) rating. The interactions are not significant, meaning the magnitude of the E-field inside left AIns did not significantly change the effect of social or emotion rating on the RTs for concrete triplets. (C, F) Adjusted predictions of the interaction between the E-field induced in right AIns with Emotion (B) and Social (E) rating. Interactions are not significant, meaning the magnitude of the E-field inside right AIns did not significantly change the effect of social or emotion rating on the RTs for concrete triplets. Error bars represent 95% confidence intervals (CI) of the adjusted predictions. ** p < 0.01, * p < 0.05, n.s. p > 0.05. Download Figure 7-12, TIF file. [file jneuro-46-e0238252025-s028.tif]

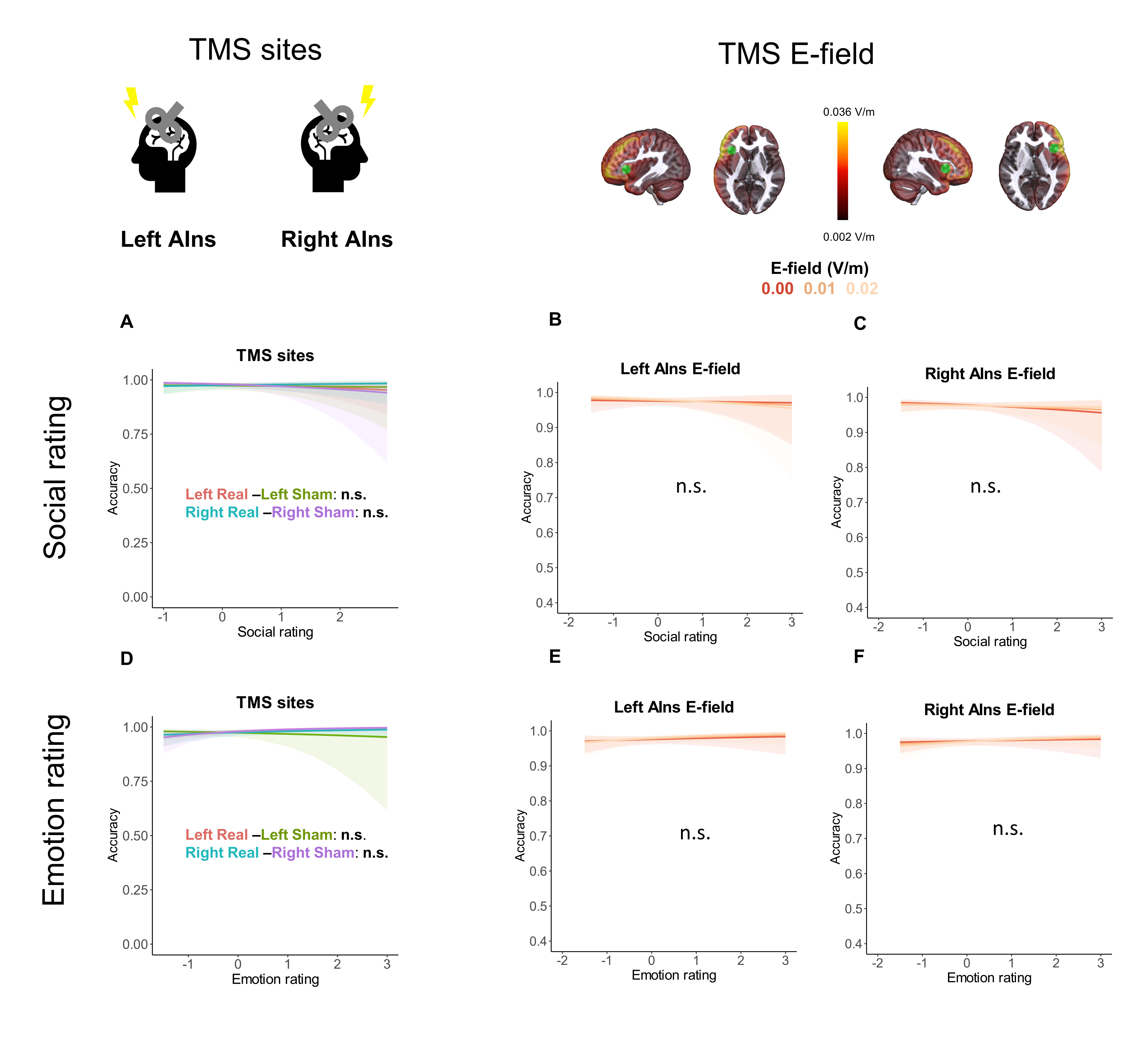

Supplement: Figure 7-16 — Semantic similarity task. Semantic ratings results with concrete triplets. Accuracy AIns: Anterior Insula, E-field: electric field. Results are shown on the centred Social and Emotion rating. (A, D) Adjusted predictions of Accuracy following each TMS condition, transformed from logit to probability scale. The comparisons between left real-left sham and between right real-right sham were not significant, meaning the TMS condition did not significantly change the effect of social or emotion rating on the accuracy for concrete triplets. P values of the planned comparisons were corrected for multiple comparisons using Holm correction. (B, E) Adjusted predictions of the interaction between the E-field induced in left AIns with Emotion (B) and Social (E) rating on Accuracy. The interactions are not significant, meaning the magnitude of the E-field inside left AIns did not significantly change the effect of social or emotion rating on the accuracy for concrete triplets (C, F) Adjusted predictions of the interaction between the E-field induced in right AIns with Emotion (B) and Social (E) rating on Accuracy. Interactions are not significant, meaning the magnitude of the E-field inside right AIns did not significantly change the effect of social or emotion rating on the accuracy for concrete triplets. Error bars represent 95% confidence intervals (CI) of the adjusted predictions. n.s. p > 0.05. Download Figure 7-16, TIF file. [file jneuro-46-e0238252025-s032.tif]
