# Supplementary material for: Emotional and Social Dimension of Abstract Concepts Meet with Interoception in Right Anterior Insula
Source: J Neurosci. 2025 Nov 21;46(2):e0238252025. doi: 10.1523/JNEUROSCI.0238-25.2025 (PMC12809663; doi:10.1523/JNEUROSCI.0238-25.2025)
Supplement: Table 2-1 — E-field in left Anterior Insula as predictor of Interoceptive accuracy. Mixed-effects regression model results of TMS E-field in left AIns as predictors of interoceptive accuracy. Significant effects are written in bold. Chisq: Chi-squared statistic, Df: degrees of freedom. Download Table 2-1, DOCX file. [file jneuro-46-e0238252025-s001.docx]

## Table 2-1. E-field in left Anterior Insula as predictor of Interoceptive accuracy.

| *Model results* |  |  |  |  |  |  |
| --- | --- | --- | --- | --- | --- | --- |
|  | *Chisq* | *Df* | *p-value* |  |  |  |
| (Intercept) | 0.487 | 1 | 0.485 |  |  |  |
| Left AIns MagnE E-field | 0.050 | 1 | 0.823 |  |  |  |
| **Heart rate** | **7.635** | **1** | **0.006** |  |  |  |
|  |  |  |  |  |  |  |

Mixed-effects model results of TMS E-field in left AIns as predictors of interoceptive accuracy. Significant effects are written in bold.

Chisq: Chi-squared statistic, Df: degrees of freedom
