# Supplementary material for: Emotional and Social Dimension of Abstract Concepts Meet with Interoception in Right Anterior Insula
Source: J Neurosci. 2025 Nov 21;46(2):e0238252025. doi: 10.1523/JNEUROSCI.0238-25.2025 (PMC12809663; doi:10.1523/JNEUROSCI.0238-25.2025)
Supplement: Figure 6-1 — Interaction between category and TMS site as predictors of Reaction Times. Mixed-effects regression model results of TMS site and category as predictors of (log-transformed) reaction times, and planned comparisons between ipsilateral real and sham stimulations, showing for each semantic category the difference in average between right real and right sham TMS conditions, and between left real and left sham TMS conditions. Significant results are written in bold. Sum.Sq: Sum of squares, Mean.Sq: Sum of squares / degrees of freedom, NumDF, df: Degrees of freedom, DenDF: Denominator degrees of Freedom, estimate: estimated value of the contrast, SE: standard error, t.ratio: test statistic Download Figure 6-1, DOCX file. [file jneuro-46-e0238252025-s006.docx]

## Figure 6-1. Interaction between category and TMS site as predictors of Reaction Times.

| *Model results* |  | |  | |  | |  | |  | |  |
| --- | --- | --- | --- | --- | --- | --- | --- | --- | --- | --- | --- |
|  | *Sum.Sq* | | *Mean.Sq* | | *NumDF* | | *DenDF* | | *F.value* | | *p-value* |
| **TMS site** | **1.177** | | **0.392** | | **3** | | **8169.676** | | **6.994** | | **0.000** |
| **category** | **1.102** | | **0.551** | | **2** | | **176.689** | | **9.817** | | **0.000** |
| **semantic similarity similars** | **0.578** | | **0.578** | | **1** | | **176.618** | | **10.294** | | **0.002** |
| semantic similarity distants | 0.028 | | 0.028 | | 1 | | 177.715 | | 0.493 | | 0.484 |
| **triplet length** | **0.322** | | **0.322** | | **1** | | **176.367** | | **5.745** | | **0.018** |
| TMS site:category | 0.093 | | 0.015 | | 6 | | 8170.305 | | 0.276 | | 0.948 |
| *Planned comparisons* | |  | |  | |  | |  | |  | |
| *contrast* | | *estimate* | | *SE* | | *df* | | *t.ratio* | | *p-value* | |
| Emotion Left Real - Left Sham | | 1.014 | | 0.013 | | 8205.338 | | 1.112 | | 0.475 | |
| Social Left Real - Left Sham | | 1.018 | | 0.013 | | 8198.664 | | 1.385 | | 0.475 | |
| Object Left Real - Left Sham | | 1.018 | | 0.013 | | 8198.395 | | 1.411 | | 0.475 | |
| Emotion Right Real - Right Sham | | 1.026 | | 0.013 | | 8180.070 | | 1.978 | | 0.192 | |
| Social Right Real - Right Sham | | 1.029 | | 0.013 | | 8179.276 | | 2.279 | | 0.123 | |
| Object Right Real - Right Sham | | 1.030 | | 0.013 | | 8179.980 | | 2.318 | | 0.123 | |

Mixed-effect regression model results of TMS site and category as predictors of (log-transformed) reaction times, and planned comparisons between ipsilateral real and sham stimulations, showing for each semantic category the difference in average between right real and right sham TMS conditions, and between left real and left sham TMS conditions. Significant results are written in bold.

Sum.Sq: Sum of squares, Mean.Sq: Sum of squares / degrees of freedom, NumDF, df: Degrees of freedom, DenDF: Denominator degrees of Freedom, estimate: estimated value of the contrast, SE: standard error, t.ratio: test statistic
