# Supplementary material for: Emotional and Social Dimension of Abstract Concepts Meet with Interoception in Right Anterior Insula
Source: J Neurosci. 2025 Nov 21;46(2):e0238252025. doi: 10.1523/JNEUROSCI.0238-25.2025 (PMC12809663; doi:10.1523/JNEUROSCI.0238-25.2025)
Supplement: Figure 6-2 — Interaction between category and E-field in left Anterior Insula as predictors of Reaction times. Mixed-effects regression model results of TMS E-field in left AIns and category as predictors of (log-transformed) reaction times, and planned comparisons to test for differences in the effects of E-field in left AIns between categories. Significant results are written in bold. Sum.Sq: Sum of squares, Mean.Sq: Sum of squares / degrees of freedom, NumDF, df: Degrees of freedom, DenDF: Denominator degrees of Freedom, estimate: estimated value of the contrast, SE: standard error, t.ratio: test statistics Download Figure 6-2, DOCX file. [file jneuro-46-e0238252025-s007.docx]

## Figure 6-2. Interaction between category and E-field in left Anterior Insula as predictors of Reaction times.

| *Model results* | |  | |  | |  | |  |  | |  | |
| --- | --- | --- | --- | --- | --- | --- | --- | --- | --- | --- | --- | --- |
|  | | *Sum.Sq* | | *Mean.Sq* | | *NumDF* | | *DenDF* | *F.value* | | *p-value* | |
| **Left AIns E-field** | | **0.413** | | **0.413** | | **1** | | **7845.968** | **7.352** | | **0.007** | |
| **category** | | **1.106** | | **0.553** | | **2** | | **176.786** | **9.858** | | **0.000** | |
| **semantic similarity similars** | | **0.541** | | **0.541** | | **1** | | **176.630** | **9.649** | | **0.002** | |
| semantic similarity distants | | 0.015 | | 0.015 | | 1 | | 177.888 | 0.267 | | 0.606 | |
| **triplet length** | | **0.317** | | **0.317** | | **1** | | **176.471** | **5.649** | | **0.019** | |
| Left AIns E-field:category | | 0.117 | | 0.059 | | 2 | | 7839.937 | 1.044 | | 0.352 | |
| *Planned comparisons* |  | |  | |  | |  | | |  | |  |
| *contrast* | *estimate* | | *SE* | | *df* | | *t.ratio* | | | *p-value* | |  |
| Emotion - Social | 0.714 | | 0.723 | | 7846.604 | | 0.988 | | | 0.647 | |  |
| Emotion - Objects | 1.009 | | 0.715 | | 7846.158 | | 1.411 | | | 0.475 | |  |
| Social - Objects | 0.295 | | 0.711 | | 7841.947 | | 0.414 | | | 0.679 | |  |

Mixed-effect regression model results of TMS E-field in left AIns and category as predictors of (log-transformed) reaction times, and planned comparisons to test for differences in the effects of E-field in left AIns between categories. Significant results are written in bold.

Sum.Sq: Sum of squares, Mean.Sq: Sum of squares / degrees of freedom, NumDF, df: Degrees of freedom, DenDF: Denominator degrees of Freedom, estimate: estimated value of the contrast, SE: standard error, t.ratio: test statistics
