# Supplementary material for: Emotional and Social Dimension of Abstract Concepts Meet with Interoception in Right Anterior Insula
Source: J Neurosci. 2025 Nov 21;46(2):e0238252025. doi: 10.1523/JNEUROSCI.0238-25.2025 (PMC12809663; doi:10.1523/JNEUROSCI.0238-25.2025)
Supplement: Figure 6-3 — Interaction between category and E-field in right Anterior Insula as predictors of Reaction times. Mixed-effect regression model results of TMS E-field in right AIns and category as predictors of (log-transformed) reaction times, and planned comparisons to test for differences in the effects of E-field in right AIns between categories. Significant results are written in bold. Sum.Sq: Sum of squares, Mean.Sq: Sum of squares / degrees of freedom, NumDF, df: Degrees of freedom, DenDF: Denominator degrees of Freedom, estimate: estimated value of the contrast, SE: standard error, t.ratio: test statistic. Download Figure 6-3, DOCX file. [file jneuro-46-e0238252025-s008.docx]

## Figure 6-3. Interaction between category and E-field in right Anterior Insula as predictors of Reaction times.

| *Model results* | |  | |  | |  |  |  | |  | |
| --- | --- | --- | --- | --- | --- | --- | --- | --- | --- | --- | --- |
|  | | *Sum.Sq* | | *Mean.Sq* | | *NumDF* | *DenDF* | *F.value* | | *p-value* | |
| Right AIns E-field | | 0.056 | | 0.056 | | 1 | 7837.694 | 1.001 | | 0.317 | |
| **category** | | **1.108** | | **0.554** | | **2** | **176.776** | **9.865** | | **0.000** | |
| **semantic similarity similars** | | **0.550** | | **0.550** | | **1** | **176.605** | **9.797** | | **0.002** | |
| semantic similarity distants | | 0.015 | | 0.015 | | 1 | 177.894 | 0.261 | | 0.610 | |
| **triplet length** | | **0.317** | | **0.317** | | **1** | **176.468** | **5.642** | | **0.019** | |
| Right AIns E-field:category | | 0.230 | | 0.115 | | 2 | 7830.225 | 2.047 | | 0.129 | |
| *Planned comparisons* |  | |  | |  | |  | |  | |  |
| *contrast* | *estimate* | | *SE* | | *df* | | *t.ratio* | | *p-value* | |  |
| Emotion - Social | -1.100 | | 0.628 | | 7836.549 | | -1.751 | | 0.232 | |  |
| Emotion - Objects | -1.104 | | 0.625 | | 7835.156 | | -1.768 | | 0.232 | |  |
| Social - Objects | -0.004 | | 0.618 | | 7834.162 | | -0.006 | | 0.995 | |  |

Mixed-effect regression model results of TMS E-field in right AIns and category as predictors of (log-transformed) reaction times, and planned comparisons to test for differences in the effects of E-field in right AIns between categories. Significant results are written in bold.

Sum.Sq: Sum of squares, Mean.Sq: Sum of squares / degrees of freedom, NumDF, df: Degrees of freedom, DenDF: Denominator degrees of Freedom, estimate: estimated value of the contrast, SE: standard error, t.ratio: test statistic
