# Supplementary material for: Emotional and Social Dimension of Abstract Concepts Meet with Interoception in Right Anterior Insula
Source: J Neurosci. 2025 Nov 21;46(2):e0238252025. doi: 10.1523/JNEUROSCI.0238-25.2025 (PMC12809663; doi:10.1523/JNEUROSCI.0238-25.2025)
Supplement: Figure 6-6 — Interaction between category and TMS site as predictors of Accuracy. Mixed-effect logistic regression model results of TMS site and category as predictors of accuracy, and planned comparisons between ipsilateral real and sham stimulations, for each semantic category. Significant results are written in bold. Chisq: Chi-squared statistic, Df: degrees of freedom, SE: standard error, z.ratio: test statistic Download Figure 6-6, DOCX file. [file jneuro-46-e0238252025-s011.docx]

## Figure 6-6. Interaction between category and TMS site as predictors of Accuracy.

| *Model results* |  | |  | |  | |  |  |
| --- | --- | --- | --- | --- | --- | --- | --- | --- |
|  | *Chisq* | | *Df* | | *p-value* | |  |  |
| **(Intercept)** | **492.904** | | **1** | | **0.000** | |  |  |
| TMS site | 0.772 | | 3 | | 0.856 | |  |  |
| **category** | **19.246** | | **2** | | **0.000** | |  |  |
| **semantic similarity similars** | **11.371** | | **1** | | **0.001** | |  |  |
| semantic similarity distants | 2.874 | | 1 | | 0.090 | |  |  |
| triplet length | 0.756 | | 1 | | 0.385 | |  |  |
| TMS site:category | 4.971 | | 6 | | 0.548 | |  |  |
| *Planned comparisons* | |  | |  | |  | |  |
| *contrast* | | *odds.ratio* | | *SE* | | *z.ratio* | | *p-value* |
| Emotion Left Real - Left Sham | | 0.858 | | 0.169 | | -0.781 | | 1.000 |
| Social Left Real - Left Sham | | 0.807 | | 0.170 | | -1.016 | | 1.000 |
| Object Left Real - Left Sham | | 1.307 | | 0.359 | | 0.975 | | 1.000 |
| Emotion Right Real - Right Sham | | 1.027 | | 0.189 | | 0.143 | | 1.000 |
| Social Right Real - Right Sham | | 0.868 | | 0.188 | | -0.654 | | 1.000 |
| Object Right Real - Right Sham | | 0.833 | | 0.229 | | -0.664 | | 1.000 |

Mixed-effects logistic regression model results of TMS site and category as predictors of accuracy, and planned comparisons between ipsilateral real and sham stimulations, for each semantic category. Significant results are written in bold.

Chisq: Chi-squared statistic, Df: degrees of freedom, SE: standard error, z.ratio: test statistic
