# Supplementary material for: Emotional and Social Dimension of Abstract Concepts Meet with Interoception in Right Anterior Insula
Source: J Neurosci. 2025 Nov 21;46(2):e0238252025. doi: 10.1523/JNEUROSCI.0238-25.2025 (PMC12809663; doi:10.1523/JNEUROSCI.0238-25.2025)
Supplement: Figure 6-7 — Interaction between category and E-field in right Anterior Insula as predictors of Accuracy. Mixed-effect logistic regression model results of TMS E-field in right AIns and category as predictors of accuracy, and planned comparisons to test for differences in the effects of E-field in right AIns between categories, showing the differences in the slope of the E-field effect on accuracy across categories. Significant results are written in bold. Chisq: Chi-squared statistic, Df: degrees of freedom, estimate: estimated value of the contrast, SE: standard error, z.ratio: test statistic Download Figure 6-7, DOCX file. [file jneuro-46-e0238252025-s012.docx]

## Figure 6-7. Interaction between category and E-field in right Anterior Insula as predictors of Accuracy.

| *Model results* | |  | |  | |  |  |  |
| --- | --- | --- | --- | --- | --- | --- | --- | --- |
|  | | *Chisq* | | *Df* | | *p-value* |  |  |
| **(Intercept)** | | **474.911** | | **1** | | **0.000** |  |  |
| Right AIns E-field | | 2.365 | | 1 | | 0.124 |  |  |
| **category** | | **19.796** | | **2** | | **0.000** |  |  |
| **semantic similarity similars** | | **12.586** | | **1** | | **0.000** |  |  |
| semantic similarity distants | | 3.514 | | 1 | | 0.061 |  |  |
| triplet length | | 1.023 | | 1 | | 0.312 |  |  |
| Right AIns E-field:category | | 1.814 | | 2 | | 0.404 |  |  |
| *Planned comparisons* |  | |  | |  | | |  |
| *contrast* | *estimate* | | *SE* | | *z.ratio* | | | *p-value* |
| Emotion - Social | -12.904 | | 9.611 | | -1.343 | | | 0.538 |
| Emotion - Objects | -4.523 | | 11.333 | | -0.399 | | | 0.957 |
| Social - Objects | 8.381 | | 11.828 | | 0.709 | | | 0.957 |

Mixed-effect logistic regression model results of TMS E-field in right AIns and category as predictors of accuracy, and planned comparisons to test for differences in the effects of E-field in right AIns between categories, showing the differences in the slope of the E-field effect on accuracy across categories. Significant results are written in bold.

Chisq: Chi-squared statistic, Df: degrees of freedom, estimate: estimated value of the contrast, SE: standard error, z.ratio: test statistic
