# Supplementary material for: Emotional and Social Dimension of Abstract Concepts Meet with Interoception in Right Anterior Insula
Source: J Neurosci. 2025 Nov 21;46(2):e0238252025. doi: 10.1523/JNEUROSCI.0238-25.2025 (PMC12809663; doi:10.1523/JNEUROSCI.0238-25.2025)
Supplement: Figure 6-8 — Interaction between category and E-field in left Anterior Insula as predictors of Accuracy. Mixed-effect logistic regression model results of TMS E-field in left AIns and category as predictors of accuracy, and planned comparisons to test for differences in the effects of E-field in left AIns between categories, showing the differences in the slope of the E-field effect on accuracy across categories. Significant effects are written in bold. Chisq: Chi-squared statistic, Df: degrees of freedom, estimate: estimated value of the contrast, SE: standard error, z.ratio: test statistic Download Figure 6-8, DOCX file. [file jneuro-46-e0238252025-s013.docx]

## Figure 6-8. Interaction between category and E-field in left Anterior Insula as predictors of Accuracy.

| *Model results* | |  | |  | |  |  |  |
| --- | --- | --- | --- | --- | --- | --- | --- | --- |
|  | | *Chisq* | | *Df* | | *p-value* |  |  |
| **(Intercept)** | | **474.879** | | **1** | | **0.000** |  |  |
| Left AIns E-field | | 0.636 | | 1 | | 0.425 |  |  |
| **category** | | **19.969** | | **2** | | **0.000** |  |  |
| **semantic similarity similars** | | **12.580** | | **1** | | **0.000** |  |  |
| semantic similarity distants | | 3.442 | | 1 | | 0.064 |  |  |
| triplet length | | 1.020 | | 1 | | 0.313 |  |  |
| Left AIns E-field:category | | 1.208 | | 2 | | 0.547 |  |  |
| *Planned comparisons* |  | |  | |  | | |  |
| *contrast* | *estimate* | | *SE* | | *z.ratio* | | | *p-value* |
| Emotion - Social | 10.921 | | 11.129 | | 0.981 | | | 0.979 |
| Emotion - Objects | -1.143 | | 12.997 | | -0.088 | | | 0.979 |
| Social - Objects | -12.064 | | 13.552 | | -0.890 | | | 0.979 |

Mixed-effect logistic regression model results of TMS E-field in left AIns and category as predictors of accuracy, and planned comparisons to test for differences in the effects of E-field in left AIns between categories, showing the differences in the slope of the E-field effect on accuracy across categories. Significant effects are written in bold.

Chisq: Chi-squared statistic, Df: degrees of freedom, estimate: estimated value of the contrast, SE: standard error, z.ratio: test statistic
