# Supplementary material for: Emotional and Social Dimension of Abstract Concepts Meet with Interoception in Right Anterior Insula
Source: J Neurosci. 2025 Nov 21;46(2):e0238252025. doi: 10.1523/JNEUROSCI.0238-25.2025 (PMC12809663; doi:10.1523/JNEUROSCI.0238-25.2025)
Supplement: Figure 7-3 — Interaction between semantic ratings and TMS site as predictors of Accuracy of Abstract triplets. Mixed-effect logistic regression model results of TMS site and semantic ratings as predictors of accuracy, and planned comparisons between ipsilateral real and sham stimulations, showing the difference in the average slope of emotion and social scores effect between right real and right sham TMS conditions, and between left real and left sham TMS conditions. Significant results are written in bold. Chisq: Chi-squared statistic, Df: degrees of freedom, estimate: estimated value of the contrast, SE: standard error, z.ratio: test statistic. Download Figure 7-3, DOCX file. [file jneuro-46-e0238252025-s019.docx]

## Figure 7-3. Interaction between semantic ratings and TMS site as predictors of Accuracy of Abstract triplets.

| *Model results* |  | | |  | |  |  | |  | |  |  |
| --- | --- | --- | --- | --- | --- | --- | --- | --- | --- | --- | --- | --- |
|  | *Chisq* | | | *Df* | | *p-value* |  |  |  |  |  |  |
| **(Intercept)** | **351.527** | | | **1** | | **0.000** |  |  |  |  |  |  |
| Emotion rating | 3.323 | | | 1 | | 0.068 |  |  |  |  |  |  |
| **Social rating** | **5.109** | | | **1** | | **0.024** |  |  |  |  |  |  |
| TMS site | 3.545 | | | 3 | | 0.315 |  |  |  |  |  |  |
| **semantic similarity similars** | **7.846** | | | **1** | | **0.005** |  |  |  |  |  |  |
| semantic similarity distants | 3.411 | | | 1 | | 0.065 |  |  |  |  |  |  |
| triplet length | 0.017 | | | 1 | | 0.896 |  |  |  |  |  |  |
| **Emotion rating:TMS site** | **10.404** | | | **3** | | **0.015** |  |  |  |  |  |  |
| **Social rating:TMS site** | **8.131** | | | **3** | | **0.043** |  |  |  |  |  |  |
| *Planned comparisons* | |  |  | |  | | |  | |  | | |
| *Emotion rating and TMS site* | |  |  | |  | | |  | |  | | |
| *contrast* | | *estimate* | *SE* | | *z.ratio* | | | *p-value* | |  |  |  |
| Left Real – Left Sham | | -0.653 | 0.308 | | -2.119 | | | 0.068 | |  |  |  |
| Right Real – Right Sham | | -0.090 | 0.296 | | -0.305 | | | 0.760 | |  |  |  |
| *Social rating and TMS site* | |  |  | |  | | |  | |  |  |  |
| *contrast* | | *estimate* | *SE* | | *z.ratio* | | | *p-value* | |  |  |  |
| Left Real – Left Sham | | -0.583 | 0.293 | | -1.988 | | | 0.094 | |  |  |  |
| Right Real – Right Sham | | -0.148 | 0.282 | | -0.525 | | | 0.600 | |  |  |  |

Mixed-effects logistic regression model results of TMS site and semantic ratings as predictors of accuracy, and planned comparisons between ipsilateral real and sham stimulations, showing the difference in the average slope of emotion and social scores effect between right real and right sham TMS conditions, and between left real and left sham TMS conditions. Significant results are written in bold.

Chisq: Chi-squared statistic, Df: degrees of freedom, estimate: estimated value of the contrast, SE: standard error, z.ratio: test statistic
