# Supplementary material for: Emotional and Social Dimension of Abstract Concepts Meet with Interoception in Right Anterior Insula
Source: J Neurosci. 2025 Nov 21;46(2):e0238252025. doi: 10.1523/JNEUROSCI.0238-25.2025 (PMC12809663; doi:10.1523/JNEUROSCI.0238-25.2025)
Supplement: Figure 7-4 — Interaction between semantic ratings and E-field in right Anterior Insula as predictors of Accuracy of Abstract triplets. Mixed-effects logistic regression model results of TMS E-field in right AIns and semantic ratings as predictors of accuracy, where the last two rows represent the interaction between the magnitude of the E-field inside right AIns and respectively emotion and social rating. Significant effects are written in bold. Chisq: Chi-squared statistic, Df: degrees of freedom. Download Figure 7-4, DOCX file. [file jneuro-46-e0238252025-s020.docx]

## Figure 7-4. Interaction between semantic ratings and E-field in right Anterior Insula as predictors of Accuracy of Abstract triplets.

| *Model results* |  |  |  |
| --- | --- | --- | --- |
|  | *Chisq* | *Df* | *p-value* |
| **(Intercept)** | **340.264** | **1** | **0.000** |
| Right AIns E-field | 2.547 | 1 | 0.111 |
| **Emotion rating** | **3.508** | **1** | **0.061** |
| **Social rating** | **5.039** | **1** | **0.025** |
| **semantic similarity similars** | **9.313** | **1** | **0.002** |
| **semantic similarity distants** | **4.237** | **1** | **0.040** |
| triplet length | 0.032 | 1 | 0.859 |
| **Right AIns E-field:Emotion rating** | **6.132** | **1** | **0.013** |
| **Right AIns E-field:Social rating** | **4.252** | **1** | **0.039** |

Mixed-effects logistic regression model results of TMS E-field in right AIns and semantic ratings as predictors of accuracy, where the last two rows represent the interaction between the magnitude of the E-field inside right AIns and respectively emotion and social rating. Significant effects are written in bold.

Chisq: Chi-squared statistic, Df: degrees of freedom
