# Supplementary material for: Emotional and Social Dimension of Abstract Concepts Meet with Interoception in Right Anterior Insula
Source: J Neurosci. 2025 Nov 21;46(2):e0238252025. doi: 10.1523/JNEUROSCI.0238-25.2025 (PMC12809663; doi:10.1523/JNEUROSCI.0238-25.2025)
Supplement: Figure 7-5 — Interaction between semantic ratings and E-field in left Anterior Insula as predictors of Accuracy of Abstract triplets. Mixed-effects logistic regression model results of TMS E-field in left AIns and semantic ratings as predictors of accuracy, where the last two rows represent the interaction between the magnitude of the E-field inside left AIns and respectively emotion and social rating. Significant effects are written in bold. Chisq: Chi-squared statistic, Df: degrees of freedom. Download Figure 7-5, DOCX file. [file jneuro-46-e0238252025-s021.docx]

## Figure 7-5. Interaction between semantic ratings and E-field in left Anterior Insula as predictors of Accuracy of Abstract triplets.

| *Model results* |  |  |  |
| --- | --- | --- | --- |
|  | *Chisq* | *Df* | *p-value* |
| **(Intercept)** | **341.337** | **1** | **0.000** |
| Left AIns E-field | 0.052 | 1 | 0.820 |
| Emotion rating | 3.191 | 1 | 0.074 |
| **Social rating** | **4.662** | **1** | **0.031** |
| **semantic similarity similars** | **9.228** | **1** | **0.002** |
| **semantic similarity distants** | **4.285** | **1** | **0.038** |
| triplet length | 0.042 | 1 | 0.837 |
| Left AIns E-field:Emotion rating | 0.463 | 1 | 0.496 |
| Left AIns E-field:Social rating | 0.232 | 1 | 0.630 |

Mixed-effects logistic regression model results of TMS E-field in left AIns and semantic ratings as predictors of accuracy, where the last two rows represent the interaction between the magnitude of the E-field inside left AIns and respectively emotion and social rating. Significant effects are written in bold.

Chisq: Chi-squared statistic, Df: degrees of freedom
