# Supplementary material for: Emotional and Social Dimension of Abstract Concepts Meet with Interoception in Right Anterior Insula
Source: J Neurosci. 2025 Nov 21;46(2):e0238252025. doi: 10.1523/JNEUROSCI.0238-25.2025 (PMC12809663; doi:10.1523/JNEUROSCI.0238-25.2025)
Supplement: Figure 7-9 — Interaction between TMS site and semantic ratings as predictors of Reaction times of Concrete triplets. Mixed-effect regression model results of TMS site and semantic ratings as predictors of (log-transformed) reaction times to concrete triplets, and planned comparisons between ipsilateral real and sham stimulations, showing the difference in the average slope of emotion and social scores effect between right real and right sham TMS conditions, and between left real and left sham TMS conditions. Significant results are written in bold. Sum.Sq: Sum of squares, Mean.Sq: Sum of squares / degrees of freedom, NumDF, df: Degrees of freedom, DenDF: Denominator degrees of Freedom, estimate: estimated value of the contrast, SE: standard error, t.ratio: test statistics. Download Figure 7-9, TIF file. [file jneuro-46-e0238252025-s025.docx]

## Figure 7-9. Interaction between TMS site and semantic ratings as predictors of Reaction times of Concrete triplets.

| *Model results* |  |  |  |  |  |  |
| --- | --- | --- | --- | --- | --- | --- |
|  | *Sum.Sq* | *Mean.Sq* | *NumDF* | *DenDF* | *F.value* | *p value* |
| Emotion_rating | 0.045 | 0.045 | 1 | 58.444 | 0.898 | 0.347 |
| Social_rating | 0.006 | 0.006 | 1 | 58.676 | 0.113 | 0.738 |
| **TMS_session** | **0.548** | **0.183** | **3** | **2795.148** | **3.682** | **0.012** |
| semantic similarity similars | 0.166 | 0.166 | 1 | 58.742 | 3.346 | 0.072 |
| semantic similarity distants | 0.068 | 0.068 | 1 | 58.772 | 1.373 | 0.246 |
| triplet length | 0.059 | 0.059 | 1 | 58.946 | 1.194 | 0.279 |
| Emotion_rating:TMS_session | 0.150 | 0.050 | 3 | 2793.051 | 1.010 | 0.387 |
| Social_rating:TMS_session | 0.127 | 0.042 | 3 | 2794.523 | 0.853 | 0.465 |

*Planned comparisons*

*Emotion ratings and TMS site*

| *contrast* | *estimate* | *SE* | *df* | *t.ratio* | *p.value* |
| --- | --- | --- | --- | --- | --- |
| Left Real-Left Sham | 0.015 | 0.015 | 2806.852 | 1.009 | 0.500 |
| Right Real-Right Sham | 0.017 | 0.015 | 2802.993 | 1.151 | 0.500 |

*Social ratings and TMS site*

| *contrast* | *estimate* | *SE* | *df* | *t.ratio* | *p.value* |
| --- | --- | --- | --- | --- | --- |
| Left Real-Left Sham | -0.022 | 0.019 | 2812.013 | -1.161 | 0.492 |
| Right Real -Right Sham | -0.019 | 0.018 | 2801.795 | -1.009 | 0.492 |

Mixed-effect regression model results of TMS site and semantic ratings as predictors of (log-transformed) reaction times to concrete triplets, and planned comparisons between ipsilateral real and sham stimulations, showing the difference in the average slope of emotion and social scores effect between right real and right sham TMS conditions, and between left real and left sham TMS conditions. Significant results are written in bold.

Sum.Sq: Sum of squares, Mean.Sq: Sum of squares / degrees of freedom, NumDF, df: Degrees of freedom, DenDF: Denominator degrees of Freedom, estimate: estimated value of the contrast, SE: standard error, t.ratio: test statistics
