# Supplementary material for: Emotional and Social Dimension of Abstract Concepts Meet with Interoception in Right Anterior Insula
Source: J Neurosci. 2025 Nov 21;46(2):e0238252025. doi: 10.1523/JNEUROSCI.0238-25.2025 (PMC12809663; doi:10.1523/JNEUROSCI.0238-25.2025)
Supplement: Figure 7-10 — Interaction between semantic ratings and E-field in right Anterior Insula as predictors of Reaction times of Concrete triplets. Mixed-effect regression model results of TMS E-field in right AIns and semantic ratings as predictors of (log-transformed) reaction times to concrete triplets, where the last two rows represent the interaction between the magnitude of the E-field inside right AIns and respectively emotion and social rating. Significant effects are written in bold. Sum.Sq: Sum of squares, Mean.Sq: Sum of squares / degrees of freedom, NumDF: Degrees of freedom, DenDF: Denominator degrees of Freedom. Download Figure 7-10, DOCX file. [file jneuro-46-e0238252025-s026.docx]

## Figure 7-10. Interaction between semantic ratings and E-field in right Anterior Insula as predictors of Reaction times of Concrete triplets.

*Model summary*

|  | *Sum.Sq* |  | *Mean.Sq* | *NumDF* | *DenDF* | *F.value* | *p value* |
| --- | --- | --- | --- | --- | --- | --- | --- |
| Right AIns E-field | 0.128 |  | 0.128 | 1 | 2686.189 | 2.581 | 0.108 |
| Emotion_rating | 0.049 |  | 0.049 | 1 | 58.422 | 0.985 | 0.325 |
| Social_rating | 0.003 |  | 0.003 | 1 | 58.633 | 0.068 | 0.795 |
| semantic similarity similars | 0.147 |  | 0.147 | 1 | 58.696 | 2.954 | 0.091 |
| semantic similarity distants | 0.060 |  | 0.060 | 1 | 58.703 | 1.204 | 0.277 |
| triplet length | 0.059 |  | 0.059 | 1 | 58.916 | 1.196 | 0.278 |
| Right AIns E-field:Emotion_rating | 0.001 |  | 0.001 | 1 | 2678.569 | 0.021 | 0.884 |
| Right AIns E-field:Social_rating | 0.099 |  | 0.099 | 1 | 2678.088 | 1.987 | 0.159 |

Mixed-effect regression model results of TMS E-field in right AIns and semantic ratings as predictors of (log-transformed) reaction times to concrete triplets, where the last two rows represent the interaction between the magnitude of the E-field inside right AIns and respectively emotion and social rating. Significant effects are written in bold.

Sum.Sq: Sum of squares, Mean.Sq: Sum of squares / degrees of freedom, NumDF: Degrees of freedom, DenDF: Denominator degrees of Freedom
