# Supplementary material for: Emotional and Social Dimension of Abstract Concepts Meet with Interoception in Right Anterior Insula
Source: J Neurosci. 2025 Nov 21;46(2):e0238252025. doi: 10.1523/JNEUROSCI.0238-25.2025 (PMC12809663; doi:10.1523/JNEUROSCI.0238-25.2025)
Supplement: Figure 7-11 — Interaction between semantic ratings and E-field in left Anterior Insula as predictors of Reaction times of Concrete triplets. Mixed-effect regression model results of TMS E-field in left AIns and semantic ratings as predictors of (log-transformed) reaction times to concrete triplets, where the last two rows represent the interaction between the magnitude of the E-field inside left AIns and respectively emotion and social rating. Significant effects are written in bold. Sum.Sq: Sum of squares, Mean.Sq: Sum of squares / degrees of freedom, NumDF: Degrees of freedom, DenDF: Denominator degrees of Freedom. Download Figure 7-11, DOCX file. [file jneuro-46-e0238252025-s027.docx]

## Figure 7-11. Interaction between semantic ratings and E-field in left Anterior Insula as predictors of Reaction times of Concrete triplets.

|  | Sum.Sq | Mean.Sq | NumDF | DenDF | F.value | p value |
| --- | --- | --- | --- | --- | --- | --- |
| Left AIns E-field | 0.031 | 0.031 | 1 | 2688.058 | 0.633 | 0.426 |
| Emotion_rating | 0.050 | 0.050 | 1 | 58.416 | 1.000 | 0.321 |
| Social_rating | 0.003 | 0.003 | 1 | 58.635 | 0.070 | 0.792 |
| semantic similarity similars | 0.146 | 0.146 | 1 | 58.696 | 2.937 | 0.092 |
| semantic similarity distants | 0.059 | 0.059 | 1 | 58.710 | 1.191 | 0.280 |
| triplet length | 0.059 | 0.059 | 1 | 58.915 | 1.177 | 0.282 |
| Left AIns E-field:Emotion_rating | 0.093 | 0.093 | 1 | 2680.186 | 1.878 | 0.171 |
| Left AIns E-field:Social_rating | 0.000 | 0.000 | 1 | 2682.959 | 0.010 | 0.921 |

Mixed-effect regression model results of TMS E-field in left AIns and semantic ratings as predictors of (log-transformed) reaction times to concrete triplets, where the last two rows represent the interaction between the magnitude of the E-field inside left AIns and respectively emotion and social rating. Significant effects are written in bold.

Sum.Sq: Sum of squares, Mean.Sq: Sum of squares / degrees of freedom, NumDF: Degrees of freedom, DenDF: Denominator degrees of Freedom
