# Supplementary material for: Emotional and Social Dimension of Abstract Concepts Meet with Interoception in Right Anterior Insula
Source: J Neurosci. 2025 Nov 21;46(2):e0238252025. doi: 10.1523/JNEUROSCI.0238-25.2025 (PMC12809663; doi:10.1523/JNEUROSCI.0238-25.2025)
Supplement: Figure 7-14 — Interaction between semantic ratings and E-field in right Anterior Insula as predictors of Accuracy of Concrete triplets. Mixed-effects logistic regression model results of TMS E-field in right AIns and semantic ratings as predictors of accuracy to concrete triplets, where the last two rows represent the interaction between the magnitude of the E-field inside right AIns and respectively emotion and social rating. Significant effects are written in bold. Chisq: Chi-squared statistic, Df: degrees of freedom. Download Figure 7-14, DOCX file. [file jneuro-46-e0238252025-s030.docx]

## Figure 7-14. Interaction between semantic ratings and E-field in right Anterior Insula as predictors of Accuracy of Concrete triplets.

|  | *Chisq* | *Df* | *p value* |
| --- | --- | --- | --- |
| (Intercept) | 296.364 | 1 | 0.000 |
| Right AIns E-field | 0.153 | 1 | 0.696 |
| Emotion_rating | 0.899 | 1 | 0.343 |
| Social_rating | 0.259 | 1 | 0.611 |
| semantic similarity similars | 2.963 | 1 | 0.085 |
| semantic similarity distants | 0.036 | 1 | 0.849 |
| triplet length | 3.389 | 1 | 0.066 |
| Right AIns E-field:Emotion_rating | 0.710 | 1 | 0.400 |
| Right AIns E-field:Social_rating | 0.292 | 1 | 0.589 |

Mixed-effect logistic regression model results of TMS E-field in right AIns and semantic ratings as predictors of accuracy to concrete triplets, where the last two rows represent the interaction between the magnitude of the E-field inside right AIns and respectively emotion and social rating. Significant effects are written in bold.

Chisq: Chi-squared statistic, Df: degrees of freedom
