# Supplementary material for: Emotional and Social Dimension of Abstract Concepts Meet with Interoception in Right Anterior Insula
Source: J Neurosci. 2025 Nov 21;46(2):e0238252025. doi: 10.1523/JNEUROSCI.0238-25.2025 (PMC12809663; doi:10.1523/JNEUROSCI.0238-25.2025)
Supplement: Figure 7-15 — Interaction between semantic ratings and E-field in left Anterior Insula as predictors of Accuracy of Concrete triplets. Mixed-effects logistic regression model results of TMS E-field in left AIns and semantic ratings as predictors of accuracy to concrete triplets, where the last two rows represent the interaction between the magnitude of the E-field inside left AIns and respectively emotion and social rating. Significant effects are written in bold. Chisq: Chi-squared statistic, Df: degrees of freedom. Download Figure 7-15, DOCX file. [file jneuro-46-e0238252025-s031.docx]

## Figure 7-15. Interaction between semantic ratings and E-field in left Anterior Insula as predictors of Accuracy of Concrete triplets.

|  | *Chisq* | *Df* | *p value* |
| --- | --- | --- | --- |
| (Intercept) | 295.956 | 1 | 0.000 |
| Left AIns E-field | 0.448 | 1 | 0.503 |
| Emotion_rating | 0.958 | 1 | 0.328 |
| Social_rating | 0.276 | 1 | 0.599 |
| semantic similarity similars | 2.952 | 1 | 0.086 |
| semantic similarity distants | 0.030 | 1 | 0.862 |
| triplet length | 3.314 | 1 | 0.069 |
| Left AIns E-field:Emotion_rating | 0.125 | 1 | 0.723 |
| Left AIns E-field:Social_rating | 0.059 | 1 | 0.809 |

Mixed-effects logistic regression model results of TMS E-field in left AIns and semantic ratings as predictors of accuracy to concrete triplets, where the last two rows represent the interaction between the magnitude of the E-field inside left AIns and respectively emotion and social rating. Significant effects are written in bold.

Chisq: Chi-squared statistic, Df: degrees of freedom
