# Supplementary material for: Emotional and Social Dimension of Abstract Concepts Meet with Interoception in Right Anterior Insula
Source: J Neurosci. 2025 Nov 21;46(2):e0238252025. doi: 10.1523/JNEUROSCI.0238-25.2025 (PMC12809663; doi:10.1523/JNEUROSCI.0238-25.2025)
Supplement: Table 3-1 — Single-subject MNI MAX PEAKS MNI coordinates of E-field maximum peak (MAX E-field) during left and right TMS, for each subject. Download Table 3-1, DOCX file. [file jneuro-46-e0238252025-s002.docx]

## Table 4-1. Interaction between semantic ratings and E-field in left Anterior Insula as predictors of Reaction times of Abstract triplets.

| *Model results* |  |  |  |  |  |  |
| --- | --- | --- | --- | --- | --- | --- |
|  | *Sum.Sq* | *Mean.Sq* | *NumDF* | *DenDF* | *F.value* | *p-value* |
| **Left AIns E-field** | **0.331** | **0.331** | **1** | **4523.185** | **5.602** | **0.018** |
| Emotion_rating | 0.222 | 0.222 | 1 | 103.405 | 3.751 | 0.055 |
| **Social_rating** | **0.312** | **0.312** | **1** | **103.486** | **5.281** | **0.024** |
| **semantic similarity similars** | **0.321** | **0.321** | **1** | **103.627** | **5.427** | **0.022** |
| semantic similarity distants | 0.000 | 0.000 | 1 | 104.848 | 0.001 | 0.972 |
| **triplet length** | **0.768** | **0.768** | **1** | **103.146** | **12.989** | **0.000** |
| Left AIns E-field:Emotion_rating | 0.197 | 0.197 | 1 | 4514.340 | 3.337 | 0.068 |
| **Left AIns E-field:Social_rating** | **0.263** | **0.263** | **1** | **4513.293** | **4.450** | **0.035** |

Mixed-effects model results of TMS E-field in left AIns and semantic ratings as predictors of (log-transformed) reaction times to abstract triplets, where the last two rows represent the interaction between the magnitude of the E-field inside left AIns and respectively emotion and social rating. Significant effects are written in bold.

Sum.Sq: Sum of squares, Mean.Sq: Sum of squares / degrees of freedom, NumDF: Degrees of freedom, DenDF: Denominator degrees of Freedom
