# Supplementary material for: Emotional and Social Dimension of Abstract Concepts Meet with Interoception in Right Anterior Insula
Source: J Neurosci. 2025 Nov 21;46(2):e0238252025. doi: 10.1523/JNEUROSCI.0238-25.2025 (PMC12809663; doi:10.1523/JNEUROSCI.0238-25.2025)
Supplement: Table 4-1 — Interaction between semantic ratings and E-field in left Anterior Insula as predictors of Reaction times of Abstract triplets. Mixed-effects regression model results of TMS E-field in left AIns and semantic ratings as predictors of (log-transformed) reaction times to abstract triplets, where the last two rows represent the interaction between the magnitude of the E-field inside left AIns and respectively emotion and social rating. Significant effects are written in bold. Sum.Sq: Sum of squares, Mean.Sq: Sum of squares / degrees of freedom, NumDF: Degrees of freedom, DenDF: Denominator degrees of Freedom Download Table 4-1, DOCX file. [file jneuro-46-e0238252025-s003.docx]

## Table 3-1. Single-subject MNI MAX PEAKS

| Left MAX | | | | | |  | | Right MAX | | |
| --- | --- | --- | --- | --- | --- | --- | --- | --- | --- | --- |
| x | | y | | z | |  | | x | y | z |
| -57 | | 31 | | 16 | |  | | 54 | 19 | 38 |
| -51 | | 25 | | 41 | |  | | 58 | 27 | 20 |
| -59 | | 24 | | 21 | |  | | 56 | 30 | 26 |
| -43 | | 29 | | 48 | |  | | 61 | 21 | 19 |
| -58 | | 33 | | 8 | |  | | 55 | 38 | 13 |
| -55 | | 40 | | 0 | |  | | 61 | 21 | 17 |
| 58 | | 28 | | 8 | |  | | 57 | 31 | 12 |
| -55 | | 34 | | -4 | |  | | 58 | 31 | 12 |
| -58 | | 20 | | 29 | |  | | 58 | 28 | 13 |
| -60 | | 17 | | 10 | |  | | 56 | 36 | 0 |
| -58 | | 32 | | 8 | |  | | 58 | 29 | 18 |
| -57 | | 33 | | 6 | |  | | 61 | 20 | 17 |
| -53 | | 39 | | 14 | |  | | 59 | 28 | 7 |
| -56 | | 36 | | 12 | |  | | 58 | 33 | 10 |
| -52 | | 43 | | 16 | |  | | 53 | 41 | 18 |
| -55 | | 29 | | 24 | |  | | 60 | 21 | 19 |
| -57 | | 35 | | 11 | |  | | 58 | 29 | 21 |
| -58 | | 57 | | 24 | |  | | 54 | 59 | 20 |
| -57 | | 34 | | 4 | |  | | 55 | 36 | 18 |
| -52 | | 22 | | 43 | |  | | 61 | 21 | 15 |
| -56 | | 35 | | 14 | |  | | 58 | 30 | 23 |
| -57 | | 33 | | 4 | |  | | 57 | 35 | 15 |
| -57 | | 35 | | 4 | |  | | 58 | 31 | 8 |
| -59 | | 27 | | 7 | |  | | 58 | 31 | 17 |
| -58 | | 32 | | 5 | |  | | 55 | 38 | 4 |
| AVERAGE | | | | | |  | | AVERAGE | | |
| -51.20 | 32.12 | | 14.92 | |  | | 57.48 | | 30.56 | 16 |

MNI coordinates of E-field maximum peak (MAX E-field) during left and right TMS, for each subject.
